# Male perceptions and experiences of weight loss initiatives: Considerations for sustainable lifestyle practices

**DOI:** 10.1371/journal.pone.0348158

**Published:** 2026-08-26

**Authors:** Mark Cortnage, Joseph Lillis, Justin Roberts

**Affiliations:** 1 Faculty of Health, Medicine and Social Care, School of Allied Health and Social Care, Anglia Ruskin University, Cambridge, United Kingdom; 2 Faculty of Science and Engineering, School of Psychology, Sport and Sensory Sciences, Anglia Ruskin University, Cambridge, United Kingdom; Innovative Aid, CANADA

## Abstract

This study employed a qualitative descriptive design to evaluate adult male perceptions and lived experiences of weight loss initiatives. In doing so, this study also aimed to understand the support mechanisms used by men when targeting weight loss, with a view to informing sustainable lifestyle practices. Following convenience sampling and informed consent, a total of 24 participants (age: 46 ± 10 yrs.; body mass: 91.1 ± 13.2 kg; height: 1.79 ± 0.06 m; BMI: 28.5 ± 4.4 kg·m^2^) took part in four focus group interviews (FGIs). These semi-structured interviews were undertaken using a standardised set of open-ended questions, with additional lines of enquiry employed where appropriate. Interviews were audio-recorded (MP3 format) and subsequently transcribed verbatim for thematic analysis. Using Braun and Clarkes six-stage model, structured thematic analysis highlighted four main themes: 1) male-specific perceptions of dieting and weight loss; 2) barriers to achieving dietary goals; 3) point-of-realisation and motivators for change; and 4) key factors for sustainable dietary control. Overall, the men perceived diets/dieting in a negative light, acknowledging the female-centric nature of weight loss programs, the need for male-specific narratives and the cyclical nature of dieting. This was coupled with barriers such as low motivation (and expectations of failure), lifestyle demands and misinformation (particularly around diets). Drivers for change focused on aesthetics over health factors and outlined an impulsiveness to diet. Three specific support systems were highlighted as potentially effective strategies to elicit sustainable engagement with dietary management. Practical implications from this study highlighted this “buddy-mentor-spousal” support system as fundamental to early and sustained success, with emphasis towards spousal support. Additionally, for sustainable lifestyle practices applicable to adult men, weight-loss initiatives should consider opportunistic simplicity coupled with quick-wins, early rewards and a tailored degree of social competitiveness around aesthetic gains, ahead of health-specific requirements.

## Introduction

Overweight and obesity are current primary health concerns within the UK population [1]. This is particularly relevant in adult males, with the overall proportion designated as overweight or living with obesity being 67% in 2022 [1]. Within specific age categories, prevalence of overweight/obesity has been reported greater for men aged 35–44 years (75%), 45–54 years (73%) and 55–64 years (80%) respectively [1]. This highlights the importance of both tailored and sustainable weight management solutions particularly for those approaching mid- to older ages.

Weight loss incentives often comprise of singular approaches including dietary restriction and physical activity. At the time of writing, there is significant focus on time-restricted dietary patterns [1,2], ketogenic/high-protein diets [3,4] and vigorous, intermittent exercise [5]. In many cases, such programmes provide a short-term mechanistic approach to “kickstart” metabolic processes pertinent to progressive metabolic flexibility and anthropometrical changes and fail to accommodate the complex interplay between lifestyle and behavioural modifications, cross-linked with energy and hormonal balance, genetics and environmental factors [6]. Therefore multi-component approaches are preferable for supporting weight loss and sustained management [7,8] with long term significant weight management deemed achievable when specific strategies that support lifestyle and behavioural change for adults are employed [9,10] particularly with male cohorts. The longer the period from weight loss through to maintenance, the increased likelihood of sustaining weight [10,11] thus underlining the need for prolonged support with goal setting, monitoring and improved self-perception and control as facilitators to meaningful change [12,13].

Several notable systematic reviews have been conducted exploring the experiences of successful weight loss maintainers [13,14]. However, the majority of the study participants were women highlighting the paucity of data exploring male perceptions and lived experiences of weight loss including the barriers and facilitators for successful weight maintenance. Exploration of male opinions around weight loss practices have yet to be conducted specifically around motivations for weight loss, efficacy, experiences and preferred approaches. Therefore, the aim of this study was to collect perceptions and lived experiences of weight loss and maintenance from adult men, to increase our understanding of male gendered perspectives around dieting.

## Materials and methods

### Ethical approval and participant recruitment

Following approval from the School of Psychology, Sport and Sensory Sciences Ethics Committee, Anglia Ruskin University (ETH2122−0617) participants were recruited through convenience sampling utilising multiple methods employing purposive and snowball sampling approaches. Recruitment consisted of word of mouth and personal contacts, through to study advertisement promoted locally (institutional intranet and public forum marketing) through to social media (Facebook/LinkedIn) promotion of the study. Additionally, study advertisement was promoted via local government authority health initiatives following gatekeeper approval. From this, 30 male participants volunteered to take part in one of four single-case focus groups.

For study inclusion, participants were required to be male, aged > 30 yrs, in good general health, and able to communicate in English. Participants were also required to have a lived experience of being overweight, having previously undertaken a weight-loss programme or were actively aiming to lose weight in the near future, and therefore willing and able to contribute to discussions around perceptions and experiences of weight loss initiatives within a semi-structured focus group discussion setting. Participants were excluded if they were: < 30 years, had an underlying medical condition that would make participation unsafe or inappropriate, or had no prior engagement with weight loss behaviours. Participants were also excluded if they were unwilling to be audio-recorded or unable to provide informed consent. From the initial cohort and following study inclusion matching, 24 participants (age: 46 ± 10 yrs.; body mass: 91.1 ± 13.2 kg; height: 1.79 ± 0.06 m; BMI: 28.5 ± 4.4 kg·m^2^) took part in the focus groups. Based on a criterion-based purposive sampling framework [15], and in line with previous research [16–18], sample size was considered satisfactory.

### Focus groups

This study employed a qualitative descriptive design using focus groups to explore men’s perceptions and experiences of weight loss initiatives. Based on previous work within our group [16] and recognising challenges faced with participant recruitment when exploring potentially sensitive topics, use of a focus group approach was deemed appropriate to provide an informal, yet safe environment for participants to proactively engage in open and honest discussion. Indeed, where there is peer support or discussion amongst participants with similar shared experiences this can provide deeper and richer qualitative information [19]. Therefore, following participant recruitment, four smaller focus group interviews (FGIs) were held in which more targeted discussion could be undertaken. Participants were provided with full study information sheets clearly outlining the aim and objectives of the study, as well as general expectations. Participation in the study was voluntary with freedom to withdraw without prejudice. FGIs were held in an informal meeting room at Anglia Ruskin University, Cambridge (in an open planned circle arrangement to foster equality, open communication and active listening throughout) or via Microsoft Teams (for those unable to attend face-to-face FGIs) with all participants providing written, informed consent prior to participation. All interviews were undertaken following the same set of pre-determined open-ended questions, with additional lines of enquiry included as appropriate. The interviews therefore followed a semi-structured approach providing flexibility for discussion. The focus group question framework (see S1 File) was developed by the researchers based on: i) review of existing literature on male engagement with weight loss programmes, relevant behavioural change strategies, relevant prevalence/ incidence data and gender-specific barriers; ii) theoretical considerations in including hegemonic practices, male help seeking behaviours, reports on lifestyle behaviour change, masculine norms, and both general and specific weight-management challenges/facilitators; and iii) the research teams prior work, which highlighted gaps in understanding men’s motivations and experiences with weight loss and management. Following initial team discussion (pertinent to the study objectives and key themes), open-ended questions were developed and refined to encourage more detailed responses and wider group discussion. This resulted in 3 distinct categories in logical order, and 14 prompt questions (with additional probing questions to further explore responses consistent with a semi-structured and flexible approach). The FGIs were conducted by experienced male researchers (doctoral level) to explore participant experiences, attitudes, perceptions, and beliefs surrounding weight loss and maintenance, with each one lasting approximately 60 minutes for standardisation. Each interview involved a lead moderator (MC) and objective moderator (JR/JL) to ensure consistency and rigor. All interviews were recorded in English using a TileRec Digital USB 3.0 Type C voice activated recorder, with MP3 files subsequently transcribed verbatim via an institution-approved provider for later thematic analysis and coding. Participant characteristics are shown in Table 1. Participants were compensated for their time with a standard gift voucher upon completion of the focus group.

**Table 1:** Participant characteristics for focus group interviews.

Focus group interview (FGI)	Participant	Age (years)	Ethnicity	Marital status	Education	Employment
FGI 1 In Person	1	≥60	White	M	Degree	FT
	2	≥40	White	M	Degree	FT
	3	≥40	White	M	Degree	FT
	4	≥50	White	NM	School	RET
	5	≥40	Asian	M	Degree	FT
	6	≥30	White	NM	PG	ST
FGI 2 Online	1	≥30	White	NM	Degree	FT
	2	≥50	White	M	PG	PT
	3	≥30	White	NM	PG	ST
	4	≥40	White	M	Degree	FT
	5	≥50	White	M	School	FT
	6	≥30	Asian	NM	PG	ST
FGI 3 Online	1	≥40	White	M	Degree	FT
	2	≥50	White	M	PG	FT
	3	≥40	White	M	Degree	FT
	4	≥40	White	NM	School	FT
	5	≥50	White	M	Degree	FT
	6	≥30	White	NM	Degree	FT
	7	≥40	White	M	PG	FT
	8	≥30	White	M	Degree	FT
FGI 4 In Person	1	≥50	White	NM	Degree	FT
	2	≥40	White	M	PG	FT
	3	≥50	White	NM	Degree	FT
	4	≥60	White	M	PG	PT

PG=Postgraduate; FT = Full-time; RET= Retired; PT = Part-time; ST = Student; M= Married; NM = Not married.

### Thematic analysis

Thematic analysis was employed to provide a structured approach to summarise similarities, differences and key features from the breadth of interview data. Braun and Clarkes six stage model [20] was employed to permit appropriate exploration of all necessary realms ([i] transcription/idea generation; [ii] code generation; [iii] theme searching; [iv] theme review/code alignment; [v] theme definition; [vi] theme report) before identifying specific markers of interest, theories and ideas prior to synthesising final conclusions. The first two stages, data familiarisation and the generation of initial codes was directly followed by stage three to five. Inductive and deductive coding approaches were initially conducted by two researchers (MC and JL) with second phase confirmation by a third researcher (JR). As such, this triangulation approach was employed to minimise risk of bias. Following organisation and clustering of codes, initial themes were scoped and refined. All authors were involved with final confirmation of assigned themes. The identification, reviewing and defining of themes refined the specific detail of each theme and therefore the underlying narrative embedded within the data [21]. The production of the report, stage six, moved beyond code and theme descriptors presenting an overarching message correlating directly with the primary aim of the study. By the fourth focus group, similar contexts, perspectives and experiences were noted and confirmed during the thematic analysis process inferring that saturation had likely been reached.

Overall rigor and trustworthiness of the qualitative findings were ensured through established criteria. Credibility (internal validity) was addressed by conducting multiple focus groups in a standardised, moderated and neutral manner, using confirmation questions or statements, a recognised analytical process, a triangulation approach during coding and team consensus on theme development. Transferability (external validity) was enhanced through transparent reporting of study procedures, including context, recruitment, inclusion/exclusion criteria, interview settings and development of the interview framework. Dependability (reliability) was demonstrated through use of a clear methodological process, a structured interview framework, inclusion of lead and objective moderators, and a recognised six-stage thematic analysis approach with triangulation to strengthen analytical rigor. Confirmability was achieved by minimising researcher bias through framework refinement using non-leading, open-ended interview questions, strategic moderator roles, and a validated thematic analysis process incorporating triangulation and open team discussion. Direct participant quotations were used to enrich and substantiate themes, demonstrating that findings were driven from collective shared experiences and perceptions rather than researcher assumptions.

## Results

From the thematic analysis and team consensus, 4 core themes and 12 sub-themes (ST) were uncovered as follows:

Theme 1: **Male-specific perceptions of dieting and weight loss**, including ST1 (Impressions of dieting), ST2 (Perceived effort and willpower), ST3 (Gendered perspectives), ST4 (The weight gain journey).
Theme 2: **Barriers to achieving dietary goals**, including ST1 (Lack of knowledge), ST2 (Lifestyle barriers).
Theme 3: **Point-of-realisation and motivators for change**, including ST1 (Perception and body/self-image), and ST2 (Motivation for change).
Theme 4: **Key factors for sustainable dietary control**, including ST1 (“The buddy”), ST2 (“The mentor”), ST3 (“The spouse”) and ST4 (Additional supportive factors).

Further assessment of the 4 core themes is demonstrated below, along with supporting participant quotations (and highlighted keywords) as pertinent. A summary of core themes, sub-themes and representative quotations is also shown in Table 2 at the end.

**Table 2:** Overall summary of the core themes, sub-themes and representative quotations from the thematic analysis.

Core theme	Sub-theme	Representative quotations
**Male-specific perceptions of dieting and weight loss**	Impressions of dieting	“I associate diet with dieting as having negative connotations”
	Perceived effort and willpower	“There has to be that willpower, whatever it is as soon as I start to fail I just give up mentally”
	Gendered perspectives	“I believe that women to look at dieting a bit differently from men”
	The weight gain journey	“Before you know where you are, you’re back at the other end of where you want to be”
**Barriers to achieving dietary goals**	Lack of knowledge	“There is so much misinformation out there about what makes a successful diet”
	Lifestyle barriers	“Other complications of life come into that”
**Point-of-realisation and motivators for change**	Perception and body/self-image	“I’m thinking, people are looking at me thinking I’m fat again”
	Motivation for change	“I never look at weight it’s how aesthetically you look that’s what I go on”
**Key factors for sustainable dietary control**	“The buddy”	“Surround yourself with people who are on the same mission”
	“The mentor”	“Because it’s always nice for someone to check in on you”
	“The spouse”	“For me, it was having the support of my wife. Without that, I wouldn’t have done it”
	Additional supportive factors	“It needs to be simple; it can’t be something that you have to think about and takes up too much time because I wouldn’t bother”

### Theme 1: Male-specific perceptions of dieting and weight loss

#### ST1 Impressions of dieting.

Collated findings from the FGIs outlined that on the whole men perceived dieting, and associated connotations including weight loss, in a generally negative manner associated to the short-term perception of dieting, particularly the restrictive nature of the approach. This was expressed by participants with the following examples:

- “I think diets are often a **short-term crash programm**e and it’s very difficult to sustain those” (FGI 2)
- “I associate diet with dieting as having **negative connotations**…my perception is that diets are **not successful”** (FGI 3)

This essentially implied that strategies to support men should not emphasise or focus on terminology associated with diet or restriction. In addition, the negative connotations perceived by men around diets and dieting, may inadvertently lead them to be automatically put off by a weight loss programme (which was noted as being associated with denial of foods or a lifestyle they enjoy), potentially resulting in a rebellious approach to such initiatives. Expressed consistently across all focus groups, this was portrayed by participants as follows:

- “I think diet is a very negative word because as soon as you mention the word diet everybody automatically thinks **restriction, restriction, restriction”** (FGI 2)
- “When I’m looking at doing any kind of restrictions or dieting **a part of me kind of rebels** against it as it were” (FGI 3)
- [Diets involve] “**Denying yourself** certain things” (FGI 4)

Despite these negative perceptions, across the focus groups participants alluded to (with a degree of self-pride) the number of diets they had attempted. It was therefore noteworthy that whilst diets were perceived as wholly negative, there appeared to be perceived merit in accumulating dietary attempts as a need for personal challenge. This was expressed as follows:

- “**I’ve pretty much been yo-yo dieting since I’m 18** up and down up and down, so I’ve been in that situation many times” (FGI 2)
- “**I’ve done south beach**…I tried Lighter Life, that gave me stomach aches, so did SlimFast… I did Slimming world, but I don’t like the Americanised, yeeha meetings…” (FGI 4)
- “**I’ve done the 5:2 intermittent** 500 calories in a day isn’t really good, I was thinking about doing the 16:8…I’ve tried **vegetarian diet but I gained on that one**” (FGI 4)

Fundamentally these insights highlighted the often-poor efficacy associated with generic, commercial programmes, which participants appeared to perceive as a type of challenge. If that challenge was achieved this equated to weight loss (sustainable or not); however, if unsuccessful, could reinforce their negative views of dieting. Furthermore, opinions suggested that diets are cyclic in nature consisting of sustained periods of weight gain intermitted with periods of dieting, further reinforcing the negative connotations associated with dieting and weight loss. Therefore, for weight loss initiatives to be both successful and sustainable, terminology should target away from “diets” and “dieting” and positively phrased towards a form of personal challenge.

#### ST2 Perceived effort and willpower.

Following thematic analysis, it was evident that there was pertinent discussion surrounding the self-discipline and effort required as well as the motivation to engage in such programmes, supporting ST1. The perceived effort required to undertake weight loss initiatives was raised across all focus groups and acknowledged by the majority of participants. It was therefore evident that this was a regular factor impacting programme engagement, as noted below:

- “It’s finding that **motivation to actually get on and do it**. And I need to be really focused on something to be able to do it on my own” (FGI 1)
- “The issue with a diet. Over the years I’ve actually **struggled**” (FGI 3)
- “There **has to be that willpower**, whatever it is as soon as I start to fail I just give up mentally and then it all falls apart and then a year later I try and gain and it’s just a cycle” (FGI 4)

Furthermore, the men demonstrated that even the smallest of perceived negative encounters when undertaking a weight loss programme such as food temptations or tasks requiring effort, could act to disproportionately demotivate male participants in future attempts.

- “**Temptation** is normally the thing that breaks you” (FGI 1)
- “Even the **slightest set back** can bring the whole thing crashing down” (FGI 1)
- “**Now calorie counting, I’m not going to do** it just because I can’t be arsed” (FGI 2)

It was therefore observed that the perceived effort, willpower or motivation needed to engage in traditional weight loss or dieting programmes was considered a central factor in poor success in the men interviewed, in some cases aligned with perceived failure. This highlighted that longer term initiatives need to consider factors which are both easy and achievable, whilst building in appropriate coping strategies to offset the potential for relapses or failure.

#### ST3 Gendered perspectives.

It was evident that men identified with a male gendered narrative when it comes to dieting and/or weight loss. A proposition raised initially by one participant in FGI 2, but subsequently endorsed across other participants, posited a gendered-binary perspective suggesting that women utilise “dieting” as a lifestyle factor, which contrasts with male-centric competitive sporting approaches. He did accept that not all men prefer sport or exercise to lose weight and highlighted the need for alternative approaches:

- “I think a lot of men, if you like sport, because this is okay if you’re doing it with people who are active….. I’ve done a lot of sport but there’s an awful lot of blokes out there that don’t do sport so you need to try and find another way to **challenge them** to realise yes, it’s not a diet **you’re not a woman just accept the fact** you’re a bit overweight and this is the way to do it” (FGI 2)

Clarification as to what the participant meant when suggesting that women diet, led to an open discussion exploring how men perceive weight and dieting in comparison with women. It was noted that some participants felt that dieting was a common approach for women and often discussed by them, particularly around weight and aesthetics, amplified by social media pressure. The negativity expressed by the men inferred that they were less inclined to be drawn into the dieting culture. This view was shared across the majority of participants, demonstrated as example:

- “I believe that women to **look at dieting a bit differently from men** and potentially different reasons” (FGI 2)
- “I feel **women pay more attention to their body**, the size of their body, the shape of their body compared to men” (FGI 2)
- “When we go down to the pub or socialise, we’re **not necessarily talking** about our weight or our appearance as such” (FGI 3)

The role of social media and advertising in reinforcing this perception were also noted as part of this theme:

- “When you look at articles if you go online and they used to have slimming stuff on TV would always have women so there was always an ad there that **women need to look slim”** (FGI 2)
- “Women it’s **part of that conversation** because now it’s **social media pressure**. And it’s there in the magazines and stuff, whereas it’s **not the topic of conversation primarily for men**. And I think it’s that whole culture change” (FGI 3)

These insights highlighted that for weight loss initiatives to be successful for men, a male gendered narrative should be employed, potentially focusing away from connotations of “diet” and being “slim” and more towards a positive challenge, which for some could involve sport or exercise.

#### ST4 The weight gain journey.

Throughout the focus group discussions there was also reference to the men’s relationships with food, particularly the cyclic nature of dieting. Men outlined a pervasive draw towards prior dietary habits and their relationship with food as a key issue with weight loss initiatives; as example this was expressed by one participant as:

- “The problem is me; the problem is the background. **My relationship to food**, the way I’m eating when I’m not dieting” (FGI 1)

Another participant, who mentioned that both “culture” and the “root cause” requires tackling, provides further clarity as to the superficial nature of dieting (as well as reaffirming that diets are a temporary approach), which in turn leaves the dieter susceptible to relapse.

- “So, if I lose weight and become not big. I then go, right my diet over I’ll return to my normal way of eating. But my normal way of eating made me big. So, I’m going to be big again aren’t I. So, you really have to tackle the culture, **the root cause** and not the symptoms” (FGI 1)

This weight gain journey was consistently expressed across all four groups, with the majority of participants describing a cyclic return to prior habits, as follows:

- “I’d lost my weight and then I thought I’ll have a doughnut today. And then one doughnut became two... and before you know where you are, **you’re back at the other end** of where you want to be” (FGI 1)
- “So, you can do that for a certain amount of time, but eventually, something will trigger it, or it will creep back and you will **end up on the same kind of weight** I’ve always been” (FGI 1)
- “I got down to 12 stone with that and as soon as I stopped **I went all the way back up**” (FGI 4)
- “Everything I’ve tried as soon as you stop doing it **and go back to where you were** it has to be a lifestyle change not a diet” (FGI 4)

It was therefore felt that men’s prior experiences had often led to the fundamental belief that diets are unlikely to be successful based on the negative weight-loss-weight-gain journey, and that having a better relationship with food could likely be a more sustainable strategy.

### Theme 2: Barriers to achieving dietary goals

When asked about the barriers men experienced as being fundamental to dietary failure, it was outlined that poor knowledge and information (or rather misinformation) led to confusion which in turn may obscure key, official dietary recommendations. This led to ST1 (lack of knowledge) below. We further uncovered as to how a busy working schedule can disrupt commitment to a dietary strategy (ST 2 lifestyle barriers). This was one of the most consistently raised barriers, noted across all focus groups.

#### ST1 Lack of knowledge.

It was evident from discussions that men perceived that having the right knowledge and information was important in creating the environment required for successful weight loss. This was noted as follows:

- “Knowledge is the biggest one, because there is **so much misinformation** out there about what makes a successful diet” (FGI 1)
- “I think **knowledge is the key here**. Knowing processed food is especially bad for you” (FGI 1)
- “One of the things which is a typical male way of living is we know everything we don’t like being told what to do, we don’t like to admit that **we don’t know** what we’re talking about” (FGI 2)
- “I did MyFitnessPal …. and that was an absolute pain... for me because **I’ve got no idea about what calories are or anything…** I completely rely on [my wife] so what she used to do was cook the dinner, tell me what was in it and then half the time as I’m trying to find everything and put it in …my phone” (FGI 4)

The last comment, aside from outlining a lack of knowledge with calories, suggested a dependency on the spouse to both prepare food and support the participant’s dieting attempts. Spousal support is further outlined in Theme 4, ST3. This sub-theme highlights the need for accurate dietary information which needs to be easy to understand and follow. Whilst partly expressed by participants (via spousal support), other forms of authoritarian or expert opinion may be important in reducing the negative connotations of misinformation.

#### ST2 Lifestyle barriers.

Following on from the prior discussion, we also uncovered how lifestyle factors, in particular working patterns, can be disruptive and could eventually undermine adherence to a dietary regime. Occupational and lifestyle demands were raised as significant barriers across three of the four groups, particularly among full-time workers.

- “You come home from work when you sit down because I’ve been working for eight or nine hours doing a physical job... and I don’t want to go out and do stuff, so you want to eat the simplest things which is kind of pasta or whatever **without realising the effect**” (FGI 2)
- “So I use to do long 12-hour shifts where you would **struggle to be able to eat on the shift** regardless of what you were trying to eat. And then at home, you’re exhausted afterwards. **You don’t want to cook particularly good things.** I’ve never found salads and fruit items particularly filling. There might be a psychological factor with that. But I would try and eat something healthy like baked potatoes, beans and a huge salad with it. Build up on the good bits with it. **Half an hour afterwards I’d be sitting there going I’m starving**” (FGI 3)
- “Other **complications of life** come into that, such as what my wife eats” (FGI 4)
- “That’s **partly what my lifestyle issue is** I’m out coaching tonight and I’m not going to get back until half eight and then we’re going to eat then” (FGI 4)

These insights highlight the need for weight loss initiatives to focus on “simplistic opportunity”; that is, tasks requiring minimal effort and time, which can be easily accommodated as part of a busy lifestyle. Based on participant discussions this not only needed to involve clear and correct information, but easy to manage and practical strategies built within typical habitual lifestyles (not as additional requirements).

### Theme 3: Point-of-realisation and motivators for change

All the men had a history of weight gain, and therefore FGI questioning further explored men’s perceptions and feelings regarding body and self-image, as well as how they felt they were perceived by others. This theme also explores participant experiences and the point when they felt the need to act to address their increasing weight. Although there were overlapping sub-themes, they were distinguishable in that ST1 (Perception of body/self-image) referred to the level of dissatisfaction with oneself and the point of motivation to change, whilst ST2 (Motivation to change) related to self and external perceptions of image deemed important enough or a “threshold” to trigger action.

#### ST1 Perception of body/self-image.

Negative body image was expressed with persistent emotional intensity across the focus groups, with most participants able to recall a specific triggering moment where realisation or self-awareness acted as a key component for personal change. Early on it was made clear to the researchers that the men regularly observed themselves and were overwhelmingly shameful in nature, outlining a negative aesthetic perception of themselves, which in turn impacted self-confidence. The majority of the men were able to clearly recall, often in detail, these negative feelings prior to attempting weight loss.

- “It’s **an embarrassment** because I’m thinking, people are looking at me thinking I’m fat again **I can’t believe I’m the size I am**” (FGI 2)
- “Looking in the mirror and just feeling **absolutely disgusted with myself**” (FGI 3)
- “I actually do like to wear suits and the suits I wear now it’s **not flattering anymore**” (FGI 3)
- “I walked past a mirror and thought, you’re a fat bastard to be perfectly blunt. A mirror, no T-shirt on and I just thought, Jesus that’s **the biggest I’ve ever been**” (FGI 4)

#### ST2 Motivation for change.

Body image and aesthetic dissatisfaction were clearly noted as dominant motivators raised spontaneously by most participants. However, throughout the open discussions, participant responses also focussed more towards the triggers to action and uncovered two core areas of interest invoking motivation for change: aesthetic appeal and personal identity. Although a different perspective and acknowledging the overlap with the previous sub-theme (referring to the level of dissatisfaction with oneself), the following sub-theme related more to self and external perceptions of image and personal identity. Responses were overwhelmingly related to negative body/image, and it was made apparent that these perceptions, induced a point in which “enough was enough” and culminated in positive action, albeit often impulsive or sudden.

- “I think for me if I’m completely honest **I needed the shame**, if that makes sense” (FGI 1)
- “It was an **identity** thing… I didn’t want to be like this” (FGI 2)
- “So, for me personally it wasn’t about weight, I never look at weight **it’s how aesthetically you look** that’s what I go on which is why this is massive for me... it’s not about weight I could weigh 100 kilos, 80 kilos it’s **how I think I should aesthetically look**” (FGI 2)
- “I **hated photos** of myself” (FGI 3)
- “So, there’s a kind of **mismatch** between your identity and who you think you are and who you actually are” (FGI 4)

Whilst aesthetic concerns dominated, the second largest motivator to change related to health and usually triggered meaningful action, due a prior health scare or concerns associated with health risk.

- “And then when they said I was diabetic type 2 or whatever it was. They wanted to put me on medication, and **I said no**” (FGI 1)
- “I had to have emergency open heart surgery. That was a **big wake up call**. But even more so, the **photo was the more, bigger prompt**” (FGI 1)
- “I could feel **something was not right**, and that triggered me to start my exercises” (FGI 2)
- “I had **no awareness** of how things were [in relation to a routine health check-up]” (FGI 2)
- “At home I happened to read a few cases of heart attack of people who are the same age as me and then **I really started thinking about it**, I still don’t know whether that was a fear of heart attack or something **that triggered me**” (FGI 2)

These insights highlighted that men were more governed by aesthetic appeal and personal identity (moving away from negative body image and aesthetic dissatisfaction) how they thought they should be looking. However, it appeared that a significant prompt (visual image of their physical self) or a potential health scare acted more directly as a “wake up call” to take meaningful action. These factors if utilised effectively could act to create acute awareness around their current circumstances and positively incentivize pro-action.

### Theme 4: Key factors for sustainable dietary control

Findings from the focus group discussion and thematic analysis uncovered potential approaches men viewed as being pivotal for continued engagement through to fruition of a positive dietary outcome. From the FGI’s it was interesting to note that very few of the men reported an approach to weight loss in the absence of peer support, particularly social engagement. Hence the concept of support structures dominated discussion, highlighting the potential need for social competitiveness along with peer or partner support. Other comments expressed by participants were also noted as being significant enough to impact engagement with overall, meaningful lifestyle change (often attempted through dietary intervention). As example, participants’ comments attributed their inability to exercise (recognised as an essential approach to weight loss) as a key factor, suggesting that in its absence, attempts to adopt positive lifestyle or dietary changes were likely to be futile, as noted below:

- “Because of my disabilities **I can’t do much in the way of exercise**” (FGI 4)
- “**I can’t put the miles in** I used to” (FGI 4).

Picking up on the preference for exercise, further questioning revolved around the methods that could be employed in order to engage the men with positive long-term lifestyle change. Here we uncovered the importance placed upon support structures, which in turn dominated discussion, with vehicles for continued motivation (and subsequent engagement) being competitive, supportive and guided in nature (ST1-ST3). Sub-theme 4 culminates in the men’s views as to the additional aspects that they felt were pivotal for dietary success.

#### ST1 “The buddy”.

The buddying system was the most frequently cited support structure, raised spontaneously across all focus groups and endorsed by the majority of participants. “The buddy” was commonly acknowledged as being an important approach to long-term engagement to change for men specifically. The idea of “competitive socialisation” was proposed as being central to sustained engagement in weight management programmes and focussed on male-centric social structures. This level of social accountability coupled with a sport or competitive element was highly noted across the FGIs.

- “Having a **check in buddy**, somebody on the diet who is motivated enough and says “Okay, do you want to check in buddy, do you want **somebody you could talk to**?” (FGI 2)
- “Surround yourself with **people who are on the same mission**” (FGI 2)
- “That would be certainly much easier if you were collaborating or even **competing with someone,** I think that would be an easier thing to do” (FGI 4)
- “But if I knew I was going to meet a bunch of people and run around the park together I’d probably **commit more to that**” (FGI 4)

#### ST2 “The mentor”.

Following on from the above sub-theme, in contrast to the need for social companionship (or a “buddy”) some interviewees suggested that direct mentorship would likely enhance accountability and therefore success. Depending upon the individual, some men preferred the need for a knowledgeable “mentor” to check on progress, provide advice and motivate; accordingly, differing slightly in context to a “buddy” system by re-positioning themselves into a subordinated role.

- “If you have a coach, if you hired a coach and contact them regularly and **they motivate you**, give you things to do and you are always checking in with them **so some sort of mentor**” (FGI 2)
- “I think having an **accountability partner** is key to weight loss” (FGI 2)
- “Because **it’s always nice for someone to check in on you** and make sure you are sticking to what you say you are going to stick to” (FGI 2)
- “Or just have a professional check-in. Have **someone external** who can see the progress in some way. I think that would probably be a good push” (FGI 3)

In part, this aligned with Theme 2 (ST1 Lack of knowledge) highlighting the need for guided support, monitoring or tracking potentially from a mentor with professional expertise (i.e., coach, healthcare professional) which could invoke a degree of trust and/or accountability.

#### ST3 “The spouse”.

The requirement for male-specific tailored support strategies, whether from a knowledgeable mentor or peer was a common suggestion and yet interestingly, most men also outlined how spousal support was pivotal for sustained success and adaptation to lifestyle change. The men were vocal when expressing how favourable spousal support had been and would often expand to the point of suggesting that the diet was largely orchestrated by the partner which inadvertently suggested that the male, again, acted in a subordinated role by having the spouse prepare meals and provide advice. This was expressed as follows:

- “**My wife structures our meal plan**, but she does like 10 days shopping in a row. And she’ll say to me, what do you want to eat on these days?” (FGI 1)
- “The snacks **she buys me** are apples rather than chocolates and stuff like that. That’s half the battle” (FGI 1)
- “**My wife helps me now a hell of a lot** because she kicks me in the arse when I start to go for the doughnuts again” (FGI 1)
- “**For me, it was having the support of my wife**. Without that, I wouldn’t have done it because she buys the food, she does the cooking in the evenings because I’m not home till late. If it wasn’t for her doing that then **I wouldn’t have got as far as I did**” (FGI 1)
- “(Use of a dietary app) because my wife uses it too which is **motivationally extremely helpful**” (FGI 2)
- “Because my **wife made me** a packed lunch” (FGI 4)

These insights also suggested that the men may not have fully immersed themselves within the dietary mindset (or did not feel a need to) and instead capitalised on the availability of spousal support to relinquish almost full control, including behavioural aspects, such as goal setting, self-motivation and self-control. As such, spousal support was deemed a key factor in sustainable practices which could be additionally beneficial in offsetting perceived setbacks.

#### ST4 Additional supportive factors.

Whist recognising the importance the men placed on peer support, this did not disregard other factors deemed important for dietary success, which varied amongst participants. The following views were raised across multiple groups, albeit with less consistency than the three primary support structures, and therefore grouped as additional supportive factors, including: clear and translatable knowledge; motivation through regular measurements; being realistic with goal setting; identifying root causes associated with weight gain; and keeping initiatives simple. Knowledge was a recognised as primary skill when embarking and engaging with a diet (including when attending group meetings) but also outlined the importance of social interaction, despite being initially dismissed by one recipient, as example:

- “Whilst I didn’t get much from the group aspect, one of the things I did appreciate from going to the groups was **the knowledge I got**. Even people that were doing their diet just like I was learning new things. And they were saying by the way, if you make your own sauces, it’s really easy to do. You just throw this, this and this in. Oh, that’s a good idea. You would get a little bit of knowledge, and you’d get that from like this little messenger group” (FGI 1)

Additional comments further outlined the array of opinions as to what could also be deemed as important when embarking on and sustaining a weight loss initiative for men:

- Motivation: “It’s all psychological as well, knowing that I’ve got to weigh. I don’t know, **there’s something about it**. It’s acknowledgement that you’ve got to weigh in” (FGI 1)
- Setting realistic achievable goals: “In 2019 I was 14 and a half stone in January. Then I got married 2020 January the second I was 10 and a half, so I’ve lost four stone. And for my frame and my height, I think 10 and a half stone **wasn’t really realistic**. But I managed to maintain that until COVID hit and had to stop going but I don’t think my target weight which I hit was realistic” (FGI 1)
- Identifying the root cause: “It’s knowing it’s a long journey and knowing that lesson I learned. You’re only tackling the symptoms; you need to **tackle the root cause**” (FGI 1)
- Simplicity: “**It needs to be simple**; it can’t be something that you have to think about and takes up too much time because I wouldn’t bother, I would go, “Oh yes this is fun” and three days later it’s no longer fun it’s a drag” (FGI 4)

## Discussion

The primary aim of this study was to understand male perceptions and lived experiences of weight loss initiatives, including support mechanisms when targeting weight loss. This may support future directions that enhance longer-term weight loss sustainability specific to this demographic which is often under-represented [22–24].

The first key finding from this study was that although the men recognised the positive associations between weight gain and health risk, and the need to manage their weight, the concept of diet and the primary method of weight control (dieting), was viewed negatively. Dieting conjured up feelings of restraint, requiring significant effort and willpower, and outlined restrictions on prior freedoms which in turn eroded willingness to engage. Perception of diet alone was firmly placed within the female domain, and yet for the men of this research, exercise was deemed to be the primary method for weight control, despite multi-component approaches highlighted as the most effective and preferred approach to weight loss [25].

Limiting factors contributing to the unsuccessful pursuit of dieting were primarily centred around the men’s own experiences of diets. It was often cited by the men that they had little inclination to persist with a diet, which required both significant effort and willpower, with factors such as temptation and disinclination as potential causes for disengagement. This pattern is consistent with the abstinence violation effect [26], whereby a single dietary lapse can manifest as total failure, potentially triggering complete disengagement rather than course correction. Added to this, participants also commented on the perceived expectation of longer-term failure based on prior experiences around weight-loss-weight-gain cyclic patterns. These insights clearly uncovered feelings of a general dislike of dieting by men, reinforced by prior experiences that induced barriers for future engagement. The frequently expressed short-term engagement also provided little time for acclimatisation with dietary principles and integration into daily lifestyle patterns. Additionally, several motivational components suggested as vital for meeting dietary outcomes, including the generation of self-efficacy, realistic goals, and internalisation of health messages [22,27] appear to be significantly undermined if expectations and chances of meeting dietary outcomes are eroded from the onset. These negative perceptions and experiences therefore highlight the importance of incorporating ecological momentary assessment (EMA) strategies to understand individual feelings, behaviours and environmental factors [26]. Such EMA approaches may be vital to identify and effect engaging and personalised coping strategies to enhance sustained practices and therefore longer-term success.

A consistent narrative associated with stereotypical gender was also evident throughout all conducted FGIs. It quickly became evident that the men perceived women as immersed within the cultural realm of dieting. Indeed, some males felt the presence of females and female centred strategies would inhibit the opportunity for engagement as dieting was deemed to be a feminised space where they felt self-conscious and uncomfortable. Participants noted a sense of “generalisation” in dietary approaches to weight management programmes which appeared to be largely directed towards or by feminine connotations associated with aesthetic appeal. Additionally, the sense of “restriction” combined with healthy eating was also perceived in a negative light in terms of associated short-term hunger and boredom.

This generalised perception of female-centric patterns associated with diet/weight programmes potentially overlooks the importance of masculinised preferences [23,24,28]. As such, men’s resistance to dieting can be understood through the framework of hegemonic masculinity and the dominant cultural norms that define what it means to “be a man” in a given social context [29]. Within this framework, dieting is considered a feminised behaviour and associated with restriction and aesthetic preoccupation upon which commercial programmes have traditionally posited as female spaces. For the men in this study, the dismissal of dieting functioned as an act of masculine identity maintenance, distancing themselves from a behaviour perceived as incompatible with their gender identity. The findings from this study therefore indicate that weight management programmes directed towards men should paradoxically focus away from “diet” aspects and are more likely to be successful if driven from a male-personalised narrative [30,31]. This could include narratives aligning more healthy dietary approaches and/or sport or physical challenges with associated masculinity (e.g., strength, stamina, integrity) [30,32,33]. This perspective also aligns with previous interventions reported elsewhere [16,34–36].

Paralleled with this, the researchers additionally observed that men recounted “serial dieting” attempts with a degree of self-pride warranting further consideration. It is feasible that this may reflect an identity-protective approach, whereby repeated failure is externalised and attributed to inadequacy of the programme rather than the individual, whilst continued attempts provide evidence of personal determination. If leveraged appropriately, this inherent competitive, self-orientation could represent a meaningful point of engagement for programme designers.

Whilst men’s perceptions and experiences of weight loss initiatives were largely negative, with dieting considered as being ineffective and expected to result in failure, poor knowledge and lifestyle challenges were also highlighted as additional barriers. Although some individuals did recognise both the importance of knowledge and the large amount of conflicting information (or misinformation), there was no evidence to suggest they had made attempts to explore further for clarity or to develop their own knowledge. In addition, often the opinions expressed by the men, suggested that they came from a position of experience and yet, further investigation outlined a lack of understanding around dieting such as the multiple approaches to weight loss, in particular nutrition. These experiences may have largely been pre-conceived ideas developed through for instance social interaction, which in turn acted as deterrents to engagement. Participant comments also outlined experiences around difficulty or inability in accommodating dietary approaches into their lifestyle [37], with working patterns having a significant negative impact on dietary efficacy which was compounded by insufficient cooking and food knowledge skills, leading to a sense of frustration as to both what to cook and the effort to create something that conforms with their dietary requirements. These barriers highlight the importance of both nutritional knowledge and applied food literacy for men (namely the capacity to translate dietary knowledge to plan and manage everyday food practices) [37]. Furthermore, some participants expressed how their spouse performed this translation suggesting that knowledge interventions should be oriented towards practical application rather than information transfer alone.

Despite a history of largely negative perceptions surrounding personal weight gain and viewing one’s body daily, the impetus to attempt a weight loss initiative would often stem from a single, significant moment in time. These instances, driven by appearance rather than health [16], were consistently recalled with emotive language and perceived by the researchers to include underlying negative, embarrassing or shameful feelings that impacted self-confidence and promoted sub-standard aesthetic perceptions of themselves when amongst others. Whilst such negative feelings have been reported to act as a barrier to male help-seeking behaviour [38,39], for the men in this study aesthetic improvement was, on the surface, demonstrated to be a more significant measure of success than health or health parameters. In fact, potential implications to health were only discussed as internal motivators by a few participants, with the emphasis often related to exercise and energy levels, as opposed to diet alone. Furthermore, an improvement in health was only considered once a “health related scare” had been encountered or noted in others of a similar age. Whether it was aesthetics or a “health related scare” that fuelled a change, the loss of identity or comparison to a former version of themselves often led the men to a threshold whereby “enough was enough”. Prior to this point, a general lethargy was outlined amongst the men, with a tolerated, underlying disquiet with the decline in health and/ or aesthetics which was insufficient to induce change. Subsequent action could be directly linked to an epiphany, however notably this change was often sudden, drastic, and thus unsustainable, ultimately resulting in little to no long-term effectiveness.

In contrast to the previously negative perceptions of diets and dieting being confined to a female-centric space around aesthetic preoccupation, this finding reveals a potential paradox. The same participants who aligned aesthetic concern with the female domain were themselves demonstrably motivated by body image dissatisfaction, recounting instances in which they had observed their reflection, objected to photographic representations of themselves, or noted that clothing no longer fitted. Body image was positioned rhetorically by the participants as a female preoccupation, and yet, in practice appeared to be a consistent and significant trigger for behavioural change (in one case more powerful than emergency surgery). The responses suggest that the leverage of aesthetic motivation among this cohort may be greater than their public discourse implied. Critically, the men appeared unaware of their positioning when their own motivation for change, derived from aesthetics, was expressed openly, and yet without the recognition that in doing so, the participants were themselves engaging with the very preoccupation they had distanced themselves from the female domain. We therefore suggest that the gendered boundary drawn around aesthetics was not a conscious act of masculine identity protection but rather an unconscious, cultural relationship in which the participants were complicit.

This tension is best understood through the concept of complicit masculinity; a position whereby men benefit from and broadly endorse hegemonic norms without fully embodying them [40]. What the researchers observed was a reframing, whereby the men consciously substituted aesthetic motivation with a more masculine narrative of health and performance. The practical implication is therefore more complicated than simply rebranding weight loss programmes in masculine language. If aesthetic motivation is simultaneously accepted and derided within the same cohort, male-targeted programme architecture could simply embed aesthetic outcomes within masculine-framed goals of performance and physical function. Aesthetic-performance masculine reframing has been observed in organically derived niche dietary communities rather than by design [41,42] and demonstrates how men reframed dieting as a masculine body project aimed at physical output whilst embracing aesthetic body work conveniently wrapped within hegemonic masculine norms. Yet this masculine reframing of dieting may come at a cost, through the development of fatphobic perceptions or persistent extreme leanness dependencies, underlining the idea that masculine reframing in commercial provision may introduce harmful dietary behaviours.

Our findings therefore highlight that for men, moving away from aesthetic dissatisfaction could act as a catalyst or motivational entry point for engagement [43,44] especially if brought to their awareness sooner. This could, for example, include early identification of individual factors associated with self-aesthetic appeal and/or aligned with masculine identity. However, importantly, such observations only consider motivational entry points. Therefore, sustainable lifestyle practices should consider these alongside other measures for coping or support structures to maximise programme adherence.

Vehicles for engagement were occasionally presented as positive and often included competitive behaviours that incentivised action and therefore transpired into relative success with dieting. Demonstrating weight loss improvements via incremental change, for example, a weight reduction from the previous measurement was shown to be a good motivator for continuation, and in fact the simplicity of competition, whether it was with oneself or as a wider group, was commonly noted by participants. Simplicity was a concept that was picked up in multiple contexts within the FGI’s, however this principle was always accompanied with competitive undertones, often referring to “incentives” or “challenges” and therefore perhaps indirectly could be associated with motivation to engage in a weight loss programme. Moreover, it was challenging to uncover the men’s feelings surrounding goal failure or a period of disengagement with research suggesting that common reasons for drop-out of weight management programmes for men, centre around a lack of autonomy and support [45]. Accountability was a common factor discussed, relating to a preference for a support structure. A socially competitive approach was cited as being useful for engagement, especially through “buddying” where training is undertaken together to reach an agreed goal. This initially appeared to conflict with research suggesting a masculinised requirement for autonomy [45] but further reflection by the researchers suggested that autonomy by the men was perceived as maintaining control rather than a full relinquishment of said control that would be expected within a structured commercial programme. The value of expert mentorship was also raised across three of the four groups, often distinguished from the buddy system by an emphasis on knowledge and expertise. The FGI’s were united in that the notion of either an informed professional, or a previous participant in the weight loss programme could provide mentorship and support when called upon. If identified early on, separate or combined buddying-mentor approaches could act not only as an EMA as part of an on-going monitoring and/or coping strategy but could also facilitate positive social competitiveness in line with meeting tailored aesthetic gains.

For those men in longer-term relationships, mention of spousal support was also highly noted, sharing how favourable female spousal support had been. In fact, many of the participants stated that their diet was largely orchestrated by their spouse with them acting in a participatory role and directed throughout the overall weight loss project. This aligns with previous research [44,46–48] and may stem from traditional or stereotypical marital roles, where the female is often responsible for buying and preparing food [49]. The researchers were led to a perception that men felt their female spouse, being immersed within the dieting realm and dieting (alone), were more knowledgeable and experienced within this realm and as such were able to relinquish control accordingly. If identified early on and leveraged correctly, exploring effective forms of spousal dietary support could facilitate longer term adherence with initiatives targeted at couples.

A consistent pattern across all three support structures was the men’s willingness to adopt a subordinated, accountable role within a trusted relational context; whether peer, expert or partner whilst expressing resistance to an equivalent level of accountability demanded by structured commercial programmes. This is particularly notable when framed against the broader masculine identity conflict highlighted throughout our data. Men who resisted dieting as a feminised, institutionally controlled endeavour were willing to relinquish agency when that authority was perceived as relationally legitimate as opposed to institutionally imposed. We posit that the distinction between identity-, lifestyle-, and context-congruent influences and institutional authorities perceived as controlling and incompatible with masculine self-concept is an important observation from this study.

In summary, the four themes reveal an underlying pattern: namely, a persistent tension between the men’s recognition of their weight-related health risk and their resistance to the interventions designed to address it. Further, this resistance appears centred around masculine identity norms; dieting is resisted when experienced as feminised, institutional or controlling, and accepted when reframed as a personal challenge or when engagement is justified through a trusted relational structure. The men’s aesthetic motivations were disavowed and reflect a form of complicit masculinity that should have direct implications for how programmes are both designed and communicated to men. Future interventions should leverage relational strength and masculine identity alignment without reinforcing potentially harmful masculine body ideals that can accompany masculine dietary culture.

### Limitations of the research

There are several limitations within the current study that are important considerations for contextualisation. Firstly, whilst sample size was deemed sufficient in line with previous literature, it was recognised that a larger cohort may have yielded additional findings. Although the FGIs provided novel insight into male perceptions and lived experiences of dieting, there was relatively limited diversity (mainly married, educated, white men) and therefore the potential for selection bias. As such, future research should consider perspectives representative of other demographics for more holistic understanding and wider generalizability. Additionally, whilst a recognised methodological approach was undertaken, the qualitative findings from FGIs largely involve self-reported perspectives and experiences. Other mixed method approaches or interventional tracking would therefore be merited in future research. Furthermore, the researchers did not delve into the specific constructs of digital applications or male-centric weight management programs, including use of personalised technology and behavioural coaching initiatives. It was also recognised that whilst the concept of dieting was discussed, we did not pursue specific aspects within the diet such as dietary types or diet quality. Future research should therefore consider such aspects with a view to personalising programs specific to male-centred objectives. It was also challenging to uncover the men’s feelings surrounding goal failure or a period of disengagement and this was understandable given the requirement to outline, among other men their inability to achieve a particular outcome. Individual interviews may have been more successful in uncovering the men’s feelings.

### Translational considerations

1. Specific to men, diets and dieting are typically perceived in a negative way, with lived experiences of expected effort and eventual failure. Use of ecological momentary assessment (EMA) strategies should be employed through identified support structures (see point 5) to understand individual challenges faced and create personalised coping strategies to enhance sustained lifestyle practices.
2. Men viewed dieting as a feminised space with most initiatives being considered “generalised” and “restrictive”. Strategies targeting male-centric weight management should therefore focus on male-gendered narratives aligning healthy dietary approaches and/or sport or physical challenges with associated masculinity and preferably in a socially competitive manner utilising early reward structures and “quick wins”.
3. Weight loss and health-specific requirements were considered less important than body image in the men interviewed, in paradoxical contrast to the off-putting perception that diets and dieting are typically confined to a female-centric space around aesthetic preoccupation. Early identification of individual factors associated with self-aesthetic appeal and/or aligned with masculine identity may be useful motivators to engagement. For some individuals, early parallel awareness around health-related risks or “scares” may also be an effective “point of realisation” approach to prompt motivation to change.
4. Practical application of both nutritional knowledge and applied food literacy were highlighted as key areas to incorporate into weight loss initiatives for longer term success. These could be subtly employed via “opportunistic simplicity” built into habitual lifestyle practices (e.g., wider choice of healthier on-the-go options or nutritious meal ideas within workplace settings) and through identified support structures (see point 5).
5. A novel aspect of this study was the identification of the importance of tailored interplay between buddy-mentor-spousal support as fundamental to early and sustained success, with the emphasis towards spousal support for those in longer-term relationships. If utilised effectively such support structures could be tailored to the individual and implemented to positively impact self-awareness, healthy eating knowledge and male-centric narratives, whilst facilitating coping strategies that may prevent or minimise programme withdrawal.
6. For healthcare professionals, policymakers or intervention designers, the findings from this study identified that male-gendered narratives should be considered in the planning phases, with due consultation with a diverse group of male stakeholders.
7. In driving sustainable lifestyle practices, healthcare professionals should aim to identify which support structure(s) an individual would benefit from the most based on personal requirements, compatibility with current lifestyle and/or existing relational trust.
8. Longer-term sustainable practices could include approaches with clear, accurate and concise information that builds knowledge and literacy around healthy eating and appropriate physical activity without conflicting with male-gendered narratives aligned with masculinity. Such approaches could aim to create a more positive “relationship with food” in supporting social or individual competitiveness (lifestyle activities or sport-related challenges), as opposed to negatively perceived restrictions or disliked tastes.

## Conclusions

This research proved instrumental in understanding male perceptions around the realm of dieting. Drivers for change stemmed primarily from aesthetics although a “health related scare” may also drive a person to adapt meaningful lifestyle changes. Consensus outlined a general uneasiness with prescribed dietary approaches which suggested a distancing from the female realm of dieting. However, discourse also suggested the men persistently engaged with diets to induce weight loss (albeit superficial in nature). A primary factor identified for longer-term dietary success was peer support with three primary relationships outlined: buddying, a mentor, or spousal support. Recruitment of spousal support was uncovered as likely the most effective method for supporting longer term engagement and goal-achievement, facilitated through the relegation of control to the spouse. Findings from this study highlight that sustainable lifestyle change specific to male populations need to be gender tailored and not generalised.

## Supporting information

S1 File Appendix showing general framework for focus group questions. (DOCX)
