# Supplementary material for: Male perceptions and experiences of weight loss initiatives: Considerations for sustainable lifestyle practices
Source: PLoS One. 2026 Aug 26;21(8):e0348158. doi: 10.1371/journal.pone.0348158 (PMC13514000; doi:10.1371/journal.pone.0348158)
Supplement: Supplementary file 1 [file pone.0348158.s003.pdf]

PLOS One

Male perceptions and experiences of weight loss initiatives: recommendations towards sustainable lifestyle practice.

--Manuscript Draft--

**Manuscript Number:**	PONE-D-25-58139R1
**Article Type:**	Research Article
**Full Title:**	Male perceptions and experiences of weight loss initiatives: recommendations towards sustainable lifestyle practice.
**Short Title:**	Male perceptions and experiences of weight loss initiatives
**Corresponding Author:**	Justin Roberts<br>Anglia Ruskin University<br>Cambridge, UNITED KINGDOM OF GREAT BRITAIN AND NORTHERN IRELAND
**Keywords:**	Weight loss; Perceptions; experiences; Lifestyle; Male; sustainability, diet, aesthetics
**Abstract:**	This study aimed to collect lived experiences and male perceptions of weight loss initiatives, as well as understand the support mechanisms used by men when targeting weight loss, with a view to informing sustainable lifestyle practices. Methods: Following informed consent, a total of 24 participants (age: 46±10 yrs.; body mass: 91.1±13.2 kg; height: 1.79±0.06 m; BMI: 28.5±4.4 kg·m2) took part in four focus group interviews (FGIs). All interviews were undertaken by experienced male researchers using a semi-structured approach following the same set of pre-determined open-ended questions, with additional lines of enquiry included as appropriate. Interviews were recorded with MP3 files transcribed for later thematic analysis and coding. Thematic analysis highlighted four main themes including: 1) male-specific perceptions of dieting and weight loss; 2) barriers to achieving dietary goals; 3) point-of-realisation and motivators for change; and 4) key factors for sustainable dietary control. Overall, the men perceived diet/dieting in a negative light, acknowledging the female-centric nature of weight loss programs, and the need for male-specific narratives as well as recognising the cyclic nature of dieting. This coupled with barriers of low motivation (and expectations of failure), lifestyle demands and misinformation (particularly around diets). Drivers for change were often focused on aesthetics over health factors and outlined an impulsiveness to diet. Three specific support systems were highlighted as potentially effective strategies to elicit sustainable engagement with dietary management.
**Order of Authors:**	Mark Cortnage, PhD
	Joseph Lillis
	Justin Roberts, PhD
**Response to Reviewers:**	Thank you for the email dated 27th February outline editorial and reviewer revisions for PONE-D-25-58139<br>Male perceptions and experiences of weight loss initiatives: recommendations towards sustainable lifestyle practice.<br>PLOS One<br><br>Outlined below are the full comments to all requests:<br><br>Authors replies to comments<br><br>Journal Requirement comments<br>1)Please ensure that your manuscript meets PLOS ONE's style requirements, including those for file naming.<br>Authors response: Thank you for the information. We have duly looked at both pdfs and fully reviewed/revised the manuscript in alignment with PLOS ONEs style requirements. This has also involved a complete revision for references.<br><br>2)When completing the data availability statement of the submission form, you

Powered by Editorial Manager® and ProduXion Manager® from Aries Systems Corporation

indicated that you will make your data available on acceptance. We strongly recommend all authors decide on a data sharing plan before acceptance, as the process can be lengthy and hold up publication timelines. Please note that, though access restrictions are acceptable now, your entire data will need to be made freely accessible if your manuscript is accepted for publication. This policy applies to all data except where public deposition would breach compliance with the protocol approved by your research ethics board. If you are unable to adhere to our open data policy, please kindly revise your statement to explain your reasoning and we will seek the editor's input on an exemption. Please be assured that, once you have provided your new statement, the assessment of your exemption will not hold up the peer review process.
Authors response: Thank you for this information. This statement will be revised during the resubmission process. As this was a qualitative study involving transcription of focus groups, full transcriptions can be made accessible.
3)Your ethics statement should only appear in the Methods section of your manuscript. If your ethics statement is written in any section besides the Methods, please delete it from any other section.
Authors response: Thank you for this information. We have removed the information at the end of manuscript and only kept a clear ethics statement in the Methods section as requested.

4) We note that this data set consists of interview transcripts. Can you please confirm that all participants gave consent for interview transcript to be published?
Authors response: Yes indeed, all participants provided consent for interview transcripts to be published. There are no potentially identifying information, and where only first names were mentioned, these were removed during the thematic analysis process so that individuals could not be identified in any context.
5)Please include captions for your Supporting Information files at the end of your manuscript, and update any in-text citations to match accordingly.
Authors response: Thank you for noting this, we have updated the manuscript accordingly.
6)If the reviewer comments include a recommendation to cite specific previously published works, please review and evaluate these publications to determine whether they are relevant and should be cited. There is no requirement to cite these works unless the editor has indicated otherwise.
Author response: we have noted this comment.

Response to reviewers:
Reviewer #1: Thank you for giving me the opportunity to review this paper. This study addresses a significant health issue, but there are several key concerns regarding the methodology:

1. Please clarify the specific type of qualitative study and provide justification for this choice.
Authors response: Firstly thank you for the review and commentary, which presented interesting and beneficial research team discussion. In constructing the paper, we aimed to be as concise as possible in the early stages, knowing that the nature of thematic analysis findings can be longer. In doing so, it is evident that we may have overlooked methodological detail which was indeed evident during the design and execution of the study. As the manuscript has been in submission since October, it has been a while since we have reviewed the paper so this has been a useful exercise in itself.

The study employed a qualitative descriptive study format using focus groups to explore mens perceptions and experiences of weight loss initiatives with the intention of informing practices which may be more sustainable and beneficial in the longer term. Based on previous work within our group, we also recognised the challenge faced when recruiting participants particularly when more sensitive topics are presented (such as weight loss). As such use of semi-structured interviews within focus group sessions was deemed appropriate in providing an informal, safe space for participants to openly and honestly engage. Indeed, topics which may be somewhat sensitive to discuss may be more easier to explore when there is peer support or similar shared experiences. The study type was based on previously published

Powered by Editorial Manager® and ProduXion Manager® from Aries Systems Corporation

	research by our group (reference 24) and follows similar guidelines and approaches outlined elsewhere (see: https://pubmed.ncbi.nlm.nih.gov/40374218/ and https://pmc.ncbi.nlm.nih.gov/articles/PMC10797665/). We have revised the beginning of the 'Focus groups' section in the methods to reflect revised modifications to the manuscript.<br><br>2. The study setting needs more detail. Please describe the geographic or contextual setting and explain how participants were recruited, what do you mean of via social media or advertisements?<br>Authors response: Again, thank you for the comment. It is important to note that we have followed approaches similar to other published articles (https://pubmed.ncbi.nlm.nih.gov/37339012/ , https://pubmed.ncbi.nlm.nih.gov/35010838/ , https://www.tandfonline.com/doi/full/10.1080/1612197X.2015.1114504), some of which do not go into as much detail (including one published in this journal: https://journals.plos.org/plosone/article?id=10.1371/journal.pone.0076089). However, in fully responding to this comment, as this was an exploratory study, we aimed to undertake local focus group interviews (specific to the Anglia Region, Cambridgeshire and Chelmsford where we are based in the UK). However, for those who could not attend the University, we also held an online Teams forum for practicality. All face-to-face interviews were held in a wide-spaced meeting room environment on the University campus, often used for general or public meetings. Interviews were undertaken with all participants sat in a circle, facing each other to foster equality, open communication, and active listening throughout. In all interviews members of the research team were also positioned amongst the participants within the circle. Participants were recruited via convenience sampling utilising several approaches, mainly word of mouth and personal contacts, alongside use of social media (researcher promotion via public advert on facebook or Linkedin). The same advert was also promoted via our local University intranet and media network (i.e. study adverts promoted internally via advertising screens and internal university marketing). In addition, study adverts were promoted via local government authority (Cambridgeshire Region) health initiatives following gatekeeper approval. Via adverts we employed purposive and snowballing sampling approaches. We have added content to the methods section to clarify these points.<br><br>3. In terms of participant selection, please provide comprehensive inclusion and exclusion criteria, as the current description is too brief.<br>Authors response: The initial information provided in the manuscript is correct based on the general remit employed during the ethical approval process. We purposely wanted to keep the inclusion remit broad so as not to restrict study participation. However, we have included additional information in the revised manuscript to further clarify study inclusion/exclusion in line with overall study conduct.<br>For study inclusion, participants were required to be male, aged > 30 years, in general good health, and able to communicate in English. Participants were also required to have a lived experience of being overweight, having previously undertaken a weight-loss programme or were actively aiming to lose weight in the near future, and therefore willing and able to contribute to discussions around perceptions and experiences of weight loss initiatives within a semi-structured focus group discussion setting. Participants were excluded if they were: < 30 years, had an underlying medical condition that would make participation unsafe or inappropriate, or had no prior engagement with weight loss behaviours. Participants were also excluded if they unwilling to be audio-recorded or unable to provide informed consent.<br><br>4. Regarding sampling, please detail the sampling framework and justify the sample size with relevant references.<br>Authors response: Our study employed a criterion-based purposive sampling framework. Participants were selected based on predefined characteristics that were directly aligned with the study aims and inclusion/exclusion criteria. Qualitative studies frequently employ purposeful sampling, a strategy in which participants are chosen based on their capacity to contribute detailed, experience-based information that aligns closely with the study's aims (Patton, 2002). Reference: Patton, M. 2002. Qualitative research and evaluation methods. 3rd Edition. Sage Publications; Thousand Oaks, CA. Sample size was based on prior experience

	(https://pubmed.ncbi.nlm.nih.gov/35010838/, incorporating 8 participants), and further justified through reference to numerous publications employing a similar methodological approach (e.g. https://www.mdpi.com/1660-4601/20/4/3320, n=13; https://www.mdpi.com/1660-4601/22/12/1796, 2 FGIs only and n=14; https://link.springer.com/article/10.1186/s12889-024-20677-5, n= 15 adults; https://www.tandfonline.com/doi/pdf/10.1080/17482631.2024.2406099, n=10). In the current study, we undertook several focus groups to ascertain theme saturation. The overall included sample size (n=24) is considered robust in alignment with other published research within this domain.<br><br>5. For data collection tools, please explain how the semi-structured interview questions were developed, their number, and whether any pilot testing was conducted.<br>Authors response: The semi-structured interview question framework (see S1 File) was developed to align closely with the primary aim of the study which was to explore male lived experiences, perceptions, motivations, and barriers related to weight-loss initiatives. The researcher's questions were developed based on the following:<br>•The existing literature on male engagement with weight-loss programmes, relevant behavioural change strategies, relevant prevalence / incidence data and gender-specific barriers.<br>•Theoretical considerations in including hegemonic practices, male help seeking behaviours, reports on lifestyle behaviour change, masculine norms, and both general and specific weight-management challenges / facilitators.<br>•The research team's prior work, which highlighted gaps in understanding men's motivations and experiences with weight loss and management.<br>During initial preparation, the research team discussed what the key objectives and themes were, and drafted open-ended questions to encourage more detailed responses and wider group discussion. Questions were informally piloted with academic colleagues during this initial preparation phase to ensure alignment with the underlying objectives of the study.<br>The resulting focus group framework consisted of a set of pre-determined open-ended questions, designed to prompt discussion around dieting experiences, perceived barriers, motivations for change, support mechanisms, and views on long-term weight-loss sustainability. The underlying framework comprised 3 general sections in logical order (views/opinions on weight management; knowledge and understanding around weight loss initiatives; and critical insights and thoughts towards longer term positive change), and 14 prompt questions. The researcher's accounted for the use of additional probing questions to further explore responses to improve depth of understanding, consistent with the semi-structured approach. Based on the above, we were able to ensure that the questions were grounded in evidence, relevant, and capable of eliciting rich experiential data.<br><br>6. Since the study employed focus group discussions, please justify why this method was chosen, especially considering the sensitive nature of the topic.<br>Author response: Focus group discussions were selected as the primary data collection method because they enabled the researchers to explore the experiences, social meanings, and the collective narratives surrounding weight loss among men >30 years. The researchers wished to understand male perceptions, motivations, and barriers to weight-loss engagement. Topics that are shaped not only by individual experiences and beliefs but by social norms, masculine identities, and peer group influences. Focus groups are well-suited to capturing these relational perspectives, as they provide an environment for participants to respond to, challenge, and build upon each other's perspectives. In doing so these help to reveal collective perspectives, beliefs, experienced and social norms detected via thematic analysis.<br>Based on prior work from this group, we were initially conscious of the sensitive nature of the topic, particularly from a male context which is somewhat under-represented. Indeed, findings from other studies highlight that sensitive topics may often be more freely disclosed or discussed within informal focus group settings where peer support or those having similar experiences become more comfortable to share perspectives and experiences<br>(https://www.tandfonline.com/doi/full/10.1080/13645579.2017.1281601).

Powered by Editorial Manager® and ProduXion Manager® from Aries Systems Corporation

	7. Concerning the study procedure, please elaborate on how social media was utilized, the recruitment timeline, the interviewers involved, the language used, and the overall recruitment and interviewing process.<br>Author response: As outlined within comment 2, participants were recruited via convenience sampling utilising several approaches, mainly word of mouth and personal contacts, alongside use of social media (researcher promotion via public advert on Facebook or Linkedin)[See comment 2 for further information]. In terms of social media, an online advert depicting the study aims and objectives, participants requirements and what to do next if interested was posted via the researchers own social media networks (e.g. Facebook, LinkedIn). This was part of the strategy to maximise reach within the local region via personal connections. The recruitment timeline was undertaken over a 7 week period preceding start of the first focus group. All researchers were involved in supporting and conducting the focus group interviews. MC is a qualitative public health researcher with considerable experience in this domain, particularly relevant to male participants and lived experienced. As such, MC was the key moderator for each focus group. Both JR and JL are experienced nutrition researchers, with experience in conducting research (including focus groups) pertinent to weight management and male participants. As such, JR acted as an objective moderator to ensure all themes/objectives were addressed. JL supported this role, and employed prompt or probing questions in line with the framework used to encourage further discussion. All focus group interviews were conducted verbally in English. For two of the FGIs, these were held face-to-face within the same University meeting room (purposefully designed to be an informal, relaxed, wide, open-planned setting). The room was set out in an open-planned, circular layout to encourage and facilitate open discussion. Following initial welcome and provision of name badges, the research team checked participants had read and fully understood the 'Participant Information Sheet' which overviewed the study in detail, emphasising that participation was voluntary, and that they could withdraw at any point without judgement. Following this, participants were required to complete written informed consent prior to starting the interview itself. All interviews lasted approximately 1hr and were audio recorded using a TileRec Digital USB 3.0 Type C voice activated recorder. The research team utilised the focus group questions framework as a means to provide logical structure and organisation to the interviews.<br><br>8. For data transcription and analysis, please clarify who handled transcription, whether translation was needed, and how potential biases were minimized.<br>Author response: in line with the policy of our Institutional ethics committee, when undertaking audio recorded interviews, audio files must be transcribed verbatim using a university-approved service. As such, once all focus groups were completed, all audio files (MP3) were uploaded to a university-approved provider, with word document transcripts provided to the research team following transcription completion. As all interviews were conducted in English, no translation was needed. When reviewing the transcripts, coding was initially conducted by two researchers (MC and JL) with second-phase confirmation by a third researcher (JR). As such, the use of duplicate coders, along with a second-phase confirmation process was employed as part of a triangulation process to minimise potential bias. Furthermore, by the fourth focus group, similar contexts, perspectives and experiences were noted by the research team, and confirmed during the thematic analysis process inferring that saturation had likely been reached.<br><br>9. Finally, regarding trustworthiness and rigor, please elaborate on how credibility, transferability, dependability, and confirmability were ensured.<br>Author response: To address this, we reverted back to early research by Lincoln and Guba where these dimensions are sourced (Lincoln, Y.S. and Guba, E.G. (1986), But is it rigorous? Trustworthiness and authenticity in naturalistic evaluation. New Directions for Program Evaluation, 1986: 73-84. https://doi.org/10.1002/ev.1427) .<br>In addressing credibility (internal validity) we employed several focus groups as a means to determine if similar experiences/perceptions were noted amongst the groups. As noted in previous comments, the use of focus groups was also pertinent to allow participants to respond to and build on each other's ideas, thus helping to reveal collective beliefs and social norms about topics meanwhile stimulating each other's experiences or perspectives. Focus groups were undertaken in a similar manner, utilising a facilitating lead moderator and objective moderator to ensure neutrality,

Powered by Editorial Manager® and ProduXion Manager® from Aries Systems Corporation

	consistency and rigor. Furthermore, in undertaking the focus groups, we also used confirmation questions or statements to ensure that the information provided was correct and clear. We also standardised the duration of each interview to ensure participants had a similar timeframe and context between focus groups.<br>In addition, we employed a recognised stage process (Braun and Clarke, 2006) to standardise the methodological approach, and used a clear triangulation approach during the thematic analysis coding process to minimise risk of internal bias and ensure multiple opinions were taken on board before arriving at a consensus.<br><br>In addressing transferability (external validity), we have provided comprehensive information relating to operational set up and delivery of the focus group interviews from context, recruitment/ inclusion/exclusion criteria, interview setting, and development of a focus group question framework. In doing so, our objective is to be as fully transparent in the process as possible for generalisability. We also noted earlier on that we employed a purposive sampling framework, aiming to also capture a range of ages (>30 years), educational backgrounds and marital status (see Table 1 in the manuscript). Within the focus group interviews we did notice that sometimes when one person would initiate a response to a moderator question, others would naturally follow without being prompted. Hence we felt that the interview process was on the whole fluid and natural. The use of prompt or probing questions was employed to either stimulate or delve into further discussion.<br><br>In term of dependability (reliability), we followed a clear and recognised process including development of an interview framework (which is transparent and clear), alongside use of a lead moderator and separate objective moderator to ensure standardisation. During analysis, we employed a recognised 6 stage model (Braun and Clarke, 2006) for thematic analysis and coding. In setting up the study, we decided to utilise a triangulation approach during coding to ensure rigor and minimise risk of internal bias. This involved initial independent coding and theme development by two members of the research team, and then separately a second-phase approach to independent coding/ theme development from the third member of the research team to assess both for consistency and internal checking in case anything had been missed.<br><br>In addressing confirmability (minimising researcher bias) we employed several approaches to ensure the research team did not unduly bias the process or findings and that the thematic analysis reflected the participants lived experiences and perspectives. This included development and refinement of a focus group question framework, including lead and objective moderator roles and use of non-leading open ended questions. Further, by employing a recognised thematic analysis process (Braun and Clarke, 2006), this minimises opportunity for internal bias. During the coding process we employed a clear triangulation process as previously noted. Furthermore, during the development of themes, we employed open discussion and shared observations within the research team to ensure that previous assumptions based on prior research were not directly influencing the findings. From the results section, we have included direct quotations from participants to fully enrich the nature of the themes presented, highlighting that underlying themes were driven from collective shared experiences and perceptions from the groups.
**Additional Information:**	
**Question**	**Response**
**Financial Disclosure**<br><br>Enter a financial disclosure statement that describes the sources of funding for the work included in this submission. Review the submission guidelines for detailed requirements. View published research articles from *PLOS ONE* for specific examples.	The author(s) received no specific funding for this work.

This statement is required for submission and **will appear in the published article** if the submission is accepted. Please make sure it is accurate.<br><br>**Funded studies**<br>Enter a statement with the following details:<br>• Initials of the authors who received each award<br>• Grant numbers awarded to each author<br>• The full name of each funder<br>• URL of each funder website<br>• Did the sponsors or funders play any role in the study design, data collection and analysis, decision to publish, or preparation of the manuscript?<br><br>Did you receive funding for this work?	
**Competing Interests**<br><br>Use the instructions below to enter a competing interest statement for this submission. On behalf of all authors, disclose any competing interests that could be perceived to bias this work—acknowledging all financial support and any other relevant financial or non-financial competing interests.<br><br>This statement is required for submission and **will appear in the published article** if the submission is accepted. Please make sure it is accurate and that any funding sources listed in your Funding Information later in the submission form are also declared in your Financial Disclosure statement.<br><br>View published research articles from *PLOS ONE* for specific examples.	The authors have declared that no competing interests exist.

Powered by Editorial Manager® and ProduXion Manager® from Aries Systems Corporation

**NO authors have competing interests**<br><br>Enter: *The authors have declared that no competing interests exist.*<br><br>**Authors with competing interests**<br><br>Enter competing interest details beginning with this statement:<br><br>*I have read the journal's policy and the authors of this manuscript have the following competing interests: [insert competing interests here]*<br><br>* typeset	
**Ethics Statement**<br><br>Enter an ethics statement for this submission. This statement is required if the study involved:<br><br>• Human participants<br>• Human specimens or tissue<br>• Vertebrate animals or cephalopods<br>• Vertebrate embryos or tissues<br>• Field research<br><br>Write "N/A" if the submission does not require an ethics statement.<br><br>General guidance is provided below. Consult the submission guidelines for detailed instructions. **Make sure that all information entered here is included in the Methods section of the manuscript.**	Anglia Ruskin University, Cambridge, UK - School of Psychology, Sport and Sensory Sciences Ethics Committee (ethics approval number: ETH2122-0617). Written, informed consent was obtained from all participants.

**Format for specific study types**<br><br>**Human Subject Research (involving human participants and/or tissue)**<br>• Give the name of the institutional review board or ethics committee that approved the study<br>• Include the approval number and/or a statement indicating approval of this research<br>• Indicate the form of consent obtained (written/oral) or the reason that consent was not obtained (e.g. the data were analyzed anonymously)<br><br>**Animal Research (involving vertebrate animals, embryos or tissues)**<br>• Provide the name of the Institutional Animal Care and Use Committee (IACUC) or other relevant ethics board that reviewed the study protocol, and indicate whether they approved this research or granted a formal waiver of ethical approval<br>• Include an approval number if one was obtained<br>• If the study involved *non-human primates*, add *additional details* about animal welfare and steps taken to ameliorate suffering<br>• If anesthesia, euthanasia, or any kind of animal sacrifice is part of the study, include briefly which substances and/or methods were applied<br><br>**Field Research**<br><br>Include the following details if this study involves the collection of plant, animal, or other materials from a natural setting:<br>• Field permit number<br>• Name of the institution or relevant body that granted permission	
**Data Availability**<br><br>Authors are required to make all data underlying the findings described fully available, without restriction, and from the time of publication. PLOS allows rare exceptions to address legal and ethical concerns. See the PLOS Data Policy and FAQ for detailed information.	Yes - all data are fully available without restriction

A Data Availability Statement describing where the data can be found is required at submission. Your answers to this question constitute the Data Availability Statement and **will be published in the article**, if accepted.<br><br>**Important:** Stating 'data available on request from the author' is not sufficient. If your data are only available upon request, select 'No' for the first question and explain your exceptional situation in the text box.<br><br>Do the authors confirm that all data underlying the findings described in their manuscript are fully available without restriction?	
**Describe where the data may be found in full sentences. If you are copying our sample text, replace any instances of XXX with the appropriate details.**<br><br>• If the data are **held or will be held in a public repository**, include URLs, accession numbers or DOIs. If this information will only be available after acceptance, indicate this by ticking the box below. For example: *All XXX files are available from the XXX database (accession number(s) XXX, XXX.).*<br>• If the data are all contained **within the manuscript and/or Supporting Information files**, enter the following: *All relevant data are within the manuscript and its Supporting Information files.*<br>• If neither of these applies but you are able to provide **details of access elsewhere**, with or without limitations, please do so. For example:<br><br>*Data cannot be shared publicly because of [XXX]. Data are available from the XXX Institutional Data Access / Ethics Committee (contact via XXX) for researchers who meet the criteria for access to confidential data.*<br><br>*The data underlying the results presented in the study are available from (include the name of the third party*	All relevant data are within the manuscript and its Supporting Information files

and contact information or URL).<br>• This text is appropriate if the data are owned by a third party and authors do not have permission to share the data.<br><br>* typeset	
Additional data availability information:	Tick here if the URLs/accession numbers/DOIs will be available only after acceptance of the manuscript for publication so that we can ensure their inclusion before publication.

Manuscript Click here to access/download;Manuscript;Manuscript.docx

Male perceptions and experiences of weight loss initiatives: recommendations towards sustainable lifestyle practice.

Mark Cortnage [1¶*], Joseph Lillis [2¶], Justin Roberts [2¶*]

[1] Faculty of Health, Medicine and Social Care, School of Allied Health and Social Care, Anglia Ruskin University, Cambridge, CB1 2LZ, UK

[2] Faculty of Science and Engineering, School of Psychology, Sport and Sensory Sciences, Anglia Ruskin University, Cambridge, CB1 1PT, UK

[*] Corresponding authors

Email: mark.cortnage@aru.ac.uk (MC); justin.roberts@aru.ac.uk (JR)

[¶] These authors contributed equally to this work

Abstract

This study aimed to collect lived experiences and male perceptions of weight loss initiatives, as well as understand the support mechanisms used by men when targeting weight loss, with a view to informing sustainable lifestyle practices. Following informed consent, a total of 24 participants (age: 46±10 yrs.; body mass: 91.1±13.2 kg; height: 1.79±0.06 m; BMI: 28.5±4.4 kg·m^2) took part in four focus group interviews (FGIs). All interviews were undertaken by experienced male researchers using a semi-structured approach following the same set of pre-determined open-ended questions, with additional lines of enquiry included as appropriate. Interviews were recorded with MP3 files transcribed for later thematic analysis and coding. Thematic analysis highlighted four main themes including: 1) male-specific perceptions of dieting and weight loss; 2) barriers to achieving dietary goals; 3) point-of-realisation and motivators for change; and 4) key factors for sustainable dietary control. Overall, the men perceived diet/dieting in a negative light, acknowledging the female-centric nature of weight loss

programs, and the need for male-specific narratives as well as recognising the cyclic nature of dieting. This coupled with barriers of low motivation (and expectations of failure), lifestyle demands and misinformation (particularly around diets). Drivers for change were often focused on aesthetics over health factors and outlined an impulsiveness to diet. Three specific support systems were highlighted as potentially effective strategies to elicit sustainable engagement with dietary management.

Introduction

Overweight and obesity are current primary health concerns within the UK population [1]. This is particularly relevant in adult males, with the overall proportion designated as overweight or living with obesity being 67% in 2022 [1]. Within specific age categories, prevalence of overweight/obesity has been reported greater for men aged 35-44 years (75%), 45-54 years (73%) and 55-64 years (80%) respectively [1]. This highlights the importance of both tailored and sustainable weight management solutions particularly for those approaching mid- to older ages.

Weight loss incentives often comprise of singular approaches including dietary restriction and physical activity. At the time of writing, there is significant focus on time-restricted dietary patterns (1,2), keto-genic/high-protein diets (3,4) and vigorous, intermittent exercise (5). In many cases, such programmes provide a short-term mechanistic approach to 'kickstart' metabolic processes pertinent to progressive metabolic flexibility and anthropometrical changes and fail to accommodate the complex interplay between lifestyle and behavioural modifications, cross-linked with energy and hormonal balance, genetics and environmental factors (6). Therefore multi-component approaches to weight loss are preferable for supporting weight loss and sustained management (7,8) with long term significant weight management achievable with specific strategies supporting lifestyle and behavioural change for adults (9,10) particularly with male cohorts. The longer the period from weight loss through to maintenance, the increased likelihood of sustaining weight (10,11) and underlines the need for sustained, prolonged support with goal setting, monitoring and improved self-perception and control as facilitators to meaningful change (12,13).

Several notable systematic reviews have been conducted exploring the experiences of successful weight loss maintainers (13,14). However, the majority of the study participants were women and underline the paucity of data exploring male experiences of weight loss and perceptions of the barriers and facilitators for successful maintenance of weight. Exploration of male opinions around weight loss practices have yet to be conducted specifically around motivations for weight loss, efficacy, experiences and preferred approaches. Therefore, the aim of this study was to collect lived experiences and perceptions of weight loss and maintenance from adult men, to increase our understanding of male gendered perspectives around dieting.

Materials and methods

Ethical approval and participant recruitment

Following University Ethics Committee approval (ETH2122-0617) participants were recruited through convenience sampling utilising multiple methods employing purposive and snowball sampling approaches between June-July 2022. Recruitment consisted of word of mouth and personal contacts, through to study advertisement promoted locally (institutional intranet and public forum marketing) through to social media (Facebook/LinkedIn) promotion of the study. Additionally, study advertisement was promoted via local government authority health initiatives following gatekeeper approval. From this, 30 male participants volunteered to take part in one of four single-case focus groups.

For study inclusion, participants were required to be male, aged > 30 yrs, in general good health, and able to communicate in English. Participants were also required to have a lived experience of being overweight, having previously undertaken a weight-loss programme or were actively aiming to lose weight in the near future, and therefore willing and able to contribute to discussions around perceptions and experiences of weight loss initiatives within a semi-structured focus group discussion setting. Participants were excluded if they were: < 30 years, had an underlying medical condition that would make participation unsafe or inappropriate, or had no prior engagement with weight loss behaviours. Participants were also excluded if they unwilling to be audio-recorded or unable to provide informed

consent. From the initial cohort and following study inclusion matching, 24 participants (age: 46±10 yrs.; body mass: 91.1±13.2 kg; height: 1.79±0.06 m; BMI: 28.5±4.4 kg·m^2) took part in the focus group. Based on a criterion-based purposive sampling framework (15), and in line with previous research (16-18), sample size was considered satisfactory.

Focus groups

This study employed a qualitative descriptive design using focus groups to explore men's perceptions and experiences of weight loss initiatives. Based on previous work within our group (16) and recognising challenges faced with participant recruitment when exploring potentially sensitive topics, use of a focus group approach was deemed appropriate to provide an informal, yet safe environment for participants to proactively engage in open and honest discussion. Indeed, where there is peer support or discussion amongst participants with similar shared experiences this can provide deeper and richer qualitative information (19). Therefore, following participant recruitment, four smaller focus group interviews (FGIs) were held in which more targeted discussion could be undertaken. Participants were provided with full study information sheets clearly outlining the aim and objectives of the study, as well as general expectations. Participation in the study was voluntary with freedom to withdraw without prejudice. FGIs were held in an informal meeting room at Anglia Ruskin University, Cambridge (in an open planned circle arrangement to foster equality, open communication and active listening throughout) or via Microsoft Teams (for those unable to attend face-to-face FGIs) with all participants providing written, informed consent prior to participation. All interviews were undertaken following the same set of pre-determined open-ended questions, with additional lines of enquiry included as appropriate. The interviews therefore followed a semi-structured approach providing flexibility for discussion. The focus group question framework (see S1 File.) was developed by the researchers based on: i) review of existing literature on male engagement with weight loss programmes, relevant behavioural change strategies, relevant prevalence/ incidence data and gender-specific barriers; ii) theoretical considerations in including hegemonic practices, male help seeking behaviours, reports on lifestyle behaviour change, masculine norms, and both general and specific weight-management challenges/facilitators; and iii) the research teams prior work, which highlighted

gaps in understanding men's motivations and experiences with weight loss and management. Following initial team discussion (pertinent to the study objectives and key themes), open-ended questions were developed and refined to encourage more detailed responses and wider group discussion. This resulted in 3 distinct categories in logical order, and 14 prompt questions (with additional probing questions to further explore responses consistent with a semi-structured and flexible approach). The FGIs were conducted by experienced personnel to explore male participant experiences, attitudes, perceptions, and beliefs surrounding weight loss and maintenance, with each one lasting approximately 60 minutes for standardisation. Each interview involved a lead moderator (MC) and objective moderator (JR/JL) to ensure consistency and rigor. All interviews were recorded in English using a TileRec Digital USB 3.0 Type C voice activated recorder, with MP3 files transcribed verbatim via an institution-approved provider for later thematic analysis and coding. Participant characteristics are shown in Table 1. Participants were compensated for their time with a standard gift voucher upon completion of the focus group.

Table 1. Participant characteristics for focus group interviews.

**Focus group**	**Age (years)**	**Ethnicity**	**Marital status**	**Education**	**Employment**
**Focus Group 1 In Person**	67	White	M	Degree	FT
	40	White	M	Degree	FT
	40	White	M	Degree	FT
	55	White	NM	School	RET
	40	Asian	M	Degree	FT
	31	White	NM	PG	ST
**Focus Group 2 Online**	38	White	NM	Degree	FT
	56	White	M	PG	PT
	30	White	NM	PG	ST
	43	White	M	Degree	FT
	56	White	M	School	FT
	32	Asian	NM	PG	ST

Focus Group 3 Online	41	White	M	Degree	FT
	54	White	M	PG	FT
	42	White	M	Degree	FT
	45	White	NM	School	FT
	58	White	M	Degree	FT
	36	White	NM	Degree	FT
	44	White	M	PG	FT
	32	White	M	Degree	FT
Focus Group 4 In Person	54	White	NM	Degree	FT
	48	White	M	PG	FT
	56	White	NM	Degree	FT
	60	White	M	PG	PT

PG=Postgraduate; FT = Full-time; RET= Retired; PT = Part-time; ST = student; M= Married; NM = Not married

Thematic analysis

Thematic analysis was employed to provide a structured approach to summarise similarities, differences and key features from the breadth of interview data. Braun and Clarkes six stage model (20) was employed to permit appropriate exploration of all necessary realms ([i] transcription/idea generation; [ii] code generation; [iii] theme searching; [iv] theme review/code alignment; [v] theme definition; [vi] theme report) before identifying specific markers of interest, theories and ideas prior to synthesising final conclusions. The first two stages, data familiarisation and the generation of initial codes was directly followed by stage three to five. Coding was initially conducted by two researchers (MC and JL) with second phase confirmation by a third researcher (JR). As such, this triangulation approach was employed to minimise risk of bias. All authors were involved with final confirmation of assigned themes. The identification, reviewing and defining of themes refined the specific detail of each theme and therefore the underlying narrative embedded within the data (21). The production of the report, stage six, moved beyond code and theme descriptors presenting an overarching message correlating directly with the primary aim of the study. By the fourth focus group, similar contexts,

perspectives and experiences were noted and confirmed during the thematic analysis process inferring that saturation had likely been reached.

Results

From the thematic analysis, four core themes and 12 sub themes (ST) were uncovered as follows:

Theme 1: **Male-specific perceptions of dieting and weight loss**, including ST1 (impressions of dieting), ST2 (perceived effort and willpower), ST3 (gendered perspectives), ST4 (the weight gain journey).

Theme 2: **Barriers to achieving dietary goals**, including ST1 (lack of knowledge), ST2 (lifestyle barriers).

Theme 3: **Point-of-realisation and motivators for change**, including ST1 (perception and body/self-image), and ST2 (motivation for change).

Theme 4: **Key factors for sustainable dietary control**, including ST1 ('The buddy'), ST2 ('The mentor'), ST3 ('The spouse') and ST4 (additional supportive factors).

Further assessment of the 4 core themes is demonstrated below, along with supporting participant quotations as pertinent.

Theme 1: Male-specific perceptions of dieting and weight loss

ST1 Impressions of dieting

Collated findings from the FGIs outlined that on the whole men perceived dieting, and associated connotations including weight loss, in a generally negative manner associated to the short-term

perception of dieting, particularly the restrictive nature of the approach. This essentially implied that strategies to support men should not emphasise or focus on terminology associated with diet/restriction. This was portrayed by participants as follows:

- ‘I associate diet with dieting as having **negative connotation**s…my perception is that diets are **not successful**.**’**
- ‘I think diet is a very negative word because as soon as you mention the word diet everybody automatically thinks **restriction, restriction, restriction**.**’**
- ‘I think diets are often a **short-term crash programm**e and it's very difficult to sustain those’
- [Diets involve] ‘**Denying yourself** certain things.’
- ‘When I’m looking at doing any kind of restrictions or dieting **a part of me kind of rebels** against it as it were.’

Despite these negative perceptions, several participants, alluded to (with a degree of self-pride) the number of diets they had attempted. It was therefore noteworthy that whilst diets were perceived as wholly negative, there was perceived merit in accumulating dietary attempts as a need for personal challenge. Fundamentally this highlighted, to the researchers, the often-poor efficacy associated with more generic, commercial programmes but which is perceived by the participants as a challenge, that if achieved, equates to weight loss (sustainable or not); and if unsuccessful, could act to prove that their negative portrayals of dieting are justified. Furthermore, opinions suggest that diets are cyclic in nature consisting of sustained periods of weight gain intermitted with periods of dieting.

- ‘I’ve done south beach…I tried Lighter Life, that gave me stomach aches, so did SlimFast… I did Slimming world, but I don’t like the Americanised, yeeha meetings…’
- ‘I’ve done the 5:2 intermittent 500 calories in a day isn’t really good, I was thinking about doing the 16:8…I’ve tried vegetarian diet but I gained on that one.’
- ‘**I've pretty much been yo-yo dieting since I'm 18 up** and down up and down, so I've been in that situation many times.’

ST2 Perceived effort and willpower

There was pertinent discussion surrounding the self-discipline and effort required as well as the motivation to engage in such programmes, supporting ST1. With the large number of men acknowledging these statements, it was evident that this was a regular factor impacting programme engagement. Furthermore, the men demonstrated that even the smallest of perceived negative encounters when undertaking a weight loss programme, could act to disproportionately demotivate male participants in future attempts.

- '**Temptation** is normally the thing that breaks you.'
- 'Even the **slightest set back** can bring the whole thing crashing down.'
- 'Now calorie counting, I'm not going to do it just because I can't be arsed.

ST3 Gendered perspectives

It was evident that men identified with a male gendered narrative when it comes to dieting and/or weight loss. A proposition by one participant presented a gendered-binary perspective suggesting that women utilise 'dieting' which contrasts with male-centric competitive sporting approaches. He did accept that not all men prefer exercise to lose weight and the need for alternative approaches.

- 'I think a lot of men, if you like sport, because this is okay if you're doing it with people who are active or been active historically…done a lot of sports, I've done a lot of sport but there's an awful lot of blokes out there that don't do sport so you need to try and find another way to **challenge them** to realise yes, it's not a diet **you're not a woman just accept the fact** you're a bit overweight and this is the way to do it, a way to do it not the way.'

Clarification as to what the participant meant when suggesting that women diet, led to an open discussion exploring how men perceive weight and dieting in comparison with women. It was noted that some participants felt that dieting was a common approach for women and often discussed by them, particularly around weight and aesthetics, amplified by social media pressure. The negativity expressed by the men inferred that they are less inclined to be drawn into the dieting culture.

- ‘Women it’s **part of that conversation** because now it’s **social media pressure**. And it’s there in the magazines and stuff, whereas it’s **not the topic of conversation primarily for men**. And I think it’s that whole culture change.’
- ‘When we go down to the pub or socialise, we’re **not necessarily talking** about our weight or our appearance as such.’
- ‘I believe that women to **look at dieting a bit differently from men** and potentially different reasons.’
- ‘I feel **women pay more attention to their body**, the size of their body, the shape of their body compared to men.’
- ‘When you look at articles if you go online and they used to have slimming stuff on TV would always have women so there was always an ad there that **women need to look slim**.**’**

ST4 The weight gain journey

There was also reference to the men’s relationships with food, particularly the cyclic nature of dieting. Men outlined a pervasive draw towards prior dietary habits. One participant, who mentions as to how the ‘root cause’ requires tackling, provides some clarity as to the superficial nature of dieting (as well as reaffirming that diets are a temporary approach), which in turn leaves the dieter susceptible to relapse.

- ‘I’d lost my weight and then I though I'll have a doughnut today. And then one doughnut became two... and before you know where you are, **you’re back at the other end** of where you want to be.’
- ‘The problem is me; the problem is the background. **My relationship to food**, the way I'm eating when I'm not dieting.’
- ‘So, you can do that for a certain amount of time, but eventually, something will trigger it, or it will creep back and you will end up on the same kind of weight I've always been.’

- So, if I lose weight and become not big. I then go, right my diet over I'll return to my normal way of eating. But my normal way of eating made me big. So, I'm going to be big again aren't I. So, you really have to tackle the culture, the root cause and not the symptoms.'

Theme 2: Barriers to achieving dietary goals.

When asked to the barriers men experienced as being fundamental to dietary failure, it was outlined that information (or rather misinformation) led to confusion which in turn may obscure key, official dietary recommendations. We further uncover as to how a busy working schedule can disrupt commitment to a dietary strategy.

ST1 Lack of knowledge

- 'Knowledge is the biggest one, because there is **so much misinformation** out there about what makes a successful diet.'

- 'I think knowledge is the key here. Knowing processed food is especially bad for you.'

- 'I did MyFitnessPal …. and that was an absolute pain ... for me because I've got no idea about what calories are or anything. I mean anything at all, and I completely rely on [my wife] so what she used to do was cook the dinner, tell me what was in it and then half the time as I'm trying to find everything and put it into my phone and go.'

The last comment, aside from outlining a lack of knowledge with calories, suggested a dependency on the spouse to both prepare food and support the participant's dieting attempts. Spousal support is further outlined in Theme 4, ST3.

ST2 Lifestyle barriers

Following on from the prior discussion, we uncover how lifestyle factors, in particular working patterns, can be disruptive and could eventually undermine adherence to a dietary regime.

- ‘Other **complications of life** come into that, such as what my wife eats.’
- So, I’m a nurse by trade and so I use to do long 12-hour shifts where you would struggle to be able to eat on the shift regardless of what you were trying to eat. And then at home, you’re exhausted afterwards. **You don’t want to cook particularly good things.** I’ve never found salads and fruit items particularly filling. There might be a psychological factor with that. But I would try and eat something healthy like baked potatoes, beans and a huge salad with it. Build up on the good bits with it. **Half an hour afterwards I’d be sitting there going I’m starving**.’
- ‘That's partly what my lifestyle issue is I’m out coaching tonight and I'm not going to get back until half eight and then we're going to eat then.’
- ‘You come home from work when you sit down because I've been working for eight or nine hours doing a physical job ... and I don't want to go out and do stuff, so you want to eat the simplest things which is kind of pasta or whatever **without realising the effect**.’

Theme 3: Point-of-realisation and motivators for change

All the men had a history of weight gain, and therefore FGI questioning further explored men’s perceptions and feelings regarding body and self-image, as well as how they felt they were perceived by others. This theme also explores participant experiences and the point when they felt the need to act to address their increasing weight. Although there were overlapping sub-themes, they were distinguishable in that ST1 referred to the level of dissatisfaction with oneself and the point of motivation to change, whilst ST2 related to self and external perceptions of image deemed important enough or a ‘threshold’ to trigger action.

ST1 Perception of body/self-image

There appeared to be a point where realisation or self-awareness acted as a key component for personal change. Early on it was made clear to the researchers that the men regularly observed themselves and were overwhelmingly shameful in nature, outlining a negative aesthetic perception of

themselves, which in turn impacted self-confidence. The majority of the men were clearly able to recall, often in detail, these negative feelings prior to attempting weight loss.

- ‘Looking in the mirror and just feeling **absolutely disgusted with myself**.’
- ‘It's **an embarrassment** because I’m thinking, people are looking at me thinking I'm fat again **I can't believe I'm the size I am**.’
- ‘I walked past a mirror and thought, you’re a fat bastard to be perfectly blunt. A mirror, no T-shirt on and I just thought, Jesus that’s **the biggest I’ve ever been.**’
- ‘I actually do like to wear suits and the suits I wear now it’s **not flattering anymore**.’

ST2 Motivation for change

Following open discussion, participant responses focussed more towards the triggers to action and uncovered two core areas: aesthetic appeal and personal identity. Although different perspective, we can see the strong relationships with the previous theme, one referring to the level of dissatisfaction with oneself and the point of motivation to change, whilst the following theme relates to self and external perceptions of image. Responses were overwhelmingly related to negative body/image, and it was made apparent that these perceptions, induced a point in which ‘enough was enough’ and culminated in positive action, albeit often impulsive or sudden.

- ‘It was an **identity** thing; I didn’t want to be seen like this.’
- ‘So, there's a kind of **mismatch** between your identity and who you think you are and who you actually are.’
- So for me personally it wasn't about weight, I never look at weight it’s how aesthetically you look that's what I go for, which is why this is massive for me.
- ‘I **hated photos** of myself.’
- ‘So, for me personally it wasn't about weight, I never look at weight **it’s how aesthetically you look** that's what I go on which is why this is massive for me... it's not about weight I could weigh 100 kilos, 80 kilos it’s **how I think I should aesthetically look**.’
- ‘I think for me if I’m completely honest **I needed the shame**, if that makes sense.’

The second largest motivator to change related to health and usually triggered meaningful action, due a prior health scare or concerns associated with health risk.

- ‘I could feel **something was not right**, and that triggered me to start my exercises.’
- ‘I had **no awareness** of how things were (in relation to a routine health check-up).’
- ‘At home I happened to read a few cases of heart attack of people who are the same age as me and then **I really started thinking about it**, I still don't know whether that was a fear of heart attack or something that triggered me.’
- ‘And then when they said I was diabetic type 2 or whatever it was. They wanted to put me on medication, and **I said no**.’
- ‘I had to have emergency open heart surgery. That was a **big wake up call**. But even more so, the **photo was the more, bigger prompt**.’

Theme 4: Key factors for sustainable dietary control

Findings from the focus group discussion and thematic analysis uncovered potential approaches men viewed as being pivotal for continued engagement through to fruition of a positive dietary outcome. From the FGI’s it was interesting to note that very few of the men reported an approach to weight loss in the absence of peer support, particularly social engagement. Hence the concept of support structures dominated discussion, highlighting potential need for social competitiveness along with peer or partner support.

The comments were expressed as being significant enough to impact engagement with overall, meaningful lifestyle change (often attempted through dietary intervention) and was mainly attributed to their inability to exercise (and recognised as being the fundamental strategy to weight loss) which in its absence, attempts to adopt positive lifestyle change were likely futile e.g., ‘Because of my disabilities I can’t do much in the way of exercise.’ and ‘I can’t put the miles in I used to.’. Picking up on the preference for exercise, further questioning revolved around the methods that could be employed in order engage the men with positive long-term lifestyle change. Here we uncovered the importance placed upon support structures, which in turn dominated discussion, with vehicles for

continued motivation (and subsequent engagement) being competitive, supportive and guided in nature (ST1-ST3). Subtheme 4 culminates in the men's views as to the additional aspects that they felt are pivotal for dietary success.

ST1 'The buddy'

The most popular of these support structures was the 'buddying support system'. This suggestion was commonly acknowledged as being an important approach to long-term engagement to change for men specifically. The idea of 'competitive socialisation' was proposed as being central to sustained engagement in weight management programmes and focussed on male-centric social structures. This level of social accountability coupled with a sport or competitive element was highly noted across the FGIs.

- 'Having a **check in buddy**, somebody on the diet who is motivated enough and says "Okay, do you want to check in buddy, do you want **somebody you could talk to**?'
- 'I think having an **accountability partner** is key to weight loss' – Partnering-up.'
- 'Surround yourself with **people who are on the same mission**.'
- 'That would be certainly much easier if you were collaborating or even **competing with someone,** I think that would be an easier thing to do.'
- 'But if I knew I was going to meet a bunch of people and run around the park together I'd probably **commit more to that**.'

ST2 'The mentor'

Following on from the above sub-theme, in contrast to the need for social companionship (or a 'buddy') some interviewees suggested that direct mentorship would likely enhance accountability and therefore success. Depending upon the individual, some men preferred the need for a knowledgeable 'mentor' to check on progress, provide advice and motivate; accordingly, differing slightly in context to a 'buddy' system by re-positioning themselves into a subordinated role.

- ‘Because **it's always nice for someone to check in on you** and make sure you are sticking to what you say you are going to stick to.’
- ‘If you have a coach, if you hired a coach and contact them regularly and **they motivate you**, give you things to do and you are always checking in with them **so some sort of mentor**.’

ST3 ‘The spouse’

The requirement for male-specific tailored support strategies, whether from a knowledgeable mentor or peer was a common suggestion and yet interestingly, most men also outlined how spousal support was pivotal for sustained success and adaptation to lifestyle change. The men were vocal when expressing how favourable spousal support had been and would often expand to the point of suggesting that the diet was largely orchestrated by the partner which inadvertently suggested that the male, again, acted in a subordinated role by having the spouse prepare meals and provide advice. Furthermore, the comments also suggested that the men may not have fully immersed themselves within the dietary mindset (or did not feel a need to) and instead capitalised on the availability of spousal support to relinquish almost full control, including behavioural aspects, such as goal setting, self-motivation and self-control.

- ‘**My wife structures our meal plan**, but she does like 10 days shopping in a row. And she'll say to me, what do you want to eat on these days? And normally after I’ve just eaten, I don't care, you know, I could have just eaten. But yeah, so we'll figure out what would the menu for the week. We’ll ask the kids what they fancy, and then she'll go out and she'll buy it.’
- ‘The snacks **she buys me** are apples rather than chocolates and stuff like that. That's half the battle.’
- ‘**My wife helps me now a hell of a lot** because she kicks me in the arse when I start to go for the doughnuts again.’
- ‘**For me, it was having the support of my wife**. Without that, I wouldn't have done it because she buys the food, she does the cooking in the evenings because I'm not home till late. ‘If it wasn't for her doing that then **I wouldn't have got as far as I did**.’

ST4 Additional supportive factors

Whist recognising the importance the men placed on peer support, this was not disregarding other factors deemed important for dietary success. However, rather than having several comments supporting a single sub-theme as outlined earlier, the views for additional supportive factors, varied. Knowledge was a recognised as primary skill when embarking and engaging with a diet but also outlined the importance of social interaction, despite being initially dismissed by the recipient.

- ‘Whilst I didn't get much from the group aspect, one of the things I did appreciate from going to the groups was the knowledge I got. Even people that were doing their diet just like I was learning new things. And they were saying by the way, if you make your own sauces, it's really easy to do. You just throw this, this and this in. Oh, that's a good idea. You would get a little bit of knowledge, and you'd get that from like this little messenger group.’

Additional comments outline the array of opinions as to what is important when embarking on and sustaining a diet.

- ‘**Motivation**. It’s all psychological as well, knowing that I've got to weigh. I don't know, there’s something about it. It's acknowledgement that you’ve got to weigh in.’
- ‘**Realistic achievable goals.** In 2019 I was 14 and a half stone in January 2014. Then I got married 2020 January the second I was 10 and a half, so I've lost four stone. And for my frame and my height, I think 10 and a half stone wasn't really realistic. But I managed to maintain that until COVID hit and had to stop going but I don't think my target weight which I hit was realistic.’
- ‘It's knowing it's a long journey and knowing that lesson I learned. You're only tackling the symptoms; you need to **tackle the root cause**.’
- ‘**It needs to be simple**; it can't be something that you have to think about and takes up too much time because I wouldn't bother, I would go, “Oh yes this is fun” and three days later it's no longer fun it's a drag.’

Discussion

The primary aim of this study was to understand lived experiences and male perceptions of weight loss initiatives, including support mechanisms when targeting weight loss. This may support future directions that enhance long-term weight loss sustainability specific to this demographic which is often under-represented [22-24].

One of the key findings from this study was that although the men recognised the positive associations between weight gain and health risk, and the need to manage their weight, the concept of diet and the primary method of weight control (dieting), was viewed negatively by the majority. Dieting conjured up feelings of restraint and outlined restrictions on prior freedoms which in turn eroded willingness to engage. Perception of diet alone was firmly placed within the female domain, and yet for the men of this research, exercise was deemed to be the primary method for weight control, despite multi-component approaches highlighted as the most effective and preferred approach to weight loss (25).

Limiting factors contributing to the unsuccessful pursuit of dieting were primarily centred around the men's own experiences of diets. It was often cited by the men that they had little inclination to persist with a diet, with factors such as temptation, disinclination etc., within a few weeks of conception mentioned as the cause(s) for disengagement. Furthermore, comments outlined an inability to accommodate dietary approaches into their lifestyle, with working patterns having a significant negative impact on dietary efficacy which was compounded by insufficient cooking and food knowledge and skill, leading to a sense of frustration as to both what to cook and the effort to create something that conforms with their dietary requirements

This uncovered feelings of a general dislike of dieting, reinforced by prior experiences that induced barriers for future engagement. Furthermore, short term engagement, provided little time for acclimatisation with dietary principles and integration into daily lifestyle patterns. Lastly, several motivational components suggested as vital for meeting dietary outcomes, including the generation of self-efficacy, realistic goals, internalisation of health messages etc (22,26) appear to be significantly

undermined if expectations and chances of meeting dietary outcomes are eroded from the onset. These negative experiences potentially present limited opportunities to engage men with weight loss in the future.

The observed negative association with diets and weight loss programmes was further echoed in terms of the notion of perceived expected failure. Responses within the FGIs noted a sense of 'generalisation' in dietary approaches to weight management programmes which appeared to be largely directed towards or by feminine connotations associated with aesthetic appeal. This generalised perception of female-centric patterns associated with diet/weight programmes can potentially overlook the importance of masculinised preference (23, 24, 27). The sense of 'restriction' combined with healthy eating was also perceived in a negative light in terms of associated short-term hunger and boredom. The findings from the focus group therefore indicate that weight management programmes directed towards men should paradoxically focus away from 'diet' aspects and are more likely to be successful if driven from a male-personalised narrative. This perspective aligns with previous interventions specific to male-gendered weight management programmes reported elsewhere (16, 28-30).

Leading on from this, barriers to engagement were typically linked to men's own experiences of diets and the unsuccessful pursuit of dieting. Whilst pertaining to diets as being an ineffective strategy, several participants, with pride were happy to express to their peers as to the number of diets they had attempted. The researchers coined the term 'serial dieters' to describe these men and the researchers attributed these announcements to a general competitiveness amongst the men, that as a motivator could, if leveraged correctly, serve to counteract common dietary practises that undermine engagement.

Although some individuals did recognise both the importance of knowledge and the large amount of conflicting narrative, there was no evidence to suggest they had made attempts to explore further for clarity and to develop their knowledge to avoid barriers to uptake and engagement. In addition, often the opinions expressed by the men, suggested that they come from a position of experience and yet, further investigation outlined a lack of understanding around dieting such as the multiple approaches to weight loss, in particular nutrition. The researcher's perceptions were that these

experiences may have largely been pre-conceived ideas developed through for instance social interaction, which in turn acted as deterrents to engagement.

The men perceived women as immersed within the cultural realm of dieting. A consistent narrative associated with stereotypical gender was evident throughout all conducted focus groups. Despite previously being discussed as a facilitator to engagement, it was evident that some males felt the presence of females and female centred strategies would inhibit the opportunity for engagement and could be attributed to the perception that for men, dieting is deemed to be a feminised space where they feel self-conscious and uncomfortable. And yet, often the male participants shared how favourable female spousal support had been; to the point of suggesting that the diet was largely orchestrated by the female partner with the male acting in a participatory role and directed throughout the dietary project. This aligns with research (31-33) and may stem from traditional or stereotypical marital roles, where the female is often responsible for buying and preparing food (34). The researchers were led to a perception, that men felt their female spouse, being immersed within the dieting realm and dieting (alone) were more knowledgeable and experienced within this realm and as such, were able to relinquish control accordingly.

Despite a history of largely negative perceptions surrounding personal weight gain and viewing one's body daily, the impetus to attempt dieting would often stem from a single, significant moment in time. These instances, driven by appearance rather than health (16), were consistently recalled with emotive language and perceived by the researchers to include underlying negative, embarrassing or shameful feelings that impacted self-confidence and promoted sub-standard aesthetic perceptions of themselves when amongst others. Despite external factors influencing the awareness and attitude of the members of the focus groups, there was limited record of responses relating to health. Consistent across internal and external perceptions, male aesthetic improvement was, on the surface, demonstrated to be a more significant measure of success than health or health parameters. In fact, potential implications to health were only discussed as internal motivators by three men, with the emphasis often related to exercise and energy levels, as opposed to diet alone.

Similarly, to aesthetic perception being utilised to motivate engagement with change, an improvement in health was only considered once a ‘scare’ had been encountered. Whether it was aesthetics or a health related ‘scare’ that fuelled a change, the loss of identity or comparison to a former version of themselves often lead the men to a threshold whereby ‘enough was enough’. Prior to this point, a general lethargy was outlined amongst the men, with a tolerated, underlying disquiet with the decline in health and / or aesthetics which was insufficient to induce change. Subsequent action could be directly linked to an epiphany, however notably this change was often sudden, drastic, and thus unsustainable, ultimately resulting in little to no long-term effectiveness.

Vehicles for engagement were occasionally presented as positive and often included competitive behaviours that incentivised action and therefore transpired into relative success with dieting. Demonstrating weight loss improvements via incremental change for example a weight reduction from the previous measurement was shown to be a good motivator for continuation, and in fact the simplicity of competition, whether it was with oneself or as a wider group, became a consistent theme throughout the FGI’s. Simplicity was a concept that was picked up in multiple contexts within the FGI’s, however this principle was always accompanied with competitive undertones, often referring to ‘incentives’ and ‘challenges’ and therefore perhaps indirectly could be associated with motivation to engage in a weight loss programme. Moreover, it was challenging to uncover the men’s feelings surrounding goal failure or a period of disengagement with research suggesting that common reasons for drop-out of weight management programmes for men, centre around a lack of autonomy and support (35). Accountability was a common factor discussed by the men throughout all FGI’s, relating to a preference for a support structure. A highly competitive approach was cited as being useful for engagement, especially through ‘buddying’ where training is undertaken together to reach an agreed goal. This initially appeared to conflict with research suggesting a masculinised requirement for autonomy but further reflection by the researchers suggested that autonomy by the men was perceived as maintaining control rather than a full relinquishment of said control that would be expected within a structured commercial programme. Interestingly, one component of the ‘commercial programme’ that the FGI’s were united in was the notion of either an informed professional, or a previous participant in

the weight loss programme, who could provide mentorship and support when called upon. Further research exploring the forms of spousal dietary support provided to men, that could improve sustainability may provide a rationale for dietary programmes aimed at couples. Regardless of the support participants favoured, the mention of a 'spouse' or significant other, when describing an attempt at a weight loss programme was consistent and agreed upon by many men in the FGI's.

Limitations of the research

There are several limitations within the current study that are important considerations for contextualisation. Whilst the FGIs provided novel insight into male perceptions and lived experiences of dieting, there was relatively limited diversity (mainly married, educated, white men), and therefore future research should consider perspectives representative of other demographics for more holistic understanding. Furthermore, the researchers did not delve into the specific constructs of digital applications or male-centric weight management programs, including use of personalised technology and behavioural coaching initiatives. Additionally, whilst the concept of dieting was discussed, we did not pursue aspects within the diet such as dietary types or diet quality. Future research should therefore consider such aspects with a view to personalising programs specific to male-centered objectives. It was also challenging to uncover the men's feelings surrounding goal failure or a period of disengagement and this was understandable given the requirement to outline, among other men their inability to achieve a particular outcome. Individual interviews may have been more successful in uncovering the men's feelings.

Translational applications

1. Specific to men, diets and dieting are typically perceived in a negative way. Therefore, any strategy purposefully targeting male-centric weight management should focus on male narratives including social competitiveness and tailored aesthetic gains, ahead of health-specific requirements.

2. The relative importance of weight loss is perceived as less important in the men interviewed, inferring that for a weight management programme to be successful, strategies could employ a more 'covert' lifestyle approach (again highlighting social or individual competitiveness) whilst focusing on health-related risk to further introduce a proactive approach.
3. Barriers to change pertinent to male narratives need to be considered as part of a weight management strategy, with 'opportunistic simplicity' potentially being key.
4. A noted 'point of realisation' could be brought to the individual's attention earlier in the behavioural change cycle, which could lead to earlier incentivisation to induce effective engagement with a programme.
5. Strategies for longer-term male success should focus on simplicity coupled with quick-wins, early rewards and a tailored degree of social competitiveness.
6. A novel aspect of this study was the identification of the importance of tailored interplay between buddy-mentor-spousal support as fundamental to early and sustained success, with the emphasis likely towards spousal support.

Conclusions

This research proved instrumental in understanding male perceptions around the realm of dieting. Drivers for change stemmed primarily from aesthetics although a health 'scare' would help drive a person to adapt meaningful lifestyle changes. Consensus outlined a general uneasiness with prescribed dietary approaches which suggested a distancing from the female realm of dieting. However, discourse also suggested the men persistently engaged with diets to induce weight loss (albeit superficial in nature). A primary factor identified for longer-term dietary success was peer support with three primary relationships outlined: mentor, buddying and spouse. Recruitment of spousal support was uncovered as likely the most effective method for supporting longer term engagement and goal-achievement, facilitated through the relegation of control to the spouse. Findings from this study highlight that sustainable lifestyle change specific to male populations need to be gender tailored and not generalised.

Acknowledgements

The authors wish to thank the participants for their time and commitment in supporting this research.

References

1. Lowe DA, Wu N, Rohdin-Bibby L, Moore AH, Kelly N, Liu YE, et al. Effects of Time-Restricted Eating on Weight Loss and Other Metabolic Parameters in Women and Men With Overweight and Obesity: The TREAT Randomized Clinical Trial. JAMA Intern Med. 2020 Nov 1;180(11):1491-1499. doi: 10.1001/jamainternmed.2020.4153.
2. Soliman GA. Intermittent fasting and time-restricted eating role in dietary interventions and precision nutrition. Front Public Health. 2022 Oct 28;10:1017254. doi: 10.3389/fpubh.2022.1017254.
3. Malhotra V, Sawal A. Metabolic Effects of Ketogenic Diets and Their Utilization in Obesity Management: A Systematic Review. Cureus. 2023 Mar 26;15(3):e36720. doi: 10.7759/cureus.36720.
4. Choi YJ, Jeon SM, Shin S. Impact of a Ketogenic Diet on Metabolic Parameters in Patients with Obesity or Overweight and with or without Type 2 Diabetes: A Meta-Analysis of Randomized Controlled Trials. Nutrients. 2020 Jul 6;12(7):2005. doi: 10.3390/nu12072005.
5. Wewege M, van den Berg R, Ward RE, Keech A. The effects of high-intensity interval training vs. moderate-intensity continuous training on body composition in overweight and obese adults: a systematic review and meta-analysis. Obes Rev. 2017 Jun;18(6):635-646. doi: 10.1111/obr.12532.
6. Dent R, McPherson R, Harper ME. Factors affecting weight loss variability in obesity. Metabolism. 2020 Dec;113:154388. doi: 10.1016/j.metabol.2020.154388.

7. NICE. Obesity prevention [Internet]. National Institute for Health and Care Excellence; 2006. Report No.: CG43. Available from: https://www.nice.org.uk/guidance/cg43.
8. National Institute for Health and Care Excellence. Obesity: identification, assessment and management. 2022 Sep. Report No.: CG189.
9. Anderson JW, Konz EC, Frederich RC, Wood CL. Long-term weight-loss maintenance: a meta-analysis of US studies. Am J Clin Nutr. 2001 Nov;74(5):579-84. doi: 10.1093/ajcn/74.5.579.
10. Thomas JG, Bond DS, Phelan S, Hill JO, Wing RR. Weight-loss maintenance for 10 years in the National Weight Control Registry. Am J Prev Med. 2014 Jan;46(1):17-23. doi: 10.1016/j.amepre.2013.08.019.
11. Phelan S, Halfman T, Pinto AM, Foster GD. Behavioral and Psychological Strategies of Long-Term Weight Loss Maintainers in a Widely Available Weight Management Program. Obesity (Silver Spring). 2020 Feb;28(2):421-428. doi: 10.1002/oby.22685.
12. Spreckley M, Seidell J, Halberstadt J. Perspectives into the experience of successful, substantial long-term weight-loss maintenance: a systematic review. Int J Qual Stud Health Well-being. 2021 Dec;16(1):1862481. doi: 10.1080/17482631.2020.1862481.
13. Hartmann-Boyce J, Boylan AM, Jebb SA, Aveyard P. Experiences of Self-Monitoring in Self-Directed Weight Loss and Weight Loss Maintenance: Systematic Review of Qualitative Studies. Qual Health Res. 2019 Jan;29(1):124-134. doi: 10.1177/1049732318784815.
14. Greaves C, Poltawski L, Garside R, Briscoe S. Understanding the challenge of weight loss maintenance: a systematic review and synthesis of qualitative research on weight loss maintenance. Health Psychol Rev. 2017 Jun;11(2):145-163. doi: 10.1080/17437199.2017.1299583.
15. Patton, M. 2002. Qualitative research and evaluation methods. 3rd Edition. Sage Publications; Thousand Oaks, CA.
16. Cortnage M, Pringle A. Onset of Weight Gain and Health Concerns for Men: Findings from the TAP Programme. Int J Environ Res Public Health. 2022 Jan 5;19(1):579. doi: 10.3390/ijerph19010579.

17. Donnachie C, Sweeting H, Hunt K. A Qualitative Study on Young Men's Experiences of Intentional Weight-Gain. Int J Environ Res Public Health. 2023 Feb 14;20(4):3320. doi: 10.3390/ijerph20043320.

18. Lin YC, Liu PC, Chou FF, Hou IC, Chou CC, Chen CW, et al. Beliefs underlying weight control behaviors among adolescents and emerging adults living with obesity: an elicitation qualitative study. BMC Public Health. 2024 Nov 27;24(1):3292. doi: 10.1186/s12889-024-20677-5.

19. Guest G, Namey E, Taylor J, Eley N, and McKenna K. Comparing focus groups and individual interviews: findings from a randomized study. Int J Social Res Methodol. 2017; 20(6), 693–708. https://doi.org/10.1080/13645579.2017.1281601.

20. Braun V, and Clarke V. Using thematic analysis in psychology. Qual Res Psychol. 2006; 3(2): 77–101. doi: 10.1191/1478088706qp063oa.

21. Vaismoradi M, Turunen H, Bondas T. Content analysis and thematic analysis: Implications for conducting a qualitative descriptive study. Nurs Health Sci. 2013 Sep;15(3):398-405. doi: 10.1111/nhs.12048.

22. Skelly LJ, Smyth PP, Donnelly MP, Leslie JC, Leader G, Simpson L, et al. Factors that potentially influence successful weight loss for adults with intellectual disabilities: A qualitative comparison. J Intellect Disabil. 2021 Dec;25(4):458-475. doi: 10.1177/1744629520931681.

23. Tsai SA, Lv N, Xiao L, Ma J. Gender Differences in Weight-Related Attitudes and Behaviors Among Overweight and Obese Adults in the United States. Am J Mens Health. 2016 Sep;10(5):389-98. doi: 10.1177/1557988314567223.

24. Morgan PJ, Collins CE, Plotnikoff RC, McElduff P, Burrows T, Warren JM, et al. The SHED-IT community trial study protocol: a randomised controlled trial of weight loss programs for overweight and obese men. BMC Public Health. 2010 Nov 16;10:701. doi: 10.1186/1471-2458-10-701.

25. National Institute for Health and Care Excellence. Overweight and obesity management NICE guideline [NG246] [Internet]. NICE; 2025 Jan. Available from: https://www.nice.org.uk/guidance/ng246/evidence.
26. Dicker D, Alfadda AA, Coutinho W, Cuevas A, Halford JCG, Hughes CA, et al. Patient motivation to lose weight: Importance of healthcare professional support, goals and self-efficacy. Eur J Intern Med. 2021 Sep;91:10-16. doi: 10.1016/j.ejim.2021.01.019.
27. Levi A, Chan KK, Pence D. Real men do not read labels: the effects of masculinity and involvement on college students' food decisions. J Am Coll Health. 2006 Sep-Oct;55(2):91-8. doi: 10.3200/JACH.55.2.91-98.
28. Hunt K, Wyke S, Gray CM, Anderson AS, Brady A, Bunn C, et al. A gender-sensitised weight loss and healthy living programme for overweight and obese men delivered by Scottish Premier League football clubs (FFIT): a pragmatic randomised controlled trial. Lancet. 2014 Apr 5;383(9924):1211-21. doi: 10.1016/S0140-6736(13)62420-4.
29. Pringle A, Zwolinsky S, McKenna J, Robertson S, Daly-Smith A, White A. Health improvement for men and hard-to-engage-men delivered in English Premier League football clubs. Health Educ Res. 2014 Jun;29(3):503-20. doi: 10.1093/her/cyu009.
30. Robertson C, Avenell A, Boachie C, Stewart F, Archibald D, Douglas F, et al. Should weight loss and maintenance programmes be designed differently for men? A systematic review of long-term randomised controlled trials presenting data for men and women: The ROMEO project. Obes Res Clin Pract. 2016 Jan-Feb;10(1):70-84. doi: 10.1016/j.orcp.2015.04.005.
31. Albanese AM, Huffman JC, Celano CM, Malloy LM, Wexler DJ, Freedman ME, et al. The role of spousal support for dietary adherence among type 2 diabetes patients: a narrative review. Soc Work Health Care. 2019 Mar;58(3):304-323. doi: 10.1080/00981389.2018.1563846.
32. Beverly EA, Wray LA. The role of collective efficacy in exercise adherence: a qualitative study of spousal support and type 2 diabetes management. Health Educ Res. 2010 Apr;25(2):211-23. doi: 10.1093/her/cyn032.

33. Tripathee S, Sweeting H, Chambers S, Maclean A. How men receive and utilise partner support when trying to change their diet and physical activity within a men's weight management programme. BMC Public Health. 2020 Feb 7;20(1):199. doi: 10.1186/s12889-020-8213-z.
34. Wong M, Gucciardi E, Li L, Grace SL. Gender and nutrition management in type 2 diabetes. Can J Diet Pract Res. 2005 Winter;66(4):215-20. doi: 10.3148/66.4.2005.215.31.
35. Archibald D, Douglas F, Hoddinott P, van Teijlingen E, Stewart F, Robertson C, Boyers D, Avenell A. A qualitative evidence synthesis on the management of male obesity. BMJ Open. 2015 Oct 12;5(10):e008372. doi: 10.1136/bmjopen-2015-008372.

Supporting information

S1 File. Appendix showing general framework for focus group questions.

Ethics approval letter

Click here to access/download
Supporting Information
Ethics_application_ETH2122-0617_Decision.pdf

Click here to access/download
Supporting Information
Supplementary Material Revised.docx

Male perceptions and experiences of weight loss initiatives: recommendations towards sustainable lifestyle practice.

Mark Cortnage[1¶*], Joseph Lillis[2¶], Justin Roberts[2¶*]

[1] Faculty of Health, Medicine and Social Care, School of Allied Health and Social Care, Anglia Ruskin University, Cambridge, CB1 2LZ, UK

[2] ~~Cambridge Centre for Sport and Exercise Sciences,~~ Faculty of Science and Engineering, School of Psychology, Sport and Sensory Sciences, Anglia Ruskin University, Cambridge, CB1 1PT, UK

[*] Corresponding authors~~ence~~

Email: ~~:~~ mark.cortnage@aru.ac.uk (MC); justin.roberts@aru.ac.uk (JR)

[¶] These authors contributed equally to this work

Abstract~~:~~

This study aimed to collect lived experiences and male perceptions of weight loss initiatives, as well as understand the support mechanisms used by men when targeting weight loss, with a view to informing sustainable lifestyle practices. ~~Methods:~~ Following informed consent, a total of 24 participants (age: 46±10 yrs.; body mass: 91.1±13.2 kg; height: 1.79±0.06 m; BMI: 28.5±4.4 kg·m^2) took part in four focus group interviews (FGIs). All interviews were undertaken by experienced male researchers using a semi-structured approach following the same set of pre-determined open-ended questions, with additional lines of enquiry included as appropriate. Interviews were recorded with MP3 files transcribed for later thematic analysis and coding. Thematic analysis highlighted four main themes including: 1) male-specific perceptions of dieting and weight loss; 2) barriers to achieving dietary goals; 3) point-of-realisation and motivators for change; and 4) key factors for sustainable dietary control. Overall, the men perceived diet/dieting in a negative light, acknowledging the female-centric nature of

Formatted: Font: (Default) Times New Roman
Formatted: Line spacing: Double
Formatted: Font: (Default) Times New Roman, Superscript
Formatted: Font: (Default) Times New Roman
Formatted: Font: (Default) Times New Roman, Superscript
Formatted: Font: (Default) Times New Roman
Formatted: Font: (Default) Times New Roman, Superscript
Formatted: Font: (Default) Times New Roman
Formatted: Font: (Default) Times New Roman
Formatted: Font: (Default) Times New Roman, Superscript
Formatted: Font: (Default) Times New Roman
Formatted: Font: (Default) Times New Roman, Superscript
Formatted: Font: (Default) Times New Roman
Formatted: Font: (Default) Times New Roman, Superscript
Formatted: Font: (Default) Times New Roman
Formatted: Font: (Default) Times New Roman
Formatted: Font: (Default) Times New Roman
Formatted: Font: (Default) Times New Roman
Formatted: Font: (Default) Times New Roman
Formatted: Font: (Default) Times New Roman, Not Superscript/ Subscript
Formatted: Font: (Default) Times New Roman
Formatted: Font: (Default) Times New Roman, 18 pt, Bold
Formatted: Font: (Default) Times New Roman, Bold
Formatted: Font: (Default) Times New Roman

weight loss programs, and the need for male-specific narratives as well as recognising the cyclic nature of dieting. This coupled with barriers of low motivation (and expectations of failure), lifestyle demands and misinformation (particularly around diets). Drivers for change were often focused on aesthetics over health factors and outlined an impulsiveness to diet. Three specific support systems were highlighted as potentially effective strategies to elicit sustainable engagement with dietary management.

~~**Keywords: weight loss; perceptions; experiences; lifestyle; male; sustainability, diet, aesthetics.**~~

~~Background~~Introduction

Overweight and obesity are current primary health concerns within the UK population [1]. This is particularly relevant in adult males, with the overall proportion designated as overweight or living with obesity being 67% in 2022 [1]. Within specific age categories, prevalence of overweight/obesity has been reported greater for men aged 35-44 years (75%), 45-54 years (73%) and 55-64 years (80%) respectively [1]. This highlights the importance of both tailored and sustainable weight management solutions particularly for those approaching mid- to older ages.

Weight loss incentives often comprise of singular approaches including dietary restriction and physical activity. At the time of writing, there is significant focus on time-restricted dietary patterns (1,2), keto-genic/high-protein diets (3,4) and vigorous, intermittent exercise (5). In many cases, such programmes provide a short-term mechanistic approach to 'kickstart' metabolic processes pertinent to progressive metabolic flexibility and anthropometrical changes and fail to accommodate the complex interplay between lifestyle and behavioural modifications, cross-linked with energy and hormonal balance, genetics and environmental factors (6). Therefore multi-component approaches to weight loss are preferable for supporting weight loss and sustained management (7,8) with long term significant weight management achievable with specific strategies supporting lifestyle and behavioural change for adults (9,10) particularly with male cohorts. The longer the period from weight loss through to

Formatted: Font: (Default) Times New Roman
Formatted: Indent: First line: 0.5", Line spacing: Double
Formatted: Font: (Default) Times New Roman, Bold
Formatted: Line spacing: Double
Formatted: Font: (Default) Times New Roman, 18 pt
Formatted: Font: 18 pt
Formatted: Font: (Default) Times New Roman, 18 pt
Formatted: Font: (Default) Times New Roman
Formatted: Indent: First line: 0.5", Line spacing: Double
Formatted: Font: (Default) Times New Roman
Formatted: Font: (Default) Times New Roman
Formatted: Font: (Default) Times New Roman
Formatted: Font: (Default) Times New Roman
Formatted: Font: (Default) Times New Roman
Formatted: Font: (Default) Times New Roman
Formatted: Font: (Default) Times New Roman
Formatted: Font: (Default) Times New Roman
Formatted: Font: (Default) Times New Roman
Formatted: Font: (Default) Times New Roman
Formatted: Font: (Default) Times New Roman
Formatted: Font: (Default) Times New Roman

maintenance, the increased likelihood of sustaining weight (10,11) and underlines the need for sustained, prolonged support with goal setting, monitoring and improved self-perception and control as facilitators to meaningful change (12,13).

Several notable systematic reviews have been conducted exploring the experiences of successful weight loss maintainers (13,14). However, the majority of the study participants were women and underline the paucity of data exploring male experiences of weight loss and perceptions of the barriers and facilitators for successful maintenance of weight. Exploration of male opinions around weight loss practices have yet to be conducted specifically around motivations for weight loss, efficacy, experiences and preferred approaches. Therefore, the aim of this study was to collect lived experiences and perceptions of weight loss and maintenance from adult men, to increase our understanding of male gendered perspectives around dieting.

~~Methods~~Materials and methods

Ethical approval and participant recruitment

Following University Ethics Committee approval (ETH2122-0617) participants were recruited through convenience sampling utilising multiple methods employing purposive and snowball sampling approaches between June-July 2022. Recruitment consisted of word of mouth and personal contacts, through to study advertisement promoted locally (institutional intranet and public forum marketing) through to social media (Facebook/LinkedIn) promotion of the study. Additionally, study advertisement was promoted via local government authority health initiatives following gatekeeper approval. From this, 30 male participants volunteered~~eds were invited~~ to take part in one of four ~~a~~ single-case focus groups.

For study inclusion, participants were required to be male, aged > 30 yrs, in general good health, and able to communicate in English. Participants were also required to have a lived experience of being overweight, having previously undertaken a weight-loss programme or were actively aiming to lose weight in the near future, and therefore willing and able to contribute to discussions around perceptions

Formatted: Font: (Default) Times New Roman
Formatted: Font: (Default) Times New Roman
Formatted: Font: (Default) Times New Roman
Formatted: Font: (Default) Times New Roman
Formatted: Font: (Default) Times New Roman
Formatted: Font: (Default) Times New Roman
Formatted: Font: (Default) Times New Roman, 18 pt
Formatted: Line spacing: Double
Formatted: Font: 18 pt
Formatted: Font: (Default) Times New Roman, 18 pt
Formatted: Font: (Default) Times New Roman, 16 pt
Formatted: Font: (Default) Times New Roman
Formatted: Font: (Default) Times New Roman
Formatted: Font: (Default) Times New Roman
Formatted: Font: (Default) Times New Roman
Formatted: Font: (Default) Times New Roman
Formatted: Font: (Default) Times New Roman
Formatted: Indent: First line: 0.5", Line spacing: Double

and experiences of weight loss initiatives within a semi-structured focus group discussion setting. Participants were excluded if they were: < 30 years, had an underlying medical condition that would make participation unsafe or inappropriate, or had no prior engagement with weight loss behaviours. Participants were also excluded if they unwilling to be audio-recorded or unable to provide informed consent.~~Participants were required to be aged >30 yrs, in good general health, and had previously undertaken a weight loss programme or aiming to lose weight in the near future for the purposes of the focus group.~~ From the initial cohort and following study inclusion matching, 24 participants (age: 46±10 yrs.; body mass: 91.1±13.2 kg; height: 1.79±0.06 m; BMI: 28.5±4.4 kg·m^2) took part in the focus group. Based on a criterion-based purposive sampling framework (15), and in line with previous research (16-18), sample size was considered satisfactory.~~Participants were recruited via local advertisement and social media between 06/06-21/07/2022. Recruitment consisted of advertisement within regional leisure groups, Local Authority commissioned programmes, and social media postings.~~

Focus groups

This study employed a qualitative descriptive design using focus groups to explore men's perceptions and experiences of weight loss initiatives. Based on previous work within our group (16) and recognising challenges faced with participant recruitment when exploring potentially sensitive topics, use of a focus group approach was deemed appropriate to provide an informal, yet safe environment for participants to proactively engage in open and honest discussion. Indeed, where there is peer support or discussion amongst participants with similar shared experiences this can provide deeper and richer qualitative information (19). Therefore, f~~F~~ollowing participant recruitment, four smaller focus group interviews (FGIs) were held in which more targeted discussion could be undertaken. Participants were provided with full study information sheets clearly outlining the aim and objectives of the study, as well as general expectations. Participation in the study was voluntary with freedom to withdraw without prejudice. FGIs were held in an informal meeting room at Anglia Ruskin University, Cambridge (in an open planned circle arrangement to foster equality, open communication and active listening throughout) or via Microsoft Teams (for those unable to attend face-to-face FGIs) with all participants providing written, informed consent prior to participation. All interviews were

Formatted: Font: (Default) Times New Roman

Formatted: Font: (Default) Times New Roman

Formatted: Font: (Default) Times New Roman

Formatted: Font: (Default) Times New Roman, 16 pt

Formatted: Line spacing: Double

Formatted: Font: (Default) Times New Roman

Formatted: Font: (Default) Times New Roman

Formatted: Font: (Default) Times New Roman

Formatted: Font: (Default) Times New Roman

undertaken following the same set of pre-determined open-ended questions, with additional lines of enquiry included as appropriate. The interviews therefore followed a semi-structured approach providing flexibility for discussion. The focus group question framework (see ~~Appendix A~~S1 File.) was developed by the researchers based on: i) review of existing literature on male engagement with weight loss programmes, relevant behavioural change strategies, relevant prevalence/ incidence data and gender-specific barriers; ii) theoretical considerations in including hegemonic practices, male help seeking behaviours, reports on lifestyle behaviour change, masculine norms, and both general and specific weight-management challenges/facilitators; and iii) the research teams prior work, which highlighted gaps in understanding men's motivations and experiences with weight loss and management. Following initial team discussion (pertinent to the study objectives and key themes), open-ended questions were developed and refined to encourage more detailed responses and wider group discussion. This resulted in 3 distinct categories in logical order, and 14 prompt questions (with additional probing questions to further explore responses consistent with a semi-structured and flexible approach). The FGIs were conducted by experienced personnel to explore male participant experiences, attitudes, perceptions, and beliefs surrounding weight loss and maintenance, with each one lasting approximately 60 minutes for standardisation. Each interview involved a lead moderator (MC) and objective moderator (JR/JL) to ensure consistency and rigor. All interviews were recorded in English using a TileRec Digital USB 3.0 Type C voice activated recorder, with MP3 files transcribed verbatim via an institution-approved provider for later thematic analysis and coding. Participant characteristics are shown in Table 1. Participants were compensated for their time with a standard gift voucher upon completion of the focus group.

Table 1. Participant characteristics for focus group interviews.

Focus group	Age (years)	Ethnicity	Marital status	Education	Employment
	67	White	M	Degree	FT

Formatted: Font: (Default) Times New Roman

Formatted: Font: (Default) Times New Roman

Formatted: Font: (Default) Times New Roman

Formatted: Font: (Default) Times New Roman

Formatted: Font: (Default) Times New Roman

Formatted: Font: (Default) Times New Roman

Formatted: Font: (Default) Times New Roman

Formatted: Indent: Left: 0.88", Line spacing: Double

Formatted: Line spacing: Double

Formatted Table

Formatted: Font: (Default) Times New Roman

Formatted: Line spacing: Double

	40	White	M	Degree	FT
**Focus Group 1**	40	White	M	Degree	FT
**In Person**	55	White	NM	School	RET
	40	Asian	M	Degree	FT
	31	White	NM	PG	ST
	38	White	NM	Degree	FT
	56	White	M	PG	PT
**Focus Group 2**	30	White	NM	PG	ST
**Online**	43	White	M	Degree	FT
	56	White	M	School	FT
	32	Asian	NM	PG	ST
	41	White	M	Degree	FT
	54	White	M	PG	FT
	42	White	M	Degree	FT
**Focus Group 3**	45	White	NM	School	FT
**Online**	58	White	M	Degree	FT
	36	White	NM	Degree	FT
	44	White	M	PG	FT
	32	White	M	Degree	FT
	54	White	NM	Degree	FT
**Focus Group 4**	48	White	M	PG	FT
**In Person**	56	White	NM	Degree	FT
	60	White	M	PG	PT

~~Table 1. Participant characteristics for focus group interviews.~~

PG PG= Postgraduate; FT = Full-time; RET= Retired; PT = Part-time;
ST = student; M= Married; NM = Not married

Thematic analysis

Formatted: Font: (Default) Times New Roman
Formatted: Line spacing: Double
Formatted: Font: (Default) Times New Roman
Formatted: Line spacing: Double
Formatted: Font: (Default) Times New Roman
Formatted: Font: (Default) Times New Roman
Formatted: Line spacing: Double
Formatted: Font: (Default) Times New Roman
Formatted: Font: (Default) Times New Roman
Formatted: Line spacing: Double
Formatted: Font: (Default) Times New Roman
Formatted: Font: (Default) Times New Roman
Formatted: Justified, Indent: Left: 0.89", Right: 0.76", Line spacing: Double
Formatted: Font: (Default) Times New Roman
Formatted: Font: (Default) Times New Roman
Formatted: Line spacing: Double
Formatted: Font: (Default) Times New Roman, 16 pt

Thematic analysis was employed to provide a structured approach to summarise similarities, differences and key features from the breadth of interview data. Braun and Clarkes six stage model (20~~15~~) was employed to permit appropriate exploration of all necessary realms ([i] transcription/idea generation; [ii] code generation; [iii] theme searching; [iv] theme review/code alignment; [v] theme definition; [vi] theme report) before identifying specific markers of interest, theories and ideas prior to synthesising final conclusions. The first two stages, data familiarisation and the generation of initial codes was directly followed by stage three to five. Coding was initially conducted by two researchers (MC and JL) with second phase confirmation by a third researcher (JR). As such, this triangulation approach was employed to minimise risk of bias. All authors were involved with final confirmation of assigned themes. The identification, reviewing and defining of themes refined the specific detail of each theme and therefore the underlying narrative embedded within the data (~~16~~21). The production of the report, stage six, moved beyond code and theme descriptors presenting an overarching message correlating directly with the primary aim of the study. By the fourth focus group, similar contexts, perspectives and experiences were noted and confirmed during the thematic analysis process inferring that saturation had likely been reached.

~~Thematic Analysis Results~~Results

From the thematic analysis, four core themes and 12 sub themes (ST) were uncovered as follows:

Theme 1: **Male-specific perceptions of dieting and weight loss**, including ST1 (~~Impressions~~ impressions of dieting), ST2 (~~Perceived~~ perceived effort and willpower), ST3 (~~Gendered~~ gendered perspectives), ST4 (~~The~~ the weight gain journey).

Theme 2: **Barriers to achieving dietary goals**, including ST1 (~~Lack~~ lack of knowledge), ST2 (~~Lifestyle~~ lifestyle barriers).

Formatted: Font: (Default) Times New Roman

Formatted: Indent: First line: 0.5", Line spacing: Double

Formatted: Font: (Default) Times New Roman

Formatted: Font: (Default) Times New Roman

Formatted: Font: (Default) Times New Roman

Formatted: Font: (Default) Times New Roman

Formatted: Font: (Default) Times New Roman

Formatted: Font: (Default) Times New Roman

Formatted: Line spacing: Double

Formatted: Font: (Default) Times New Roman, 18 pt

Formatted: Font: 18 pt

Formatted: Font: (Default) Times New Roman, 18 pt

Formatted: Font: (Default) Times New Roman

Formatted: Indent: First line: 0.5", Line spacing: Double

Formatted: Font: (Default) Times New Roman, Not Italic

Formatted: Line spacing: Double

Formatted: Font: (Default) Times New Roman

Formatted: Font: (Default) Times New Roman

Formatted: Font: (Default) Times New Roman

Formatted: Font: (Default) Times New Roman

Formatted: Font: (Default) Times New Roman

Formatted: Font: (Default) Times New Roman, Not Italic

Formatted: Font: (Default) Times New Roman

Formatted: Font: (Default) Times New Roman

Formatted: Font: (Default) Times New Roman

Theme 3: **Point-of-realisation and motivators for change**, including ST1 (~~Perception~~ perception and body/self-image), and ST2 (~~Motivation~~ motivation for change).

Theme 4: **Key factors for sustainable dietary control**, including ST1 ('The buddy'), ST2 ('The mentor'), ST3 ('The spouse') and ST4 (~~Additional~~ additional supportive factors).

Further assessment of the 4 core themes is demonstrated below, along with supporting participant quotations as pertinent.

Theme 1: Male-specific perceptions of dieting and weight loss

ST1 Impressions of dieting

Collated findings from the FGIs outlined that on the whole men perceived dieting, and associated connotations including weight loss, in a generally negative manner associated to the short-term perception of dieting, particularly the restrictive nature of the approach. This essentially implied that strategies to support men should not emphasise or focus on terminology associated with diet/restriction. This was portrayed by participants as follows:

- 'I associate diet with dieting as having **negative connotation**s…my perception is that diets are **not successful**.'
- 'I think diet is a very negative word because as soon as you mention the word diet everybody automatically thinks **restriction, restriction, restriction**.'
- 'I think diets are often a **short-term crash programm**e and it's very difficult to sustain those'
- [Diets involve] '**Denying yourself** certain things.'
- 'When I'm looking at doing any kind of restrictions or dieting **a part of me kind of rebels** against it as it were.'

Despite these negative perceptions, several participants, alluded to (with a degree of self-pride) the number of diets they had attempted. It was therefore noteworthy that whilst diets were perceived as

Formatted: Font: (Default) Times New Roman, Not Italic
Formatted: Font: (Default) Times New Roman
Formatted: Font: (Default) Times New Roman
Formatted: Font: (Default) Times New Roman
Formatted: Font: (Default) Times New Roman, Not Italic
Formatted: Font: (Default) Times New Roman
Formatted: Font: (Default) Times New Roman, 14 pt
Formatted: Font: (Default) Times New Roman
Formatted: Font: (Default) Times New Roman
Formatted: Font: (Default) Times New Roman, 16 pt
Formatted: Font: (Default) Times New Roman, 14 pt, No underline
Formatted: Font: (Default) Times New Roman
Formatted: Indent: First line: 0.25", Line spacing: Double
Formatted: Font: (Default) Times New Roman, Not Italic
Formatted: Line spacing: Double
Formatted: Font: (Default) Times New Roman
Formatted: Indent: First line: 0.25", Line spacing: Double

wholly negative, there was perceived merit in accumulating dietary attempts as a need for personal challenge. Fundamentally this highlighted, to the researchers, the often-poor efficacy associated with more generic, commercial programmes but which is perceived by the participants as a challenge, that if achieved, equates to weight loss (sustainable or not); and if unsuccessful, could act to prove that their negative portrayals of dieting are justified. Furthermore, opinions suggest that diets are cyclic in nature consisting of sustained periods of weight gain intermitted with periods of dieting.

- ‘I’ve done south beach…I tried Lighter Life, that gave me stomach aches, so did SlimFast… I did Slimming world, but I don’t like the Americanised, yeeha meetings…’
- ‘I’ve done the 5:2 intermittent 500 calories in a day isn’t really good, I was thinking about doing the 16:8…I’ve tried vegetarian diet but I gained on that one.’
- ‘**I've pretty much been yo-yo dieting since I'm 18 up** and down up and down, so I've been in that situation many times.’

ST2 Perceived effort and willpower

There was pertinent discussion surrounding the self-discipline and effort required as well as the motivation to engage in such programmes, supporting ST1. With the large number of men acknowledging these statements, it was evident that this was a regular factor impacting programme engagement. Furthermore, the men demonstrated that even the smallest of perceived negative encounters when undertaking a weight loss programme, could act to disproportionately demotivate male participants in future attempts.

- ‘**Temptation** is normally the thing that breaks you.’
- ‘Even the **slightest set back** can bring the whole thing crashing down.’
- ‘Now calorie counting, I’m not going to do it just because I can’t be arsed.’

ST3 Gendered perspectives

It was evident that men identified with a male gendered narrative when it comes to dieting and/or weight loss. A proposition by one participant presented a gendered-binary perspective suggesting that

Formatted: Font: (Default) Times New Roman, Not Italic

Formatted: Line spacing: Double

Formatted: Font: (Default) Times New Roman, 14 pt, No underline

Formatted: Font: (Default) Times New Roman

Formatted: Indent: First line: 0.25", Line spacing: Double

Formatted: Font: (Default) Times New Roman, Not Italic

Formatted: Line spacing: Double

Formatted: Font: (Default) Times New Roman, 14 pt, No underline

Formatted: Font: (Default) Times New Roman

Formatted: Indent: First line: 0.25", Line spacing: Double

women utilise ‘dieting’ which contrasts with male-centric competitive sporting approaches. He did accept that not all men prefer exercise to lose weight and the need for alternative approaches.

- ‘I think a lot of men, if you like sport, because this is okay if you're doing it with people who are active or been active historically…done a lot of sports, I've done a lot of sport but there's an awful lot of blokes out there that don't do sport so you need to try and find another way to **challenge them** to realise yes, it’s not a diet **you’re not a woman just accept the fact** you're a bit overweight and this is the way to do it, a way to do it not the way.’

Clarification as to what the participant meant when suggesting that women diet, led to an open discussion exploring how men perceive weight and dieting in comparison with women. It was noted that some participants felt that dieting was a common approach for women and often discussed by them, particularly around weight and aesthetics, amplified by social media pressure. The negativity expressed by the men inferred that they are less inclined to be drawn into the dieting culture.

- ‘Women it’s **part of that conversation** because now it’s **social media pressure**. And it’s there in the magazines and stuff, whereas it’s **not the topic of conversation primarily for men**. And I think it’s that whole culture change.’
- ‘When we go down to the pub or socialise, we’re **not necessarily talking** about our weight or our appearance as such.’
- ‘I believe that women to **look at dieting a bit differently from men** and potentially different reasons.’
- ‘I feel **women pay more attention to their body**, the size of their body, the shape of their body compared to men.’
- ‘When you look at articles if you go online and they used to have slimming stuff on TV would always have women so there was always an ad there that **women need to look slim.’**

ST4 The weight gain journey

There was also reference to the men’s relationships with food, particularly the cyclic nature of dieting. Men outlined a pervasive draw towards prior dietary habits. One participant, who mentions as

to how the ‘root cause’ requires tackling, provides some clarity as to the superficial nature of dieting (as well as reaffirming that diets are a temporary approach), which in turn leaves the dieter susceptible to relapse.

- ‘I’d lost my weight and then I though I'll have a doughnut today. And then one doughnut became two... and before you know where you are, **you’re back at the other end** of where you want to be.’
- ‘The problem is me; the problem is the background. **My relationship to food**, the way I'm eating when I'm not dieting.’
- ‘So, you can do that for a certain amount of time, but eventually, something will trigger it, or it will creep back and you will end up on the same kind of weight I've always been.’
- So, if I lose weight and become not big. I then go, right my diet over I’ll return to my normal way of eating. But my normal way of eating made me big. So, I'm going to be big again aren’t I. So, you really have to tackle the culture, the root cause and not the symptoms.’

Theme 2: Barriers to achieving dietary goals.

When asked to the barriers men experienced as being fundamental to dietary failure, it was outlined that information (or rather misinformation) led to confusion which in turn may obscure key, official dietary recommendations. We further uncover as to how a busy working schedule can disrupt commitment to a dietary strategy.

ST1 Lack of knowledge

- ‘Knowledge is the biggest one, because there is **so much misinformation** out there about what makes a successful diet.’
- ‘I think knowledge is the key here. Knowing processed food is especially bad for you.’

Formatted: Font: (Default) Times New Roman, Not Italic

Formatted: Line spacing: Double

Formatted: Font: (Default) Times New Roman

Formatted: Font: (Default) Times New Roman, 16 pt

Formatted: Font: (Default) Times New Roman

Formatted: Indent: First line: 0.5", Line spacing: Double

Formatted: Font: (Default) Times New Roman, 14 pt, No underline

Formatted: Line spacing: Double

Formatted: Font: (Default) Times New Roman

Formatted: Font: (Default) Times New Roman, Not Italic

Formatted: Font: (Default) Times New Roman

Formatted: Font: (Default) Times New Roman, Not Italic

- ‘I did MyFitnessPal …. and that was an absolute pain ... for me because I've got no idea about what calories are or anything. I mean anything at all, and I completely rely on [my wife] so what she used to do was cook the dinner, tell me what was in it and then half the time as I'm trying to find everything and put it into my phone and go.’

The last comment, aside from outlining a lack of knowledge with calories, suggested a dependency on the spouse to both prepare food and support the participant’s dieting attempts. Spousal support is further outlined in Theme 4, ST3.

ST2 Lifestyle barriers

Following on from the prior discussion, we uncover how lifestyle factors, in particular working patterns, can be disruptive and could eventually undermine adherence to a dietary regime.

- ‘Other **complications of life** come into that, such as what my wife eats.’
- So, I’m a nurse by trade and so I use to do long 12-hour shifts where you would struggle to be able to eat on the shift regardless of what you were trying to eat. And then at home, you’re exhausted afterwards. **You don’t want to cook particularly good things.** I’ve never found salads and fruit items particularly filling. There might be a psychological factor with that. But I would try and eat something healthy like baked potatoes, beans and a huge salad with it. Build up on the good bits with it. **Half an hour afterwards I’d be sitting there going I’m starving**.’
- ‘That's partly what my lifestyle issue is I’m out coaching tonight and I'm not going to get back until half eight and then we're going to eat then.’
- ‘You come home from work when you sit down because I've been working for eight or nine hours doing a physical job ... and I don't want to go out and do stuff, so you want to eat the simplest things which is kind of pasta or whatever **without realising the effect**.’

Theme 3: Point-of-realisation and motivators for change

All the men had a history of weight gain, and therefore FGI questioning further explored men’s perceptions and feelings regarding body and self-image, as well as how they felt they were perceived

Formatted: Font: (Default) Times New Roman

Formatted: Font: (Default) Times New Roman, Not Italic

Formatted: Font: (Default) Times New Roman

Formatted: Font: (Default) Times New Roman, 14 pt, No underline

Formatted: Font: (Default) Times New Roman

Formatted: Indent: First line: 0.25", Line spacing: Double

Formatted: Font: (Default) Times New Roman, Not Italic

Formatted: Line spacing: Double

Formatted: Font: (Default) Times New Roman, 16 pt

Formatted: Font: (Default) Times New Roman

Formatted: Indent: First line: 0.5", Line spacing: Double

by others. This theme also explores participant experiences and the point when they felt the need to act to address their increasing weight.

Although there were overlapping sub-themes, they were distinguishable in that ST1 referred to the level of dissatisfaction with oneself and the point of motivation to change, whilst ST2 related to self and external perceptions of image deemed important enough or a ‘threshold’ to trigger action.

ST1 Perception of body/self-image

There appeared to be a point where realisation or self-awareness acted as a key component for personal change. Early on it was made clear to the researchers that the men regularly observed themselves and were overwhelmingly shameful in nature, outlining a negative aesthetic perception of themselves, which in turn impacted self-confidence. The majority of the men were clearly able to recall, often in detail, these negative feelings prior to attempting weight loss.

- ‘Looking in the mirror and just feeling **absolutely disgusted with myself**.’
- ‘It's **an embarrassment** because I’m thinking, people are looking at me thinking I'm fat again **I can't believe I'm the size I am**.’
- ‘I walked past a mirror and thought, you’re a fat bastard to be perfectly blunt. A mirror, no T-shirt on and I just thought, Jesus that’s **the biggest I’ve ever been.**’
- ‘I actually do like to wear suits and the suits I wear now it’s **not flattering anymore**.’

ST2 Motivation for change

Following open discussion, participant responses focussed more towards the triggers to action and uncovered two core areas: aesthetic appeal and personal identity. Although different perspective, we can see the strong relationships with the previous theme, one referring to the level of dissatisfaction with oneself and the point of motivation to change, whilst the following theme relates to self and external perceptions of image. Responses were overwhelmingly related to negative body/image, and it was made apparent that these perceptions, induced a point in which ‘enough was enough’ and culminated in positive action, albeit often impulsive or sudden.

- ‘It was an **identity** thing; I didn’t want to be seen like this.’
- ‘So, there's a kind of **mismatch** between your identity and who you think you are and who you actually are.’
- So for me personally it wasn't about weight, I never look at weight it’s how aesthetically you look that's what I go for, which is why this is massive for me.
- ‘I **hated photos** of myself.’
- ‘So, for me personally it wasn't about weight, I never look at weight **it’s how aesthetically you look** that's what I go on which is why this is massive for me... it's not about weight I could weigh 100 kilos, 80 kilos it’s **how I think I should aesthetically look**.’
- ‘I think for me if I’m completely honest **I needed the shame**, if that makes sense.’

The second largest motivator to change related to health and usually triggered meaningful action, due a prior health scare or concerns associated with health risk.

- ‘I could feel **something was not right**, and that triggered me to start my exercises.’
- ‘I had **no awareness** of how things were (in relation to a routine health check-up).’
- ‘At home I happened to read a few cases of heart attack of people who are the same age as me and then **I really started thinking about it**, I still don't know whether that was a fear of heart attack or something that triggered me.’
- ‘And then when they said I was diabetic type 2 or whatever it was. They wanted to put me on medication, and **I said no**.’
- ‘I had to have emergency open heart surgery. That was a **big wake up call**. But even more so, the **photo was the more, bigger prompt**.’

Theme 4: Key factors for sustainable dietary control

Findings from the focus group discussion and thematic analysis uncovered potential approaches men viewed as being pivotal for continued engagement through to fruition of a positive dietary outcome. From the FGI’s it was interesting to note that very few of the men reported an approach to weight loss in the absence of peer support, particularly social engagement. Hence the

Formatted: Font: (Default) Times New Roman, Not Italic

Formatted: Indent: Left: 0.31", Line spacing: Double

Formatted: Line spacing: Double

Formatted: Font: (Default) Times New Roman

Formatted: Font: (Default) Times New Roman, Not Italic

Formatted: Font: (Default) Times New Roman, 16 pt

Formatted: Font: (Default) Times New Roman

Formatted: Indent: First line: 0.5", Line spacing: Double

concept of support structures dominated discussion, highlighting potential need for social competitiveness along with peer or partner support.

The comments were expressed as being significant enough to impact engagement with overall, meaningful lifestyle change (often attempted through dietary intervention) and was mainly attributed to their inability to exercise (and recognised as being the fundamental strategy to weight loss) which in its absence, attempts to adopt positive lifestyle change were likely futile e.g., 'Because of my disabilities I can't do much in the way of exercise.' and 'I can't put the miles in I used to.'. Picking up on the preference for exercise, further questioning revolved around the methods that could be employed in order engage the men with positive long-term lifestyle change. Here we uncovered the importance placed upon support structures, which in turn dominated discussion, with vehicles for continued motivation (and subsequent engagement) being competitive, supportive and guided in nature (ST1-ST3). Subtheme 4 culminates in the men's views as to the additional aspects that they felt are pivotal for dietary success.

ST1 'The buddy'

The most popular of these support structures was the 'buddying support system'. This suggestion was commonly acknowledged as being an important approach to long-term engagement to change for men specifically. The idea of 'competitive socialisation' was proposed as being central to sustained engagement in weight management programmes and focussed on male-centric social structures. This level of social accountability coupled with a sport or competitive element was highly noted across the FGIs.

- 'Having a **check in buddy**, somebody on the diet who is motivated enough and says "Okay, do you want to check in buddy, do you want **somebody you could talk to**?'
- 'I think having an **accountability partner** is key to weight loss' – Partnering-up.'
- 'Surround yourself with **people who are on the same mission**.'
- 'That would be certainly much easier if you were collaborating or even **competing with someone,** I think that would be an easier thing to do.'

Formatted: Font: (Default) Times New Roman, Not Italic

Formatted: Font: (Default) Times New Roman

Formatted: Font: (Default) Times New Roman, Not Italic

Formatted: Font: (Default) Times New Roman

Formatted: Font: (Default) Times New Roman, 14 pt, No underline

Formatted: Line spacing: Double

Formatted: Font: (Default) Times New Roman

Formatted: Indent: First line: 0.25", Line spacing: Double

Formatted: Font: (Default) Times New Roman, Not Italic

Formatted: Line spacing: Double

- ‘But if I knew I was going to meet a bunch of people and run around the park together I'd probably **commit more to that**.’

ST2 ‘The mentor’

Following on from the above sub-theme, in contrast to the need for social companionship (or a ‘buddy’) some interviewees suggested that direct mentorship would likely enhance accountability and therefore success. Depending upon the individual, some men preferred the need for a knowledgeable ‘mentor’ to check on progress, provide advice and motivate; accordingly, differing slightly in context to a ‘buddy’ system by re-positioning themselves into a subordinated role.

- ‘Because **it's always nice for someone to check in on you** and make sure you are sticking to what you say you are going to stick to.’
- ‘If you have a coach, if you hired a coach and contact them regularly and **they motivate you**, give you things to do and you are always checking in with them **so some sort of mentor**.’

ST3 ‘The spouse’

The requirement for male-specific tailored support strategies, whether from a knowledgeable mentor or peer was a common suggestion and yet interestingly, most men also outlined how spousal support was pivotal for sustained success and adaptation to lifestyle change. The men were vocal when expressing how favourable spousal support had been and would often expand to the point of suggesting that the diet was largely orchestrated by the partner which inadvertently suggested that the male, again, acted in a subordinated role by having the spouse prepare meals and provide advice. Furthermore, the comments also suggested that the men may not have fully immersed themselves within the dietary mindset (or did not feel a need to) and instead capitalised on the availability of spousal support to relinquish almost full control, including behavioural aspects, such as goal setting, self-motivation and self-control.

- ‘**My wife structures our meal plan**, but she does like 10 days shopping in a row. And she'll say to me, what do you want to eat on these days? And normally after I’ve just eaten, I don't

Formatted: Font: (Default) Times New Roman, 14 pt, No underline

Formatted: Font: (Default) Times New Roman

Formatted: Indent: First line: 0.25", Line spacing: Double

Formatted: Font: (Default) Times New Roman, Not Italic

Formatted: Line spacing: Double

Formatted: Font: (Default) Times New Roman, 14 pt, No underline

Formatted: Font: (Default) Times New Roman

Formatted: Indent: First line: 0.25", Line spacing: Double

Formatted: Font: (Default) Times New Roman, Not Italic

Formatted: Line spacing: Double

care, you know, I could have just eaten. But yeah, so we'll figure out what would the menu for the week. We'll ask the kids what they fancy, and then she'll go out and she'll buy it.'

- 'The snacks **she buys me** are apples rather than chocolates and stuff like that. That's half the battle.'
- '**My wife helps me now a hell of a lot** because she kicks me in the arse when I start to go for the doughnuts again.'
- '**For me, it was having the support of my wife**. Without that, I wouldn't have done it because she buys the food, she does the cooking in the evenings because I'm not home till late. 'If it wasn't for her doing that then **I wouldn't have got as far as I did**.'

ST4 Additional supportive factors

Whist recognising the importance the men placed on peer support, this was not disregarding other factors deemed important for dietary success. However, rather than having several comments supporting a single sub-theme as outlined earlier, the views for additional supportive factors, varied.

Knowledge was a recognised as primary skill when embarking and engaging with a diet but also outlined the importance of social interaction, despite being initially dismissed by the recipient.

- 'Whilst I didn't get much from the group aspect, one of the things I did appreciate from going to the groups was the knowledge I got. Even people that were doing their diet just like I was learning new things. And they were saying by the way, if you make your own sauces, it's really easy to do. You just throw this, this and this in. Oh, that's a good idea. You would get a little bit of knowledge, and you'd get that from like this little messenger group.'

Additional comments outline the array of opinions as to what is important when embarking on and sustaining a diet.

- '**Motivation**. It's all psychological as well, knowing that I've got to weigh. I don't know, there's something about it. It's acknowledgement that you've got to weigh in.'

Formatted: Font: (Default) Times New Roman, 14 pt, No underline

Formatted: Font: (Default) Times New Roman

Formatted: Indent: First line: 0.5", Line spacing: Double

Formatted: Font: (Default) Times New Roman, Not Italic

Formatted: Line spacing: Double

Formatted: Font: (Default) Times New Roman

Formatted: Font: (Default) Times New Roman, Not Italic

- ‘**Realistic achievable goals.** In 2019 I was 14 and a half stone in January 2014. Then I got married 2020 January the second I was 10 and a half, so I've lost four stone. And for my frame and my height, I think 10 and a half stone wasn't really realistic. But I managed to maintain that until COVID hit and had to stop going but I don't think my target weight which I hit was realistic.’
- ‘It's knowing it's a long journey and knowing that lesson I learned. You're only tackling the symptoms; you need to **tackle the root cause**.’
- ‘**It needs to be simple**; it can't be something that you have to think about and takes up too much time because I wouldn't bother, I would go, “Oh yes this is fun~~.~~” and three days later it's no longer fun it's a drag.’

~~**Discussion**~~

Discussion

The primary aim of this study was to understand lived experiences and male perceptions of weight loss initiatives, including support mechanisms when targeting weight loss. This may support future directions that enhance long-term weight loss sustainability specific to this demographic which is often under-represented [~~19-~~22-24~~21~~].

One of the key findings from this study was that although the men recognised the positive associations between weight gain and health risk, and the need to manage their weight, the concept of diet and the primary method of weight control (dieting), was viewed negatively by the majority. Dieting conjured up feelings of restraint and outlined restrictions on prior freedoms which in turn eroded willingness to engage. Perception of diet alone was firmly placed within the female domain, and yet for the men of this research, exercise was deemed to be the primary method for weight control, despite multi-component approaches highlighted as the most effective and preferred approach to weight loss (25~~17~~).

Formatted: Font: (Default) Times New Roman

Formatted: Font: (Default) Times New Roman

Formatted: Font: (Default) Times New Roman, Bold

Formatted: Font: +Body (Calibri), Not Bold

Formatted: List Paragraph, Justified, Line spacing: Double

Formatted: Font: 18 pt, Bold

Formatted: Font: (Default) Times New Roman

Formatted: Indent: First line: 0.5", Line spacing: Double

Formatted: Font: (Default) Times New Roman

Formatted: Font: (Default) Times New Roman

Formatted: Font: (Default) Times New Roman

Limiting factors contributing to the unsuccessful pursuit of dieting were primarily centred around the men's own experiences of diets. It was often cited by the men that they had little inclination to persist with a diet, with factors such as temptation, disinclination etc., within a few weeks of conception mentioned as the cause(s) for disengagement. Furthermore, comments outlined an inability to accommodate dietary approaches into their lifestyle, with working patterns having a significant negative impact on dietary efficacy which was compounded by insufficient cooking and food knowledge and skill, leading to a sense of frustration as to both what to cook and the effort to create something that conforms with their dietary requirements

This uncovered feelings of a general dislike of dieting, reinforced by prior experiences that induced barriers for future engagement. Furthermore, short term engagement, provided little time for acclimatisation with dietary principles and integration into daily lifestyle patterns. Lastly, several motivational components suggested as vital for meeting dietary outcomes, including the generation of self-efficacy, realistic goals, internalisation of health messages etc (~~18~~22,~~19~~26) appear to be significantly undermined if expectations and chances of meeting dietary outcomes are eroded from the onset. These negative experiences potentially present limited opportunities to engage men with weight loss in the future.

The observed negative association with diets and weight loss programmes was further echoed in terms of the notion of perceived expected failure. Responses within the FGIs noted a sense of 'generalisation' in dietary approaches to weight management programmes which appeared to be largely directed towards or by feminine connotations associated with aesthetic appeal. This generalised perception of female-centric patterns associated with diet/weight programmes can potentially overlook the importance of masculinised preference (~~20–22~~23, 24, 27). The sense of 'restriction' combined with healthy eating was also perceived in a negative light in terms of associated short-term hunger and boredom. The findings from the focus group therefore indicate that weight management programmes directed towards men should paradoxically focus away from 'diet' aspects and are more likely to be successful if driven from a male-personalised narrative. This perspective aligns with previous

Formatted: Font: (Default) Times New Roman

Formatted: Font: (Default) Times New Roman

Formatted: Font: (Default) Times New Roman

Formatted: Font: (Default) Times New Roman

interventions specific to male-gendered weight management programmes reported elsewhere (16, 2~~3–26~~8-30).

Leading on from this, barriers to engagement were typically linked to men's own experiences of diets and the unsuccessful pursuit of dieting. Whilst pertaining to diets as being an ineffective strategy, several participants, with pride were happy to express to their peers as to the number of diets they had attempted. The researchers coined the term 'serial dieters' to describe these men and the researchers attributed these announcements to a general competitiveness amongst the men, that as a motivator could, if leveraged correctly, serve to counteract common dietary practises that undermine engagement.

Although some individuals did recognise both the importance of knowledge and the large amount of conflicting narrative, there was no evidence to suggest they had made attempts to explore further for clarity and to develop their knowledge to avoid barriers to uptake and engagement. In addition, often the opinions expressed by the men, suggested that they come from a position of experience and yet, further investigation outlined a lack of understanding around dieting such as the multiple approaches to weight loss, in particular nutrition. The researcher's perceptions were that these experiences may have largely been pre-conceived ideas developed through for instance social interaction, which in turn acted as deterrents to engagement.

The men perceived women as immersed within the cultural realm of dieting. A consistent narrative associated with stereotypical gender was evident throughout all conducted focus groups. Despite previously being discussed as a facilitator to engagement, it was evident that some males felt the presence of females and female centred strategies would inhibit the opportunity for engagement and could be attributed to the perception that for men, dieting is deemed to be a feminised space where they feel self-conscious and uncomfortable. And yet, often the male participants shared how favourable female spousal support had been; to the point of suggesting that the diet was largely orchestrated by the female partner with the male acting in a participatory role and directed throughout the dietary project. This aligns with research (~~27–29~~31-33) and may stem from traditional or stereotypical marital roles, where the female is often responsible for buying and preparing food (~~30~~34). The researchers were led

Formatted: Font: (Default) Times New Roman

Formatted: Font: (Default) Times New Roman

Formatted: Font: (Default) Times New Roman

Formatted: Line spacing: Double

Formatted: Font: (Default) Times New Roman

Formatted: Font: (Default) Times New Roman

Formatted: Font: (Default) Times New Roman

Formatted: Font: (Default) Times New Roman

to a perception, that men felt their female spouse, being immersed within the dieting realm and dieting (alone) were more knowledgeable and experienced within this realm and as such, were able to relinquish control accordingly.

Despite a history of largely negative perceptions surrounding personal weight gain and viewing one's body daily, the impetus to attempt dieting would often stem from a single, significant moment in time. These instances, driven by appearance rather than health (~~24~~16), were consistently recalled with emotive language and perceived by the researchers to include underlying negative, embarrassing or shameful feelings that impacted self-confidence and promoted sub-standard aesthetic perceptions of themselves when amongst others. Despite external factors influencing the awareness and attitude of the members of the focus groups, there was limited record of responses relating to health. Consistent across internal and external perceptions, male aesthetic improvement was, on the surface, demonstrated to be a more significant measure of success than health or health parameters. In fact, potential implications to health were only discussed as internal motivators by three men, with the emphasis often related to exercise and energy levels, as opposed to diet alone.

Similarly, to aesthetic perception being utilised to motivate engagement with change, an improvement in health was only considered once a 'scare' had been encountered. Whether it was aesthetics or a health related 'scare' that fuelled a change, the loss of identity or comparison to a former version of themselves often lead the men to a threshold whereby 'enough was enough'. Prior to this point, a general lethargy was outlined amongst the men, with a tolerated, underlying disquiet with the decline in health and / or aesthetics which was insufficient to induce change. Subsequent action could be directly linked to an epiphany, however notably this change was often sudden, drastic, and thus unsustainable, ultimately resulting in little to no long-term effectiveness.

Vehicles for engagement were occasionally presented as positive and often included competitive behaviours that incentivised action and therefore transpired into relative success with dieting. Demonstrating weight loss improvements via incremental change for example a weight reduction from the previous measurement was shown to be a good motivator for continuation, and in fact the simplicity of competition, whether it was with oneself or as a wider group, became a consistent

Formatted: Indent: First line: 0.5", Line spacing: Double

Formatted: Font: (Default) Times New Roman

Formatted: Font: (Default) Times New Roman

theme throughout the FGI's. Simplicity was a concept that was picked up in multiple contexts within the FGI's, however this principle was always accompanied with competitive undertones, often referring to 'incentives' and 'challenges' and therefore perhaps indirectly could be associated with motivation to engage in a weight loss programme. Moreover, it was challenging to uncover the men's feelings surrounding goal failure or a period of disengagement with research suggesting that common reasons for drop-out of weight management programmes for men, centre around a lack of autonomy and support (~~31~~35). Accountability was a common factor discussed by the men throughout all FGI's, relating to a preference for a support structure. A highly competitive approach was cited as being useful for engagement, especially through 'buddying' where training is undertaken together to reach an agreed goal. This initially appeared to conflict with research suggesting a masculinised requirement for autonomy but further reflection by the researchers suggested that autonomy by the men was perceived as maintaining control rather than a full relinquishment of said control that would be expected within a structured commercial programme. Interestingly, one component of the 'commercial programme' that the FGI's were united in was the notion of either an informed professional, or a previous participant in the weight loss programme, who could provide mentorship and support when called upon. Further research exploring the forms of spousal dietary support provided to men, that could improve sustainability may provide a rationale for dietary programmes aimed at couples. Regardless of the support participants favoured, the mention of a 'spouse' or significant other, when describing an attempt at a weight loss programme was consistent and agreed upon by many men in the FGI's.

Limitations of the research

There are several limitations within the current study that are important considerations for contextualisation. Whilst the FGIs provided novel insight into male perceptions and lived experiences of dieting, there was relatively limited diversity (mainly married, educated, white men), and therefore future research should consider perspectives representative of other demographics for more holistic understanding. Furthermore, the researchers did not delve into the specific constructs of digital applications or male-centric weight management programs, including use of personalised technology and behavioural coaching initiatives. Additionally, whilst the concept of dieting was discussed, we did

Formatted: Font: (Default) Times New Roman

Formatted: Font: (Default) Times New Roman

Formatted: Font: (Default) Times New Roman, 16 pt, Not Italic

Formatted: Line spacing: Double

Formatted: Font: (Default) Times New Roman, 14 pt, Not Italic

Formatted: Font: (Default) Times New Roman

Formatted: Indent: First line: 0.5", Line spacing: Double

not pursue aspects within the diet such as dietary types or diet quality. Future research should therefore consider such aspects with a view to personalising programs specific to male-centered objectives. It was also challenging to uncover the men's feelings surrounding goal failure or a period of disengagement and this was understandable given the requirement to outline, among other men their inability to achieve a particular outcome. Individual interviews may have been more successful in uncovering the men's feelings.

Translational applications

1. Specific to men, diets and dieting are typically perceived in a negative way. Therefore, any strategy purposefully targeting male-centric weight management should focus on male narratives including social competitiveness and tailored aesthetic gains, ahead of health-specific requirements.
2. The relative importance of weight loss is perceived as less important in the men interviewed, inferring that for a weight management programme to be successful, strategies could employ a more 'covert' lifestyle approach (again highlighting social or individual competitiveness) whilst focusing on health-related risk to further introduce a proactive approach.
3. Barriers to change pertinent to male narratives need to be considered as part of a weight management strategy, with 'opportunistic simplicity' potentially being key.
4. A noted 'point of realisation' could be brought to the individual's attention earlier in the behavioural change cycle, which could lead to earlier incentivisation to induce effective engagement with a programme.
5. Strategies for longer-term male success should focus on simplicity coupled with quick-wins, early rewards and a tailored degree of social competitiveness.
6. A novel aspect of this study was the identification of the importance of tailored interplay between buddy-mentor-spousal support as fundamental to early and sustained success, with the emphasis likely towards spousal support.

Conclusions

Formatted: Font: (Default) Times New Roman, 16 pt, Not Italic

Formatted: Line spacing: Double

Formatted: Font: (Default) Times New Roman

Formatted: Font: (Default) Times New Roman, 18 pt

Formatted: Font: 18 pt

Formatted: Font: (Default) Times New Roman, 18 pt

This research proved instrumental in understanding male perceptions around the realm of dieting. Drivers for change stemmed primarily from aesthetics although a health 'scare' would help drive a person to adapt meaningful lifestyle changes. Consensus outlined a general uneasiness with prescribed dietary approaches which suggested a distancing from the female realm of dieting. However, discourse also suggested the men persistently engaged with diets to induce weight loss (albeit superficial in nature). A primary factor identified for longer-term dietary success was peer support with three primary relationships outlined: mentor, buddying and spouse. Recruitment of spousal support was uncovered as likely the most effective method for supporting longer term engagement and goal-achievement, facilitated through the relegation of control to the spouse. Findings from this study highlight that sustainable lifestyle change specific to male populations need to be gender tailored and not generalised.

Acknowledgements

The authors wish to thank the participants for their time and commitment in supporting this research.

~~Author Contributions: Conceptualisation, M.C., J.L., J.R.; methodology, M.C., J.L., J.R.; formal analysis, M.C., J.L., J.R.; writing – original draft preparation, M.C., J.L., J.R.; writing – review and editing, M.C., J.L., J.R; project administration, M.C., J.R.; funding acquisition, J.R., M.C. All authors have read and agreed to the published version of the manuscript.~~

~~Funding: This research received no external funding, although The APC was funded by Anglia Ruskin University.~~

~~Institutional Review Board Statement: The study was conducted in accordance with the Decla-ration of Helsinki, and approved by the School of Psychology and Sports Science Ethics Com-mittee (ethics approval number: ETH2122-0617, 06/06/2022).~~

~~Informed Consent Statement: Informed consent was obtained from all subjects involved in the study.~~

Formatted: Font: (Default) Times New Roman

Formatted: Indent: First line: 0.5", Line spacing: Double

Formatted: Font: 18 pt, Bold

Formatted: Line spacing: Double

Formatted: Font: (Default) Times New Roman

Data Availability Statement: The data presented in this study are available on request from the corresponding author.

Conflicts of Interest: The authors declare no conflict of interest.

References

1. Lowe DA, Wu N, Rohdin-Bibby L, Moore AH, Kelly N, Liu YE, et al. Effects of Time-Restricted Eating on Weight Loss and Other Metabolic Parameters in Women and Men With Overweight and Obesity: The TREAT Randomized Clinical Trial. JAMA Intern Med. 2020 Nov 1;180(11):1491.

2. Soliman GA. Intermittent fasting and time-restricted eating role in dietary interventions and precision nutrition. Front Public Health. 2022 Oct 28;10:1017254.

3. Malhotra V, Sawal A. Metabolic Effects of Ketogenic Diets and Their Utilization in Obesity Management: A Systematic Review. Cureus [Internet]. 2023 Mar 26 [cited 2023 Dec 3]; Available from: https://www.cureus.com/articles/117114-metabolic-effects-of-ketogenic-diets-and-their-utilization-in-obesity-management-a-systematic-review

4. Choi YJ, Jeon SM, Shin S. Impact of a Ketogenic Diet on Metabolic Parameters in Patients with Obesity or Overweight and with or without Type 2 Diabetes: A Meta-Analysis of Randomized Controlled Trials. Nutrients. 2020 Jul 6;12(7):2005.

5. Wewege M, Van Den Berg R, Ward RE, Keech A. The effects of high-intensity interval training vs. moderate-intensity continuous training on body composition in overweight and obese adults: a systematic review and meta-analysis. Obesity Reviews. 2017 Jun;18(6):635–46.

6. Dent R, McPherson R, Harper ME. Factors affecting weight loss variability in obesity. Metabolism. 2020 Dec;113:154388.

Formatted: Font: (Default) Times New Roman, 18 pt

Formatted: Font: (Default) Times New Roman

Formatted: Normal, Justified, Line spacing: Double

7. NICE. Obesity prevention [Internet]. National Institute for Health and Care Excellence; 2006. Report No.: CG43. Available from: https://www.nice.org.uk/guidance/cg43

8. National Institute for Health and Care Excellence. Obesity: identification, assessment and management. 2022 Sep. Report No.: CG189.

9. Anderson JW, Konz EC, Frederich RC, Wood CL. Long-term weight-loss maintenance: a meta-analysis of US studies. The American Journal of Clinical Nutrition. 2001 Nov 1;74(5):579–84.

10. Thomas JG, Bond DS, Phelan S, Hill JO, Wing RR. Weight-Loss Maintenance for 10 Years in the National Weight Control Registry. American Journal of Preventive Medicine. 2014 Jan;46(1):17–23.

11. Phelan S, Halfman T, Pinto AM, Foster GD. Behavioral and Psychological Strategies of Long-Term Weight Loss Maintainers in a Widely Available Weight Management Program. Obesity. 2020 Feb;28(2):421–8.

12. Spreckley M, Seidell J, Halberstadt J. Perspectives into the experience of successful, substantial long-term weight-loss maintenance: a systematic review. International Journal of Qualitative Studies on Health and Well-being. 2021 Jan 1;16(1):1862481.

13. Hartmann-Boyce J, Boylan AM, Jebb SA, Aveyard P. Experiences of Self-Monitoring in Self-Directed Weight Loss and Weight Loss Maintenance: Systematic Review of Qualitative Studies. Qual Health Res. 2019 Jan;29(1):124–34.

14. Greaves C, Poltawski L, Garside R, Briscoe S. Understanding the challenge of weight loss maintenance: a systematic review and synthesis of qualitative research on weight loss maintenance. Health Psychology Review. 2017 Apr 3;11(2):145–63.

15. Braun V, Clarke V. Using thematic analysis in psychology. Qualitative Research in Psychology. 2006 Jan;3(2):77–101.

16. Vaismoradi M, Turunen H, Bondas T. Content analysis and thematic analysis: Implications for conducting a qualitative descriptive study: Qualitative descriptive study. Nurs Health Sci. 2013 Sep;15(3):398–405.

17. National Institute for Health and Care Excellence. Overweight and obesity management NICE guideline [NG246] [Internet]. NICE; 2025 Jan [cited 2025 Jan 16]. Available from: https://www.nice.org.uk/guidance/ng246/evidence

18. Dicker D, Alfadda AA, Coutinho W, Cuevas A, Halford JCG, Hughes CA, et al. Patient motivation to lose weight: Importance of healthcare professional support, goals and self-efficacy. European Journal of Internal Medicine. 2021 Sep;91:10–6.

19. Skelly LJ, Smyth PP, Donnelly MP, Leslie JC, Leader G, Simpson L, et al. Factors that potentially influence successful weight loss for adults with intellectual disabilities: A qualitative comparison. J Intellect Disabil. 2021 Dec;25(4):458–75.

20. Tsai SA, Lv N, Xiao L, Ma J. Gender Differences in Weight-Related Attitudes and Behaviors Among Overweight and Obese Adults in the United States. Am J Mens Health. 2016 Sep;10(5):389–98.

21. Morgan PJ, Collins CE, Plotnikoff RC, McElduff P, Burrows T, Warren JM, et al. The SHED-IT community trial study protocol: a randomised controlled trial of weight loss programs for overweight and obese men. BMC Public Health. 2010 Dec;10(1):701.

22. Levi A, Chan KK, Pence D. Real Men Do Not Read Labels: The Effects of Masculinity and Involvement on College Students' Food Decisions. Journal of American College Health. 2006 Sep 1;55(2):91–8.

23. Hunt K, Wyke S, Gray CM, Anderson AS, Brady A, Bunn C, et al. A gender-sensitised weight loss and healthy living programme for overweight and obese men delivered by Scottish Premier League football clubs (FFIT): a pragmatic randomised controlled trial. The Lancet. 2014 Apr;383(9924):1211–21.

24. Cortnage M, Pringle A. Onset of Weight Gain and Health Concerns for Men: Findings from the TAP Programme. International Journal of Environmental Research and Public Health [Internet]. 2022;19(1). Available from: https://www.mdpi.com/1660-4601/19/1/579

25. Pringle A, Zwolinsky S, McKenna J, Robertson S, Daly-Smith A, White A. Health improvement for men and hard-to-engage-men delivered in English Premier League football clubs. Health Education Research. 2014 Jun 1;29(3):503–20.

26. Robertson C, Avenell A, Boachie C, Stewart F, Archibald D, Douglas F, et al. Should weight loss and maintenance programmes be designed differently for men? A systematic review of long-term randomised controlled trials presenting data for men and women: The ROMEO project. Obesity Research & Clinical Practice. 2016 Jan;10(1):70–84.

27. Albanese AM, Huffman JC, Celano CM, Malloy LM, Wexler DJ, Freedman ME, et al. The role of spousal support for dietary adherence among type 2 diabetes patients: a narrative review. Social Work in Health Care. 2019 Mar 16;58(3):304–23.

28. Beverly EA, Wray LA. The role of collective efficacy in exercise adherence: a qualitative study of spousal support and Type 2 diabetes management. Health Education Research. 2010 Apr 1;25(2):211–23.

29. Tripathee S, Sweeting H, Chambers S, Maclean A. How men receive and utilise partner support when trying to change their diet and physical activity within a men's weight management programme. BMC Public Health. 2020 Dec;20(1):199.

30. Wong M, Gucciardi E, Li L, Grace SL. Gender and Nutrition Management in Type 2 Diabetes. Canadian Journal of Dietetic Practice and Research. 2005 Dec;66(4):215–20.

31. Archibald D, Douglas F, Hoddinott P, van Teijlingen E, Stewart F, Robertson C, et al. A qualitative evidence synthesis on the management of male obesity. BMJ Open. 2015 Oct;5(10):e008372.

1. Lowe DA, Wu N, Rohdin-Bibby L, Moore AH, Kelly N, Liu YE, et al. Effects of Time-Restricted Eating on Weight Loss and Other Metabolic Parameters in Women and Men With Overweight and Obesity: The TREAT Randomized Clinical Trial. JAMA Intern Med. 2020 Nov 1;180(11):1491-1499. doi: 10.1001/jamainternmed.2020.4153.
2. Soliman GA. Intermittent fasting and time-restricted eating role in dietary interventions and precision nutrition. Front Public Health. 2022 Oct 28;10:1017254. doi: 10.3389/fpubh.2022.1017254.
3. Malhotra V, Sawal A. Metabolic Effects of Ketogenic Diets and Their Utilization in Obesity Management: A Systematic Review. Cureus. 2023 Mar 26;15(3):e36720. doi: 10.7759/cureus.36720.
4. Choi YJ, Jeon SM, Shin S. Impact of a Ketogenic Diet on Metabolic Parameters in Patients with Obesity or Overweight and with or without Type 2 Diabetes: A Meta-Analysis of Randomized Controlled Trials. Nutrients. 2020 Jul 6;12(7):2005. doi: 10.3390/nu12072005.
5. Wewege M, van den Berg R, Ward RE, Keech A. The effects of high-intensity interval training vs. moderate-intensity continuous training on body composition in overweight and obese adults: a systematic review and meta-analysis. Obes Rev. 2017 Jun;18(6):635-646. doi: 10.1111/obr.12532.
6. Dent R, McPherson R, Harper ME. Factors affecting weight loss variability in obesity. Metabolism. 2020 Dec;113:154388. doi: 10.1016/j.metabol.2020.154388.
7. NICE. Obesity prevention [Internet]. National Institute for Health and Care Excellence; 2006. Report No.: CG43. Available from: https://www.nice.org.uk/guidance/cg43.
8. National Institute for Health and Care Excellence. Obesity: identification, assessment and management. 2022 Sep. Report No.: CG189.
9. Anderson JW, Konz EC, Frederich RC, Wood CL. Long-term weight-loss maintenance: a meta-analysis of US studies. Am J Clin Nutr. 2001 Nov;74(5):579-84. doi: 10.1093/ajcn/74.5.579.
10. Thomas JG, Bond DS, Phelan S, Hill JO, Wing RR. Weight-loss maintenance for 10 years in the National Weight Control Registry. Am J Prev Med. 2014 Jan;46(1):17-23. doi: 10.1016/j.amepre.2013.08.019.

Formatted: Font: (Default) Times New Roman

Formatted: List Paragraph, Numbered + Level: 1 + Numbering Style: 1, 2, 3, … + Start at: 1 + Alignment: Left + Aligned at: 0.25" + Indent at: 0.5"

Formatted: Font: (Default) Times New Roman

Formatted: Font: (Default) Times New Roman

Formatted: Font: (Default) Times New Roman

Formatted: Font: (Default) Times New Roman

Formatted: Font: (Default) Times New Roman

Field Code Changed

Formatted: Hyperlink, Font: (Default) Times New Roman

Formatted: Font: (Default) Times New Roman

Formatted: Font: (Default) Times New Roman

Formatted: Font: (Default) Times New Roman

11. Phelan S, Halfman T, Pinto AM, Foster GD. Behavioral and Psychological Strategies of Long-Term Weight Loss Maintainers in a Widely Available Weight Management Program. Obesity (Silver Spring). 2020 Feb;28(2):421-428. doi: 10.1002/oby.22685.

12. Spreckley M, Seidell J, Halberstadt J. Perspectives into the experience of successful, substantial long-term weight-loss maintenance: a systematic review. Int J Qual Stud Health Well-being. 2021 Dec;16(1):1862481. doi: 10.1080/17482631.2020.1862481.

13. Hartmann-Boyce J, Boylan AM, Jebb SA, Aveyard P. Experiences of Self-Monitoring in Self-Directed Weight Loss and Weight Loss Maintenance: Systematic Review of Qualitative Studies. Qual Health Res. 2019 Jan;29(1):124-134. doi: 10.1177/1049732318784815.

14. Greaves C, Poltawski L, Garside R, Briscoe S. Understanding the challenge of weight loss maintenance: a systematic review and synthesis of qualitative research on weight loss maintenance. Health Psychol Rev. 2017 Jun;11(2):145-163. doi: 10.1080/17437199.2017.1299583.

15. Patton, M. 2002. Qualitative research and evaluation methods. 3rd Edition. Sage Publications; Thousand Oaks, CA.

16. Cortnage M, Pringle A. Onset of Weight Gain and Health Concerns for Men: Findings from the TAP Programme. Int J Environ Res Public Health. 2022 Jan 5;19(1):579. doi: 10.3390/ijerph19010579.

17. Donnachie C, Sweeting H, Hunt K. A Qualitative Study on Young Men's Experiences of Intentional Weight-Gain. Int J Environ Res Public Health. 2023 Feb 14;20(4):3320. doi: 10.3390/ijerph20043320.

18. Lin YC, Liu PC, Chou FF, Hou IC, Chou CC, Chen CW, et al. Beliefs underlying weight control behaviors among adolescents and emerging adults living with obesity: an elicitation qualitative study. BMC Public Health. 2024 Nov 27;24(1):3292. doi: 10.1186/s12889-024-20677-5.

19. Guest G, Namey E, Taylor J, Eley N, and McKenna K. Comparing focus groups and individual interviews: findings from a randomized study. Int J Social Res Methodol. 2017; 20(6), 693–708. https://doi.org/10.1080/13645579.2017.1281601.

20. Braun V, and Clarke V. Using thematic analysis in psychology. Qual Res Psychol. 2006; 3(2): 77–101. doi: 10.1191/1478088706qp063oa.

21. Vaismoradi M, Turunen H, Bondas T. Content analysis and thematic analysis: Implications for conducting a qualitative descriptive study. Nurs Health Sci. 2013 Sep;15(3):398-405. doi: 10.1111/nhs.12048.

22. Skelly LJ, Smyth PP, Donnelly MP, Leslie JC, Leader G, Simpson L, et al. Factors that potentially influence successful weight loss for adults with intellectual disabilities: A qualitative comparison. J Intellect Disabil. 2021 Dec;25(4):458-475. doi: 10.1177/1744629520931681.

23. Tsai SA, Lv N, Xiao L, Ma J. Gender Differences in Weight-Related Attitudes and Behaviors Among Overweight and Obese Adults in the United States. Am J Mens Health. 2016 Sep;10(5):389-98. doi: 10.1177/1557988314567223.

24. Morgan PJ, Collins CE, Plotnikoff RC, McElduff P, Burrows T, Warren JM, et al. The SHED-IT community trial study protocol: a randomised controlled trial of weight loss programs for overweight and obese men. BMC Public Health. 2010 Nov 16;10:701. doi: 10.1186/1471-2458-10-701.

25. National Institute for Health and Care Excellence. Overweight and obesity management NICE guideline [NG246] [Internet]. NICE; 2025 Jan. Available from: https://www.nice.org.uk/guidance/ng246/evidence.

26. Dicker D, Alfadda AA, Coutinho W, Cuevas A, Halford JCG, Hughes CA, et al. Patient motivation to lose weight: Importance of healthcare professional support, goals and self-efficacy. Eur J Intern Med. 2021 Sep;91:10-16. doi: 10.1016/j.ejim.2021.01.019.

27. Levi A, Chan KK, Pence D. Real men do not read labels: the effects of masculinity and involvement on college students' food decisions. J Am Coll Health. 2006 Sep-Oct;55(2):91-8. doi: 10.3200/JACH.55.2.91-98.

Formatted: Font: (Default) Times New Roman

Formatted: Font: (Default) Times New Roman

Formatted: Font: (Default) Times New Roman

Formatted: Font: (Default) Times New Roman

Formatted: Font: (Default) Times New Roman

Formatted: Font: (Default) Times New Roman

Formatted: Hyperlink, Font: (Default) Times New Roman

Field Code Changed

Formatted: Font: (Default) Times New Roman

28. Hunt K, Wyke S, Gray CM, Anderson AS, Brady A, Bunn C, et al. A gender-sensitised weight loss and healthy living programme for overweight and obese men delivered by Scottish Premier League football clubs (FFIT): a pragmatic randomised controlled trial. Lancet. 2014 Apr 5;383(9924):1211-21. doi: 10.1016/S0140-6736(13)62420-4.

29. Pringle A, Zwolinsky S, McKenna J, Robertson S, Daly-Smith A, White A. Health improvement for men and hard-to-engage-men delivered in English Premier League football clubs. Health Educ Res. 2014 Jun;29(3):503-20. doi: 10.1093/her/cyu009.

30. Robertson C, Avenell A, Boachie C, Stewart F, Archibald D, Douglas F, et al. Should weight loss and maintenance programmes be designed differently for men? A systematic review of long-term randomised controlled trials presenting data for men and women: The ROMEO project. Obes Res Clin Pract. 2016 Jan-Feb;10(1):70-84. doi: 10.1016/j.orcp.2015.04.005.

31. Albanese AM, Huffman JC, Celano CM, Malloy LM, Wexler DJ, Freedman ME, et al. The role of spousal support for dietary adherence among type 2 diabetes patients: a narrative review. Soc Work Health Care. 2019 Mar;58(3):304-323. doi: 10.1080/00981389.2018.1563846.

32. Beverly EA, Wray LA. The role of collective efficacy in exercise adherence: a qualitative study of spousal support and type 2 diabetes management. Health Educ Res. 2010 Apr;25(2):211-23. doi: 10.1093/her/cyn032.

33. Tripathee S, Sweeting H, Chambers S, Maclean A. How men receive and utilise partner support when trying to change their diet and physical activity within a men's weight management programme. BMC Public Health. 2020 Feb 7;20(1):199. doi: 10.1186/s12889-020-8213-z.

34. Wong M, Gucciardi E, Li L, Grace SL. Gender and nutrition management in type 2 diabetes. Can J Diet Pract Res. 2005 Winter;66(4):215-20. doi: 10.3148/66.4.2005.215.31.

35. Archibald D, Douglas F, Hoddinott P, van Teijlingen E, Stewart F, Robertson C, Boyers D, Avenell A. A qualitative evidence synthesis on the management of male obesity. BMJ Open. 2015 Oct 12;5(10):e008372. doi: 10.1136/bmjopen-2015-008372.

Supporting information

Formatted: Font: (Default) Times New Roman

Formatted: Font: (Default) Times New Roman

Formatted: Font: (Default) Times New Roman

Formatted: Font: (Default) Times New Roman

Formatted: Font: (Default) Times New Roman

Formatted: Font: (Default) Times New Roman

Formatted: Font: (Default) Times New Roman

Formatted: Font: Bold

S1 File. Appendix showing general framework for focus group questions.

Formatted: Font: 14 pt

Formatted: Indent: Left: 0.25", Line spacing: Double

Formatted: Font: (Default) Times New Roman, 14 pt

PONE-D-25-58139
Male perceptions and experiences of weight loss initiatives: recommendations towards sustainable lifestyle practice.
PLOS One

Authors replies to comments

Journal Requirement comments

1) Please ensure that your manuscript meets PLOS ONE's style requirements, including those for file naming.
Authors response: Thank you for the information. We have duly looked at both pdfs and fully reviewed/revised the manuscript in alignment with PLOS ONEs style requirements. This has also involved a complete revision for references.

2) When completing the data availability statement of the submission form, you indicated that you will make your data available on acceptance. We strongly recommend all authors decide on a data sharing plan before acceptance, as the process can be lengthy and hold up publication timelines. Please note that, though access restrictions are acceptable now, your entire data will need to be made freely accessible if your manuscript is accepted for publication. This policy applies to all data except where public deposition would breach compliance with the protocol approved by your research ethics board. If you are unable to adhere to our open data policy, please kindly revise your statement to explain your reasoning and we will seek the editor's input on an exemption. Please be assured that, once you have provided your new statement, the assessment of your exemption will not hold up the peer review process.
Authors response: Thank you for this information. This statement will be revised during the resubmission process. As this was a qualitative study involving transcription of focus groups, full transcriptions can be made accessible.
3) Your ethics statement should only appear in the Methods section of your manuscript. If your ethics statement is written in any section besides the Methods, please delete it from any other section.
Authors response: Thank you for this information. We have removed the information at the end of manuscript and only kept a clear ethics statement in the Methods section as requested.

4) We note that this data set consists of interview transcripts. Can you please confirm that all participants gave consent for interview transcript to be published?
Authors response: Yes indeed, all participants provided consent for interview transcripts to be published. There are no potentially identifying information,

and where only first names were mentioned, these were removed during the thematic analysis process so that individuals could not be identified in any context.

5) Please include captions for your Supporting Information files at the end of your manuscript, and update any in-text citations to match accordingly.
Authors response: Thank you for noting this, we have updated the manuscript accordingly.

6) If the reviewer comments include a recommendation to cite specific previously published works, please review and evaluate these publications to determine whether they are relevant and should be cited. There is no requirement to cite these works unless the editor has indicated otherwise.
Author response: we have noted this comment.

Response to reviewers:

Reviewer #1: Thank you for giving me the opportunity to review this paper. This study addresses a significant health issue, but there are several key concerns regarding the methodology:

1. Please clarify the specific type of qualitative study and provide justification for this choice.

Authors response: Firstly thank you for the review and commentary, which presented interesting and beneficial research team discussion. In constructing the paper, we aimed to be as concise as possible in the early stages, knowing that the nature of thematic analysis findings can be longer. In doing so, it is evident that we may have overlooked methodological detail which was indeed evident during the design and execution of the study. As the manuscript has been in submission since October, it has been a while since we have reviewed the paper so this has been a useful exercise in itself.

The study employed a qualitative descriptive study format using focus groups to explore mens perceptions and experiences of weight loss initiatives with the intention of informing practices which may be more sustainable and beneficial in the longer term. Based on previous work within our group, we also recognised the challenge faced when recruiting participants particularly when more sensitive topics are presented (such as weight loss). As such use of semi-structured interviews within focus group sessions was deemed appropriate in providing an informal, safe space for participants to openly and honestly engage. Indeed, topics which may be somewhat sensitive to discuss may be more easier to explore when there is peer support or similar shared experiences. The study type was based on

previously published research by our group (reference 24) and follows similar guidelines and approaches outlined elsewhere (see: https://pubmed.ncbi.nlm.nih.gov/40374218/ and https://pmc.ncbi.nlm.nih.gov/articles/PMC10797665/). We have revised the beginning of the 'Focus groups' section in the methods to reflect revised modifications to the manuscript.

Field Code Changed

2. The study setting needs more detail. Please describe the geographic or contextual setting and explain how participants were recruited, what do you mean of via social media or advertisements?

Authors response: Again, thank you for the comment. It is important to note that we have followed approaches similar to other published articles (https://pubmed.ncbi.nlm.nih.gov/37339012/ , https://pubmed.ncbi.nlm.nih.gov/35010838/ , https://www.tandfonline.com/doi/full/10.1080/1612197X.2015.1114504), some of which do not go into as much detail (including one published in this journal: https://journals.plos.org/plosone/article?id=10.1371/journal.pone.0076089). However, in fully responding to this comment, as this was an exploratory study, we aimed to undertake local focus group interviews (specific to the Anglia Region, Cambridgeshire and Chelmsford where we are based in the UK). However, for those who could not attend the University, we also held an online Teams forum for practicality. All face-to-face interviews were held in a wide-spaced meeting room environment on the University campus, often used for general or public meetings. Interviews were undertaken with all participants sat in a circle, facing each other to foster equality, open communication, and active listening throughout. In all interviews members of the research team were also positioned amongst the participants within the circle.

Participants were recruited via convenience sampling utilising several approaches, mainly word of mouth and personal contacts, alongside use of social media (researcher promotion via public advert on facebook or Linkedin). The same advert was also promoted via our local University intranet and media network (i.e. study adverts promoted internally via advertising screens and internal university marketing). In addition, study adverts were promoted via local government authority (Cambridgeshire Region) health initiatives following gatekeeper approval. Via adverts we employed purposive and snowballing sampling approaches. We have added content to the methods section to clarify these points.

3. In terms of participant selection, please provide comprehensive inclusion and exclusion criteria, as the current description is too brief.

Authors response: The initial information provided in the manuscript is correct based on the general remit employed during the ethical approval process. We purposely wanted to keep the inclusion remit broad so as not to restrict study participation. However, we have included additional information in the revised manuscript to further clarify study inclusion/exclusion in line with overall study conduct.

For study inclusion, participants were required to be male, aged > 30 years, in general good health, and able to communicate in English. Participants were also required to have a lived experience of being overweight, having previously undertaken a weight-loss programme or were actively aiming to lose weight in the near future, and therefore willing and able to contribute to discussions around perceptions and experiences of weight loss initiatives within a semi-structured focus group discussion setting. Participants were excluded if they were: < 30 years, had an underlying medical condition that would make participation unsafe or inappropriate, or had no prior engagement with weight loss behaviours. Participants were also excluded if they unwilling to be audio-recorded or unable to provide informed consent.

4. Regarding sampling, please detail the sampling framework and justify the sample size with relevant references.
Authors response: Our study employed a criterion-based purposive sampling framework. Participants were selected based on predefined characteristics that were directly aligned with the study aims and inclusion/exclusion criteria. Qualitative studies frequently employ purposeful sampling, a strategy in which participants are chosen based on their capacity to contribute detailed, experience-based information that aligns closely with the study's aims (Patton, 2002). Reference: Patton, M. 2002. Qualitative research and evaluation methods. 3rd Edition. Sage Publications; Thousand Oaks, CA.

Sample size was based on prior experience (https://pubmed.ncbi.nlm.nih.gov/35010838/, incorporating 8 participants), and further justified through reference to numerous publications employing a similar methodological approach (e.g. https://www.mdpi.com/1660-4601/20/4/3320, n=13; https://www.mdpi.com/1660-4601/22/12/1796, 2 FGIs only and n=14; https://link.springer.com/article/10.1186/s12889-024-20677-5, n= 15 adults; https://www.tandfonline.com/doi/pdf/10.1080/17482631.2024.2406099, n=10). In the current study, we undertook several focus groups to ascertain theme saturation. The overall included sample size (n=24) is considered robust in alignment with other published research within this domain.

5. For data collection tools, please explain how the semi-structured interview questions were developed, their number, and whether any pilot testing was conducted.

Authors response: The semi-structured interview question framework (see S1 File) was developed to align closely with the primary aim of the study which was to explore male lived experiences, perceptions, motivations, and barriers related to weight-loss initiatives. The researcher's questions were developed based on the following:

- **The existing literature on male engagement with weight-loss programmes, relevant behavioural change strategies, relevant prevalence / incidence data and gender-specific barriers.**
- **Theoretical considerations in including hegemonic practices, male help seeking behaviours, reports on lifestyle behaviour change, masculine norms, and both general and specific weight-management challenges / facilitators.**
- **The research team's prior work, which highlighted gaps in understanding men's motivations and experiences with weight loss and management.**

During initial preparation, the research team discussed what the key objectives and themes were, and drafted open-ended questions to encourage more detailed responses and wider group discussion. Questions were informally piloted with academic colleagues during this initial preparation phase to ensure alignment with the underlying objectives of the study.

The resulting focus group framework consisted of a set of pre-determined open-ended questions, designed to prompt discussion around dieting experiences, perceived barriers, motivations for change, support mechanisms, and views on long-term weight-loss sustainability. The underlying framework comprised 3 general sections in logical order (views/opinions on weight management; knowledge and understanding around weight loss initiatives; and critical insights and thoughts towards longer term positive change), and 14 prompt questions. The researcher's accounted for the use of additional probing questions to further explore responses to improve depth of understanding, consistent with the semi-structured approach. Based on the above, we were able to ensure that the questions were grounded in evidence, relevant, and capable of eliciting rich experiential data.

6. Since the study employed focus group discussions, please justify why this method was chosen, especially considering the sensitive nature of the topic.

Author response: Focus group discussions were selected as the primary data collection method because they enabled the researchers to explore the experiences, social meanings, and the collective narratives surrounding weight loss among men >30 years. The researchers wished to understand male perceptions, motivations, and barriers to weight-loss engagement. Topics that are shaped not only by individual experiences and beliefs but by social norms, masculine identities, and peer group influences. Focus groups are well-suited to capturing these relational perspectives, as they provide an environment for participants to respond to, challenge, and build upon each other's perspectives. In doing so these help to reveal collective perspectives, beliefs, experienced and social norms detected via thematic analysis.

Based on prior work from this group, we were initially conscious of the sensitive nature of the topic, particularly from a male context which is somewhat under-represented. Indeed, findings from other studies highlight that sensitive topics may often be more freely disclosed or discussed within informal focus group settings where peer support or those having similar experiences become more comfortable to share perspectives and experiences (https://www.tandfonline.com/doi/full/10.1080/13645579.2017.1281601).

7. Concerning the study procedure, please elaborate on how social media was utilized, the recruitment timeline, the interviewers involved, the language used, and the overall recruitment and interviewing process.

Author response: As outlined within comment 2, participants were recruited via convenience sampling utilising several approaches, mainly word of mouth and personal contacts, alongside use of social media (researcher promotion via public advert on Facebook or Linkedin)[See comment 2 for further information]. In terms of social media, an online advert depicting the study aims and objectives, participants requirements and what to do next if interested was posted via the researchers own social media networks (e.g. Facebook, LinkedIn). This was part of the strategy to maximise reach within the local region via personal connections. The recruitment timeline was undertaken over a 7 week period preceding start of the first focus group. All researchers were involved in supporting and conducting the focus group interviews. MC is a qualitative public health researcher with considerable experience in this domain, particularly relevant to male participants and lived experienced. As such, MC was the key moderator for each focus group. Both JR and JL are experienced nutrition researchers, with experience in conducting research (including focus groups) pertinent to weight management and male participants. As such, JR acted as an objective moderator to ensure all themes/objectives were addressed. JL supported this role, and employed prompt or probing questions in line with the framework used to encourage further discussion. All focus group interviews were conducted verbally in English. For two

of the FGIs, these were held face-to-face within the same University meeting room (purposefully designed to be an informal, relaxed, wide, open-planned setting). The room was set out in an open-planned, circular layout to encourage and facilitate open discussion. Following initial welcome and provision of name badges, the research team checked participants had read and fully understood the 'Participant Information Sheet' which overviewed the study in detail, emphasising that participation was voluntary, and that they could withdraw at any point without judgement. Following this, participants were required to complete written informed consent prior to starting the interview itself. All interviews lasted approximately 1hr and were audio recorded using a TileRec Digital USB 3.0 Type C voice activated recorder. The research team utilised the focus group questions framework as a means to provide logical structure and organisation to the interviews.

8. For data transcription and analysis, please clarify who handled transcription, whether translation was needed, and how potential biases were minimized.

Author response: in line with the policy of our Institutional ethics committee, when undertaking audio recorded interviews, audio files must be transcribed verbatim using a university-approved service. As such, once all focus groups were completed, all audio files (MP3) were uploaded to a university-approved provider, with word document transcripts provided to the research team following transcription completion. As all interviews were conducted in English, no translation was needed. When reviewing the transcripts, coding was initially conducted by two researchers (MC and JL) with second-phase confirmation by a third researcher (JR). As such, the use of duplicate coders, along with a second-phase confirmation process was employed as part of a triangulation process to minimise potential bias. Furthermore, by the fourth focus group, similar contexts, perspectives and experiences were noted by the research team, and confirmed during the thematic analysis process inferring that saturation had likely been reached.

9. Finally, regarding trustworthiness and rigor, please elaborate on how credibility, transferability, dependability, and confirmability were ensured.

Author response: To address this, we reverted back to early research by Lincoln and Guba where these dimensions are sourced (**Lincoln, Y.S. and Guba, E.G. (1986), But is it rigorous? Trustworthiness and authenticity in naturalistic evaluation. New Directions for Program Evaluation, 1986: 73-84. https://doi.org/10.1002/ev.1427) .**

In addressing credibility (internal validity) we employed several focus groups as a means to determine if similar experiences/perceptions were noted amongst the groups. As noted in previous comments, the use of focus groups was also pertinent to allow participants to respond to and build on each other's ideas, thus helping to reveal collective beliefs and social norms about topics meanwhile stimulating each other's experiences or perspectives. Focus groups were undertaken in a similar manner, utilising a facilitating lead moderator and objective moderator to ensure neutrality, consistency and rigor. Furthermore, in undertaking the focus groups, we also used confirmation questions or statements to ensure that the information provided was correct and clear. We also standardised the duration of each interview to ensure participants had a similar timeframe and context between focus groups.

In addition, we employed a recognised stage process (Braun and Clarke, 2006) to standardise the methodological approach, and used a clear triangulation approach during the thematic analysis coding process to minimise risk of internal bias and ensure multiple opinions were taken on board before arriving at a consensus.

In addressing transferability (external validity), we have provided comprehensive information relating to operational set up and delivery of the focus group interviews from context, recruitment/ inclusion/exclusion criteria, interview setting, and development of a focus group question framework. In doing so, our objective is to be as fully transparent in the process as possible for generalisability. We also noted earlier on that we employed a purposive sampling framework, aiming to also capture a range of ages (>30 years), educational backgrounds and marital status (see Table 1 in the manuscript). Within the focus group interviews we did notice that sometimes when one person would initiate a response to a moderator question, others would naturally follow without being prompted. Hence we felt that the interview process was on the whole fluid and natural. The use of prompt or probing questions was employed to either stimulate or delve into further discussion.

In term of dependability (reliability), we followed a clear and recognised process including development of an interview framework (which is transparent and clear), alongside use of a lead moderator and separate objective moderator to ensure standardisation. During analysis, we employed a recognised 6 stage model (Braun and Clarke, 2006) for thematic analysis and coding. In setting up the study, we decided to utilise a triangulation approach during coding to ensure rigor and minimise risk of internal bias. This involved initial independent coding and theme development by two members of the research team, and then separately a second-

phase approach to independent coding/ theme development from the third member of the research team to assess both for consistency and internal checking in case anything had been missed.

In addressing confirmability (minimising researcher bias) we employed several approaches to ensure the research team did not unduly bias the process or findings and that the thematic analysis reflected the participants lived experiences and perspectives. This included development and refinement of a focus group question framework, including lead and objective moderator roles and use of non-leading open ended questions. Further, by employing a recognised thematic analysis process (Braun and Clarke, 2006), this minimises opportunity for internal bias. During the coding process we employed a clear triangulation process as previously noted. Furthermore, during the development of themes, we employed open discussion and shared observations within the research team to ensure that previous assumptions based on prior research were not directly influencing the findings. From the results section, we have included direct quotations from participants to fully enrich the nature of the themes presented, highlighting that underlying themes were driven from collective shared experiences and perceptions from the groups.
