# Supplementary material for: Male perceptions and experiences of weight loss initiatives: Considerations for sustainable lifestyle practices
Source: PLoS One. 2026 Aug 26;21(8):e0348158. doi: 10.1371/journal.pone.0348158 (PMC13514000; doi:10.1371/journal.pone.0348158)
Supplement: Supplementary file 2 [file pone.0348158.s001.docx]

**S1 File.**

**Appendix showing general framework for focus group questions.**

**General overview**

Targeting open-ended questions (semi-structured) with flexibility for exploration.

Introductions / warm-up / participant arrival

General welcome to an open environment - basic introductions, made to feel welcome.

When seated offer an introduction to the focus group outlining the order of play, rationale for research and overall goals. Check consent forms completed.

**Section 1 – Views/ opinions on weight management** (~15-20mins pending discussion)

Aim overall to obtain general views and perceptions of weight loss programmes or initiatives

1. What are your views or opinions on weight loss or weight management?

[Aim: to ascertain general views on weight loss initiatives and open up the groups opinions on weight loss strategies specific to men; do they perceive dieting as easy or hard?]. Depending on flow of conversation build in Q2-4 as applicable.

2. Have you personally tried to lose weight in the past?

[Aim: find out who has attempted weight loss programmes before (and which ones) and/or who is looking to lose weight; consider whether the general perception has been positive or negative and why]

3. What type of diets or programmes have you previously tried? What worked? What didn’t?

[Aim: consider factors that may have been perceived as successful and how long programmes were followed]

4. How do you think men in general perceive weight loss initiatives? Do you think men differ to women when it comes to the word diet and dieting?

[Aim: consider if there are male specific aspects that may be pertinent to longer term success]

**Section 2 – Knowledge and understanding around weight loss initiatives** (~15-20mins pending discussion)

Aim overall to obtain experiences of weight loss programmes or initiatives

5. What to you is a successful diet? What aspects made it a successful diet?

[Aim: to understand the groups understanding or knowledge of food/ diet related aspects to a successful outcome; try to ascertain factors impacting successful dietary outcomes or factors impacting positive experiences] Depending on flow of conversation build in Q6-7.

6. What makes an unsuccessful diet?

[Aim: consider if there are any perceived negatives to dietary programmes]

7. Do you think exercise is important for weight loss? What type? How frequently do you think exercise is needed?

[Aim: consider if they perceive exercise to be a key focus within a weight loss initiative and why; what is the understanding behind the importance of exercise?]

8. Learning from the past what would you do differently next time?

[Aim: to see if key learnings come up which may be pertinent for longer term success]

**Section 3 – Critical insights and thoughts towards longer term positive change** (~15-20mins pending discussion)

Aim overall to i) establish ‘moments of realisation’ leading to positive change, and ii) opinions on successful longer term weight loss initiatives.

9. Thinking about your own situation, when did you realise that you needed to lose weight? What was the trigger?

[Aim: consider the point where they realised the importance of needing to lose weight, what was the cause or reason?] Depending on flow of conversation build in Q10-12.

10. What was the motivation to lose weight? When did you make the decision that you going to change?

[Aim: building on previous question, consider factors that led to positive motivation either in the short term or over time – build on key points raised by discussion]

11. What were the challenges faced when undertaking weight loss initiatives?

[Aim: consider barriers to change (food/exercise/lifestyle/family etc.) or factors leading to withdrawal from a program]

12. When thinking about success weight loss programs, what would you say were the key reasons for positive change and your own success?

[Aim: consider factors leading to positive change both internally and externally]

13. What do you think could help other men achieve longer term success in losing and maintaining weight loss?

[Aim: consider key points raised which may support observations for specific longer term success]

14. Are there any other factors you think men look for in a weight loss programme?

[Aim: consider factors such as lifestyle, personal circumstances, personal strategies, technology etc.]

Bring focus group to a close at an appropriate moment, thank the group and explain the next stage of the research study (thematic analysis).
