# Supplementary material for: Male perceptions and experiences of weight loss initiatives: Considerations for sustainable lifestyle practices
Source: PLoS One. 2026 Aug 26;21(8):e0348158. doi: 10.1371/journal.pone.0348158 (PMC13514000; doi:10.1371/journal.pone.0348158)
Supplement: Supplementary file 3 [file pone.0348158.s004.docx]

PONE-D-25-58139

Male perceptions and experiences of weight loss initiatives: recommendations towards sustainable lifestyle practice.

PLOS One

**Authors replies to comments**

**Journal Requirement comments**

1) Please ensure that your manuscript meets PLOS ONE's style requirements, including those for file naming.

**Authors response: Thank you for the information. We have duly looked at both pdfs and fully reviewed/revised the manuscript in alignment with PLOS ONEs style requirements. This has also involved a complete revision for references.**

2) When completing the data availability statement of the submission form, you indicated that you will make your data available on acceptance. We strongly recommend all authors decide on a data sharing plan before acceptance, as the process can be lengthy and hold up publication timelines. Please note that, though access restrictions are acceptable now, your entire data will need to be made freely accessible if your manuscript is accepted for publication. This policy applies to all data except where public deposition would breach compliance with the protocol approved by your research ethics board. If you are unable to adhere to our open data policy, please kindly revise your statement to explain your reasoning and we will seek the editor's input on an exemption. Please be assured that, once you have provided your new statement, the assessment of your exemption will not hold up the peer review process.

**Authors response: Thank you for this information. This statement will be revised during the resubmission process. As this was a qualitative study involving transcription of focus groups, full transcriptions can be made accessible.**

3) Your ethics statement should only appear in the Methods section of your manuscript. If your ethics statement is written in any section besides the Methods, please delete it from any other section.

**Authors response: Thank you for this information. We have removed the information at the end of manuscript and only kept a clear ethics statement in the Methods section as requested.**

4) We note that this data set consists of interview transcripts. Can you please confirm that all participants gave consent for interview transcript to be published?

**Authors response: Yes indeed, all participants provided consent for interview transcripts to be published. There are no potentially identifying information, and where only first names were mentioned, these were removed during the thematic analysis process so that individuals could not be identified in any context.**

5) Please include captions for your Supporting Information files at the end of your manuscript, and update any in-text citations to match accordingly.

**Authors response: Thank you for noting this, we have updated the manuscript accordingly.**

6) If the reviewer comments include a recommendation to cite specific previously published works, please review and evaluate these publications to determine whether they are relevant and should be cited. There is no requirement to cite these works unless the editor has indicated otherwise.

**Author response: we have noted this comment.**

**Response to reviewers:**

**Reviewer #1:** Thank you for giving me the opportunity to review this paper. This study addresses a significant health issue, but there are several key concerns regarding the methodology:

1. Please clarify the specific type of qualitative study and provide justification for this choice.

**Authors response: Firstly thank you for the review and commentary, which presented interesting and beneficial research team discussion. In constructing the paper, we aimed to be as concise as possible in the early stages, knowing that the nature of thematic analysis findings can be longer. In doing so, it is evident that we may have overlooked methodological detail which was indeed evident during the design and execution of the study. As the manuscript has been in submission since October, it has been a while since we have reviewed the paper so this has been a useful exercise in itself.**

**The study employed a qualitative descriptive study format using focus groups to explore mens perceptions and experiences of weight loss initiatives with the intention of informing practices which may be more sustainable and beneficial in the longer term. Based on previous work within our group, we also recognised the challenge faced when recruiting participants particularly when more sensitive topics are presented (such as weight loss). As such use of semi-structured interviews within focus group sessions was deemed appropriate in providing an informal, safe space for participants to openly and honestly engage. Indeed, topics which may be somewhat sensitive to discuss may be more easier to explore when there is peer support or similar shared experiences. The study type was based on previously published research by our group (reference 24) and follows similar guidelines and approaches outlined elsewhere (see: <https://pubmed.ncbi.nlm.nih.gov/40374218/> and** [**https://pmc.ncbi.nlm.nih.gov/articles/PMC10797665/**](https://pmc.ncbi.nlm.nih.gov/articles/PMC10797665/) **). We have revised the beginning of the ‘Focus groups’ section in the methods to reflect revised modifications to the manuscript.**

2. The study setting needs more detail. Please describe the geographic or contextual setting and explain how participants were recruited, what do you mean of via social media or advertisements?
**Authors response: Again, thank you for the comment. It is important to note that we have followed approaches similar to other published articles (**[**https://pubmed.ncbi.nlm.nih.gov/37339012/**](https://pubmed.ncbi.nlm.nih.gov/37339012/) **,** [**https://pubmed.ncbi.nlm.nih.gov/35010838/**](https://pubmed.ncbi.nlm.nih.gov/35010838/) **,** [**https://www.tandfonline.com/doi/full/10.1080/1612197X.2015.1114504**](https://www.tandfonline.com/doi/full/10.1080/1612197X.2015.1114504) **), some of which do not go into as much detail (including one published in this journal:** [**https://journals.plos.org/plosone/article?id=10.1371/journal.pone.0076089**](https://journals.plos.org/plosone/article?id=10.1371/journal.pone.0076089) **). However, in fully responding to this comment, as this was an exploratory study, we aimed to undertake local focus group interviews (specific to the Anglia Region, Cambridgeshire and Chelmsford where we are based in the UK). However, for those who could not attend the University, we also held an online Teams forum for practicality. All face-to-face interviews were held in a wide-spaced meeting room environment on the University campus, often used for general or public meetings. Interviews were undertaken with all participants sat in a circle, facing each other to foster equality, open communication, and active listening throughout. In all interviews members of the research team were also positioned amongst the participants within the circle.**

**Participants were recruited via convenience sampling utilising several approaches, mainly word of mouth and personal contacts, alongside use of social media (researcher promotion via public advert on facebook or Linkedin). The same advert was also promoted via our local University intranet and media network (i.e. study adverts promoted internally via advertising screens and internal university marketing). In addition, study adverts were promoted via local government authority (Cambridgeshire Region) health initiatives following gatekeeper approval. Via adverts we employed purposive and snowballing sampling approaches. We have added content to the methods section to clarify these points.**

3. In terms of participant selection, please provide comprehensive inclusion and exclusion criteria, as the current description is too brief.

**Authors response: The initial information provided in the manuscript is correct based on the general remit employed during the ethical approval process. We purposely wanted to keep the inclusion remit broad so as not to restrict study participation. However, we have included additional information in the revised manuscript to further clarify study inclusion/exclusion in line with overall study conduct.**

**For study inclusion, participants were required to be male, aged > 30 years, in general good health, and able to communicate in English. Participants were also required to have a lived experience of being overweight, having previously undertaken a weight-loss programme or were actively aiming to lose weight in the near future, and therefore willing and able to contribute to discussions around perceptions and experiences of weight loss initiatives within a semi-structured focus group discussion setting. Participants were excluded if they were: < 30 years, had an underlying medical condition that would make participation unsafe or inappropriate, or had no prior engagement with weight loss behaviours. Participants were also excluded if they unwilling to be audio-recorded or unable to provide informed consent.**

4. Regarding sampling, please detail the sampling framework and justify the sample size with relevant references.
**Authors response: Our study employed a criterion-based purposive sampling framework. Participants were selected based on predefined characteristics that were directly aligned with the study aims and inclusion/exclusion criteria. Qualitative studies frequently employ purposeful sampling, a strategy in which participants are chosen based on their capacity to contribute detailed, experience‑based information that aligns closely with the study’s aims (Patton, 2002). Reference:** **Patton, M. 2002. Qualitative research and evaluation methods. 3^rd^ Edition. Sage Publications; Thousand Oaks, CA.**

**Sample size was based on prior experience (**[**https://pubmed.ncbi.nlm.nih.gov/35010838/**](https://pubmed.ncbi.nlm.nih.gov/35010838/)**, incorporating 8 participants), and further justified through reference to numerous publications employing a similar methodological approach (e.g.** [**https://www.mdpi.com/1660-4601/20/4/3320**](https://www.mdpi.com/1660-4601/20/4/3320)**, n=13;** [**https://www.mdpi.com/1660-4601/22/12/1796**](https://www.mdpi.com/1660-4601/22/12/1796)**, 2 FGIs only and n=14;** [**https://link.springer.com/article/10.1186/s12889-024-20677-5**](https://link.springer.com/article/10.1186/s12889-024-20677-5)**, n= 15 adults;** [**https://www.tandfonline.com/doi/pdf/10.1080/17482631.2024.2406099**](https://www.tandfonline.com/doi/pdf/10.1080/17482631.2024.2406099)**, n=10). In the current study, we undertook several focus groups to ascertain theme saturation. The overall included sample size (n=24) is considered robust in alignment with other published research within this domain.**

5. For data collection tools, please explain how the semi-structured interview questions were developed, their number, and whether any pilot testing was conducted.

**Authors response: The semi‑structured interview question framework (see S1 File) was developed to align closely with the primary aim of the study which was to explore male lived experiences, perceptions, motivations, and barriers related to weight‑loss initiatives. The researcher’s questions were developed based on the following:**

- **The existing literature on male engagement with weight‑loss programmes, relevant behavioural change strategies, relevant prevalence / incidence data and gender‑specific barriers.**
- **Theoretical considerations in including hegemonic practices, male help seeking behaviours, reports on lifestyle behaviour change, masculine norms, and both general and specific weight‑management challenges / facilitators.**
- **The research team’s prior work, which highlighted gaps in understanding men’s motivations and experiences with weight loss and management.**

**During initial preparation, the research team discussed what the key objectives and themes were, and drafted open-ended questions to encourage more detailed responses and wider group discussion. Questions were informally piloted with academic colleagues during this initial preparation phase to ensure alignment with the underlying objectives of the study.**

**The resulting focus group framework consisted of a set of pre‑determined open‑ended questions, designed to prompt discussion around dieting experiences, perceived barriers, motivations for change, support mechanisms, and views on long‑term weight‑loss sustainability. The underlying framework comprised 3 general sections in logical order (views/opinions on weight management; knowledge and understanding around weight loss initiatives; and critical insights and thoughts towards longer term positive change), and 14 prompt questions. The researcher’s accounted for the use of additional probing questions to further explore responses to improve depth of understanding, consistent with the semi‑structured approach. Based on the above, we were able to ensure that the questions were grounded in evidence, relevant, and capable of eliciting rich experiential data.**

6. Since the study employed focus group discussions, please justify why this method was chosen, especially considering the sensitive nature of the topic.

**Author response:** **Focus group discussions were selected as the primary data collection method because they enabled the researchers to explore the experiences, social meanings, and the collective narratives surrounding weight loss among men >30 years. The researchers wished to understand male perceptions, motivations, and barriers to weight‑loss engagement. Topics that are shaped not only by individual experiences and beliefs but by social norms, masculine identities, and peer group influences. Focus groups are well-suited to capturing these relational perspectives, as they provide an environment for participants to respond to, challenge, and build upon each other’s perspectives. In doing so these help to reveal collective perspectives, beliefs, experienced and social norms detected via thematic analysis.**

**Based on prior work from this group, we were initially conscious of the sensitive nature of the topic, particularly from a male context which is somewhat under-represented. Indeed, findings from other studies highlight that sensitive topics may often be more freely disclosed or discussed within informal focus group settings where peer support or those having similar experiences become more comfortable to share perspectives and experiences (**[**https://www.tandfonline.com/doi/full/10.1080/13645579.2017.1281601**](https://www.tandfonline.com/doi/full/10.1080/13645579.2017.1281601) **).**

7. Concerning the study procedure, please elaborate on how social media was utilized, the recruitment timeline, the interviewers involved, the language used, and the overall recruitment and interviewing process.

**Author response: As outlined within comment 2, participants were recruited via convenience sampling utilising several approaches, mainly word of mouth and personal contacts, alongside use of social media (researcher promotion via public advert on Facebook or Linkedin)[See comment 2 for further information]. In terms of social media, an online advert depicting the study aims and objectives, participants requirements and what to do next if interested was posted via the researchers own social media networks (e.g. Facebook, LinkedIn). This was part of the strategy to maximise reach within the local region via personal connections. The recruitment timeline was undertaken over a 7 week period preceding start of the first focus group. All researchers were involved in supporting and conducting the focus group interviews. MC is a qualitative public health researcher with considerable experience in this domain, particularly relevant to male participants and lived experienced. As such, MC was the key moderator for each focus group. Both JR and JL are experienced nutrition researchers, with experience in conducting research (including focus groups) pertinent to weight management and male participants. As such, JR acted as an objective moderator to ensure all themes/objectives were addressed. JL supported this role, and employed prompt or probing questions in line with the framework used to encourage further discussion. All focus group interviews were conducted verbally in English. For two of the FGIs, these were held face-to-face within the same University meeting room (purposefully designed to be an informal, relaxed, wide, open-planned setting). The room was set out in an open-planned, circular layout to encourage and facilitate open discussion. Following initial welcome and provision of name badges, the research team checked participants had read and fully understood the ‘Participant Information Sheet’ which overviewed the study in detail, emphasising that participation was voluntary, and that they could withdraw at any point without judgement. Following this, participants were required to complete written informed consent prior to starting the interview itself. All interviews lasted approximately 1hr and were audio recorded using a TileRec Digital USB 3.0 Type C voice activated recorder. The research team utilised the focus group questions framework as a means to provide logical structure and organisation to the interviews.**

8. For data transcription and analysis, please clarify who handled transcription, whether translation was needed, and how potential biases were minimized.

**Author response: in line with the policy of our Institutional ethics committee, when undertaking audio recorded interviews, audio files must be transcribed verbatim using a university-approved service. As such, once all focus groups were completed, all audio files (MP3) were uploaded to a university-approved provider, with word document transcripts provided to the research team following transcription completion. As all interviews were conducted in English, no translation was needed. When reviewing the transcripts, coding was initially conducted by two researchers (MC and JL) with second‑phase confirmation by a third researcher (JR). As such, the use of duplicate coders, along with a second-phase confirmation process was employed as part of a triangulation process to minimise potential bias. Furthermore, by the fourth focus group, similar contexts, perspectives and experiences were noted by the research team, and confirmed during the thematic analysis process inferring that saturation had likely been reached.**

9. Finally, regarding trustworthiness and rigor, please elaborate on how credibility, transferability, dependability, and confirmability were ensured.

**Author response: To address this, we reverted back to early research by Lincoln and Guba where these dimensions are sourced** (**Lincoln, Y.S. and Guba, E.G. (1986), But is it rigorous? Trustworthiness and authenticity in naturalistic evaluation. New Directions for Program Evaluation, 1986: 73-84. https://doi.org/10.1002/ev.1427) .**

**In addressing credibility (internal validity) we employed several focus groups as a means to determine if similar experiences/perceptions were noted amongst the groups. As noted in previous comments, the use of focus groups was also pertinent to allow participants to respond to and build on each other’s ideas, thus helping to reveal collective beliefs and social norms about topics meanwhile stimulating each other’s experiences or perspectives. Focus groups were undertaken in a similar manner, utilising a facilitating lead moderator and objective moderator to ensure neutrality, consistency and rigor. Furthermore, in undertaking the focus groups, we also used confirmation questions or statements to ensure that the information provided was correct and clear. We also standardised the duration of each interview to ensure participants had a similar timeframe and context between focus groups.**

**In addition, we employed a recognised stage process (Braun and Clarke, 2006) to standardise the methodological approach, and used a clear triangulation approach during the thematic analysis coding process to minimise risk of internal bias and ensure multiple opinions were taken on board before arriving at a consensus.**

**In addressing transferability (external validity), we have provided comprehensive information relating to operational set up and delivery of the focus group interviews from context, recruitment/ inclusion/exclusion criteria, interview setting, and development of a focus group question framework. In doing so, our objective is to be as fully transparent in the process as possible for generalisability. We also noted earlier on that we employed a purposive sampling framework, aiming to also capture a range of ages (>30 years), educational backgrounds and marital status (see Table 1 in the manuscript). Within the focus group interviews we did notice that sometimes when one person would initiate a response to a moderator question, others would naturally follow without being prompted. Hence we felt that the interview process was on the whole fluid and natural. The use of prompt or probing questions was employed to either stimulate or delve into further discussion.**

**In term of dependability (reliability), we followed a clear and recognised process including development of an interview framework (which is transparent and clear), alongside use of a lead moderator and separate objective moderator to ensure standardisation. During analysis, we employed a recognised 6 stage model (Braun and Clarke, 2006) for thematic analysis and coding. In setting up the study, we decided to utilise a triangulation approach during coding to ensure rigor and minimise risk of internal bias. This involved initial independent coding and theme development by two members of the research team, and then separately a second-phase approach to independent coding/ theme development from the third member of the research team to assess both for consistency and internal checking in case anything had been missed.**

**In addressing confirmability (minimising researcher bias) we employed several approaches to ensure the research team did not unduly bias the process or findings and that the thematic analysis reflected the participants lived experiences and perspectives. This included development and refinement of a focus group question framework, including lead and objective moderator roles and use of non-leading open ended questions. Further, by employing a recognised thematic analysis process (Braun and Clarke, 2006), this minimises opportunity for internal bias. During the coding process we employed a clear triangulation process as previously noted. Furthermore, during the development of themes, we employed open discussion and shared observations within the research team to ensure that previous assumptions based on prior research were not directly influencing the findings. From the results section, we have included direct quotations from participants to fully enrich the nature of the themes presented, highlighting that underlying themes were driven from collective shared experiences and perceptions from the groups.**
