# Supplementary material for: Male perceptions and experiences of weight loss initiatives: Considerations for sustainable lifestyle practices
Source: PLoS One. 2026 Aug 26;21(8):e0348158. doi: 10.1371/journal.pone.0348158 (PMC13514000; doi:10.1371/journal.pone.0348158)
Supplement: Supplementary file 4 [file pone.0348158.s005.docx]

**PONE-D-25-58139**

**Male perceptions and experiences of weight loss initiatives: recommendations towards sustainable lifestyle practice.**

**PLOS One**

Reviewer #2: The manuscript addresses an important public health issue by exploring male perceptions and experiences regarding weight loss initiatives and sustainable lifestyle practices. The qualitative focus and thematic exploration provide useful insights into male-specific barriers and motivators toward dietary control and weight management. However, several areas require clarification and improvement before publication.

**Authors response: Firstly, thank you for the review and commentary, which provided areas which have indeed improved the manuscript quality and robustness. We have done our best to address all comments in bold below.**

Major Comments

Title Revision: The title is lengthy and somewhat unclear. Consider revising for conciseness and clarity.

Example: Male Perceptions and Experiences of Weight Loss Initiatives: Recommendations for Sustainable Lifestyle Practices.

**Authors response: We have amended the title as recommended and in keeping with the theme of the research. We did play with other titles (e.g. Serial Dieters and Reluctant Changers: Understanding why men disengage from weight loss programmes and what keeps them in) but ended up coming back to this suggestion and have amended the manuscript accordingly to read: Male Perceptions and Experiences of Weight Loss Initiatives: Considerations for Sustainable Lifestyle Practices.**

Abstract

The abstract should clearly state: the study design, sampling technique, method of thematic analysis, and the practical implications of findings.

**Authors response: Thank you for the insight, we have amended the abstract in accordance with these recommendations. In aiming to keep the abstract within word count we agree that these areas needed to be clearer and have therefore re-included them in the abstract.**

“MP3 files transcribed” should be revised grammatically.

**Author response: Thank you, this has been corrected.**

The concluding statement should briefly specify the “three specific support systems” identified.

**Author response: Thank you, this has been corrected.**

Methodology Clarification

Please clarify the participant recruitment strategy and inclusion/exclusion criteria.

**Author response: As noted within the Materials and Methods (Ethical approval and participant recruitment), participants were recruited through convenience sampling utilising multiple methods employing purposive and snowball sampling (line 68-69 – clean version). Recruitment consisted of word of mouth and personal contacts, through to study advertisement promoted locally (institutional intranet and public forum marketing) through to social media (Facebook/LinkedIn) promotion of the study. Additionally, study advertisement was promoted via local government authority health initiatives following gatekeeper approval. This strategy is noted in the methods and led to recruitment of 30 male participants, from which 24 completed the study as noted.**

**Information based on the inclusion/exclusion criteria is detailed in lines 74-84 (clean version) following a similar request from reviewer 1. We purposely wanted to keep the inclusion remit broad so as not to restrict study participation, and based on our ethical approval process. For clarity, participants were required to be male, aged > 30 years, in general good health, and able to communicate in English. Participants were also required to have a lived experience of being overweight, having previously undertaken a weight-loss programme or were actively aiming to lose weight in the near future, and therefore willing and able to contribute to discussions around perceptions and experiences of weight loss initiatives within a semi-structured focus group discussion setting. Participants were excluded if they were: < 30 years, had an underlying medical condition that would make participation unsafe or inappropriate, or had no prior engagement with weight loss behaviours. Participants were also excluded if they unwilling to be audio-recorded or unable to provide informed consent.**

More detail is needed regarding: ethical approval, data saturation, coding procedures, and how trustworthiness/rigor of qualitative findings was ensured.

**Author response:** **Although we felt the summarised information around ethical approval was already clear enough for publication, we have since added further detail specific to the school and institution for clarity (line 66-67 clean version).** **In terms of thematic analysis we followed Braun and Clarkes six stage model as noted in line 133 (clean version). Coding was initially undertaken by 2 researchers independently and manually, with secondary verification from a 3^rd^ researcher as noted in the manuscript. This approach was taken to minimise risk of personal bias, and involved reading transcripts several times independently. Within this strategy, both inductive and deductive coding approaches were employed, and codes clustered leading to initial theme generation. Additional information has been added to the methods. As part of this process we noted that similar themes, perspectives and experiences were occurring by the final focus group with no new codes/themes appearing, and therefore following research team discussion and agreement it was deemed that data saturation had occurred. This is aligned with previously published research (https://pubmed.ncbi.nlm.nih.gov/34785096/).**

**In terms of trustworthiness and rigor, we identified clear areas pertaining to credibility, transferability, dependability and confirmability as outlined by Lincoln and Guba where these dimensions are sourced (Lincoln, Y.S. and Guba, E.G. (1986), But is it rigorous? Trustworthiness and authenticity in naturalistic evaluation. New Directions for Program Evaluation, 1986: 73-84.** [**https://doi.org/10.1002/ev.1427**](https://doi.org/10.1002/ev.1427)**). As such whilst we felt this significantly added to the methods section, we deemed it appropriate to add such contexts for robustness and clarity as follows (line 148 onwards – clean version):**

“Overall rigor and trustworthiness of the qualitative findings were ensured through established criteria. Credibility (internal validity) was addressed by conducting multiple focus groups in a standardised, moderated and neutral manner, using confirmation questions or statements, a recognised analytical process, a triangulation approach during coding and team consensus on theme development. Transferability (external validity) was enhanced through transparent reporting of study procedures, including context, recruitment, inclusion/exclusion criteria, interview settings and development of the interview framework. Dependability (reliability) was demonstrated through use of a clear methodological process, a structured interview framework, inclusion of lead and objective moderators, and a recognised six-stage thematic analysis approach with triangulation to strengthen analytical rigor. Confirmability was achieved by minimising researcher bias through framework refinement using non-leading, open-ended interview questions, strategic moderator roles, and a validated thematic analysis process incorporating triangulation and open team discussion. Direct participant quotations were used to enrich and substantiate themes, demonstrating that findings were driven from collective shared experiences and perceptions rather than researcher assumptions”.

Specify whether thematic analysis followed an established framework such as Braun and Clarke.

**Author response: Yes indeed, this was previously highlighted in line 133 of the methods (clean version) outlining the 6 stages undertaken.**

Sample Characteristics - The sample size of 24 participants should be justified for qualitative inquiry.

**Author response: Sample size was based on prior experience (**[**https://pubmed.ncbi.nlm.nih.gov/35010838/**](https://pubmed.ncbi.nlm.nih.gov/35010838/) **, incorporating 8 participants), and further justified through reference to numerous publications employing a similar methodological approach (e.g.** [**https://www.mdpi.com/1660-4601/20/4/3320**](https://www.mdpi.com/1660-4601/20/4/3320) **, n=13;** [**https://www.mdpi.com/1660-4601/22/12/1796**](https://www.mdpi.com/1660-4601/22/12/1796) **, 2 FGIs only and n=14;** [**https://link.springer.com/article/10.1186/s12889-024-20677-5**](https://link.springer.com/article/10.1186/s12889-024-20677-5) **, n= 15 adults;** [**https://www.tandfonline.com/doi/pdf/10.1080/17482631.2024.2406099**](https://www.tandfonline.com/doi/pdf/10.1080/17482631.2024.2406099) **, n=10). In the current study, we undertook several focus groups to ascertain theme saturation. The overall included sample size (n=24) was therefore considered robust in alignment with other published research within this domain. We have noted this in line 84-85 (clean version) for clarity.**

Provide additional demographic information such as occupation, socioeconomic status, ethnicity, or educational background if available, since these factors may influence perceptions of dieting and lifestyle practices.

**Author response: In line with our Institutional ethics guidelines we could only include data approved for collection. In this case this included age, ethnicity, marital status, highest education level and employment status. We therefore did not collect data on specific occupations and socio-economic status but have noted the rest in table 1 in the methods section. We also note this in the limitations section for clarity.**

Results Presentation

The themes are relevant, but participant quotations should be presented more systematically to strengthen interpretation.

**Author response: Thank you for this comment which prompted team discussion. We were a little unsure what to do here as we have followed a similar format to previously published work. For each section we have introduced the theme/sub-theme and then provided supporting quotations and highlighted key words for clarity. However, in taking on board the reviewers comment here, we have thoroughly revised the results section providing clearer alignment between author commentary and participant quotations to support the theme/sub-theme in question. Additionally, we have attempted to be more structured in quotations in terms of order of appearance across the focus groups to enhance the richness of the information collated. In doing so we feel this has actually enhanced the flow of the results section and therefore wish to thank the reviewer for this comment.**

Consider adding a table summarizing themes, subthemes, and representative quotes.

**Author response: Thank you for this insight. Whilst we felt this over-repeating information at the beginning of the results section, in compiling this table it did actually help to bring together the overall findings so we have included this at the end of the results section as Table 2. Again, thank you for this insight which has indeed benefitted the manuscript.**

Some findings appear descriptive rather than analytical; deeper interpretation is recommended.

**Author response: In reading back through the results section we are in agreement with this comment and have attempted to provide more commentary leading into the quotations whilst keeping aligned with the more descriptive nature of a results section. Furthermore, we have attempted to provide deeper evaluation of the results in the discussion session to address this comment as well.**

Discussion Section

The discussion should better connect findings with existing literature on male health behavior, masculinity, dieting perceptions, and sustainable lifestyle interventions. Expand on why men perceive dieting negatively and how gender norms may influence engagement in weight loss programs. Clarify how the findings contribute new knowledge beyond previous studies.

**Author response: Thank you for this insight. We discussed this point at length within the research team and have reshaped and reformatted the discussion section to accommodate more connection with current literature whilst maintaining themes highlighted from this research. In doing so we feel this has significantly improved the overall flow of the discussion section. There is now more discussion around masculine narratives in line with previously reported literature and male perceptions of dieting including an explanation on why men perceived dieting negatively (and somewhat paradoxically). In building new knowledge we have highlighted the observed ‘aesthetic appeal’ paradox and also note the unique ‘buddy-mentor-spousal’ support interplay which if leveraged effectively could be a potent approach in maximising adherence to weight-loss initiatives.**

Practical Implications

The manuscript mentions “male-specific narratives” and “support systems,” but practical recommendations remain vague. Authors should provide clearer guidance for healthcare professionals, policymakers, or intervention designers.

**Author response: Whilst we did not specifically set out to provide direct guidance for healthcare professionals etc, but instead report observations from a focus group design, we do agree with this comment. As such we have thoroughly revised the discussion section and reformulated our stance on ‘translational considerations’ where we include noted highlights as potential recommendations or guidance for healthcare professionals etc.**

Limitations

The limitations section should explicitly discuss: small sample size, possible selection bias, self-reported experiences, and limited generalizability.

**Author response: Thank you for these insights, which are both valid and helpful. We have adjusted the limitations section to include all of these.**

Minor Comments

Line 13–15: Revise sentence structure for readability.

**Author response: This has now been revised.**

Line 17–18: “Interviews were undertaken by experienced male researchers” could be shortened and clarified.

**Author response: This has now been revised. As we mention that the research team were experienced male researchers in the methods section, we opted to shorten this sentence to read: “**These semi-structured interviews were undertaken using a standardised set of open-ended questions, with additional lines of enquiry employed where appropriate”. **We trust this suffices and is further clarified in the methods section.**

Line 19: “Interviews were recorded with MP3 files transcribed” should be revised to:

“Interviews were audio-recorded and later transcribed verbatim for thematic analysis.”

**Author response: This has now been revised, in accordance with your previous mention in the abstract section above.**

Ensure consistent formatting of units such as kg·m².

**Author response: We have checked the manuscript and the formatting does appear correct and consistent throughout. The dot between kg and m2 is raised as appropriate.**

Proofreading is needed for grammar, punctuation, and spacing throughout the manuscript.

**Author response: Thank you – we have undertaken a full check on grammar, punctuation and spacing and amended any errors where appropriate.**

References should be checked for journal formatting consistency.

**Author response: Thank you – we have undertaken a full check on all references in alignment with the journal requirements. Additionally, multiple references have been added based on revisions to the discussion section noted above.**

Overall Recommendation

The study explores a meaningful and under-researched area regarding male engagement with weight loss initiatives. With stronger methodological clarification, deeper analytical discussion, and improved presentation of findings, the manuscript could make a valuable contribution to the literature on gender-specific health behavior and sustainable lifestyle interventions.

**Author response: we thank you for your time and commitment in reviewing our manuscript and the constructive recommendations made. In amending the manuscript we feel that the comments have subsequently improved the quality and robustness of the manuscript, for which we thank the reviewer.**
