# Supplementary material for: Male perceptions and experiences of weight loss initiatives: Considerations for sustainable lifestyle practices
Source: PLoS One. 2026 Aug 26;21(8):e0348158. doi: 10.1371/journal.pone.0348158 (PMC13514000; doi:10.1371/journal.pone.0348158)
Supplement: Peer review history

# Peer review: Decision Letter 0

**DOI:** 10.1371/journal.pone.0348158.r001

**Authors:** Ilse Bloom

27 Feb 2026

PONE-D-25-58139

Male perceptions and experiences of weight loss initiatives: recommendations towards sustainable lifestyle practice.

PLOS One

Dear Dr. Roberts,

-->Thank you for submitting your manuscript to PLOS ONE. After careful consideration, we feel that it has merit but does not fully meet PLOS ONE’s publication criteria as it currently stands. Therefore, we invite you to submit a revised version of the manuscript that addresses the points raised during the review process.

==============================

Please note that we have only been able to secure a single reviewer to assess your manuscript. We are issuing a decision on your manuscript at this point to prevent further delays in the evaluation of your manuscript. Please be aware that the editor who handles your revised manuscript might find it necessary to invite additional reviewers to assess this work once the revised manuscript is submitted. However, we will aim to proceed on the basis of this single review if possible.

The comments from the reviewer can be found below. Please review these comments and make the appropriate revisions to your manuscript alongside a point-by-point response to the concerns raised.

==============================

Please submit your revised manuscript by Apr 12 2026 11:59PM. If you will need more time than this to complete your revisions, please reply to this message or contact the journal office at plosone@plos.org. When you're ready to submit your revision, log on to https://www.editorialmanager.com/pone/ and select the 'Submissions Needing Revision' folder to locate your manuscript file.

Please include the following items when submitting your revised manuscript:

- A letter that responds to each point raised by the academic editor and reviewer(s). You should upload this letter as a separate file labeled 'Response to Reviewers'.
- A marked-up copy of your manuscript that highlights changes made to the original version. You should upload this as a separate file labeled 'Revised Manuscript with Track Changes'.
- An unmarked version of your revised paper without tracked changes. You should upload this as a separate file labeled 'Manuscript'.

If you would like to make changes to your financial disclosure, please include your updated statement in your cover letter. Guidelines for resubmitting your figure files are available below the reviewer comments at the end of this letter.

If applicable, we recommend that you deposit your laboratory protocols in protocols.io to enhance the reproducibility of your results. Protocols.io assigns your protocol its own identifier (DOI) so that it can be cited independently in the future. For instructions see: https://journals.plos.org/plosone/s/submission-guidelines#loc-laboratory-protocols. Additionally, PLOS ONE offers an option for publishing peer-reviewed Lab Protocol articles, which describe protocols hosted on protocols.io. Read more information on sharing protocols at https://plos.org/protocols?utm_medium=editorial-email&utm_source=authorletters&utm_campaign=protocols.

We look forward to receiving your revised manuscript.

Kind regards,

Ilse Bloom

Staff Editor

PLOS One

**Journal Requirements:**

1. When submitting your revision, we need you to address these additional requirements.

Please ensure that your manuscript meets PLOS ONE's style requirements, including those for file naming. The PLOS ONE style templates can be found at

https://journals.plos.org/plosone/s/file?id=wjVg/PLOSOne_formatting_sample_main_body.pdf and

https://journals.plos.org/plosone/s/file?id=ba62/PLOSOne_formatting_sample_title_authors_affiliations.pdf

2. When completing the data availability statement of the submission form, you indicated that you will make your data available on acceptance. We strongly recommend all authors decide on a data sharing plan before acceptance, as the process can be lengthy and hold up publication timelines. Please note that, though access restrictions are acceptable now, your entire data will need to be made freely accessible if your manuscript is accepted for publication. This policy applies to all data except where public deposition would breach compliance with the protocol approved by your research ethics board. If you are unable to adhere to our open data policy, please kindly revise your statement to explain your reasoning and we will seek the editor's input on an exemption. Please be assured that, once you have provided your new statement, the assessment of your exemption will not hold up the peer review process.

3. Your ethics statement should only appear in the Methods section of your manuscript. If your ethics statement is written in any section besides the Methods, please delete it from any other section.

4. We note that this data set consists of interview transcripts. Can you please confirm that all participants gave consent for interview transcript to be published?

If they DID provide consent for these transcripts to be published, please also confirm that the transcripts do not contain any potentially identifying information (or let us know if the participants consented to having their personal details published and made publicly available). We consider the following details to be identifying information:

- Names, nicknames, and initials

- Age more specific than round numbers

- GPS coordinates, physical addresses, IP addresses, email addresses

- Information in small sample sizes (e.g. 40 students from X class in X year at X university)

- Specific dates (e.g. visit dates, interview dates)

- ID numbers

Or, if the participants DID NOT provide consent for these transcripts to be published:

- Provide a de-identified version of the data or excerpts of interview responses

- Provide information regarding how these transcripts can be accessed by researchers who meet the criteria for access to confidential data, including:

a) the grounds for restriction

b) the name of the ethics committee, Institutional Review Board, or third-party organization that is imposing sharing restrictions on the data

c) a non-author, institutional point of contact that is able to field data access queries, in the interest of maintaining long-term data accessibility.

d) Any relevant data set names, URLs, DOIs, etc. that an independent researcher would need in order to request your minimal data set.

For further information on sharing data that contains sensitive participant information, please see: https://journals.plos.org/plosone/s/data-availability#loc-human-research-participant-data-and-other-sensitive-data

If there are ethical, legal, or third-party restrictions upon your dataset, you must provide all of the following details (https://journals.plos.org/plosone/s/data-availability#loc-acceptable-data-access-restrictions):

a) A complete description of the dataset

b) The nature of the restrictions upon the data (ethical, legal, or owned by a third party) and the reasoning behind them

c) The full name of the body imposing the restrictions upon your dataset (ethics committee, institution, data access committee, etc)

d) If the data are owned by a third party, confirmation of whether the authors received any special privileges in accessing the data that other researchers would not have

e) Direct, non-author contact information (preferably email) for the body imposing the restrictions upon the data, to which data access requests can be sent

5. Please include captions for your Supporting Information files at the end of your manuscript, and update any in-text citations to match accordingly. Please see our Supporting Information guidelines for more information: http://journals.plos.org/plosone/s/supporting-information.

6. If the reviewer comments include a recommendation to cite specific previously published works, please review and evaluate these publications to determine whether they are relevant and should be cited. There is no requirement to cite these works unless the editor has indicated otherwise.

[Note: HTML markup is below. Please do not edit.]

Reviewers' comments:

Reviewer's Responses to Questions

**Comments to the Author**

1. Is the manuscript technically sound, and do the data support the conclusions?

The manuscript must describe a technically sound piece of scientific research with data that supports the conclusions. Experiments must have been conducted rigorously, with appropriate controls, replication, and sample sizes. The conclusions must be drawn appropriately based on the data presented.

Reviewer #1: Partly

**********

2. Has the statistical analysis been performed appropriately and rigorously?

Reviewer #1: N/A

**********

3. Have the authors made all data underlying the findings in their manuscript fully available?

The PLOS Data policy requires authors to make all data underlying the findings described in their manuscript fully available without restriction, with rare exception (please refer to the Data Availability Statement in the manuscript PDF file). The data should be provided as part of the manuscript or its supporting information, or deposited to a public repository. For example, in addition to summary statistics, the data points behind means, medians and variance measures should be available. If there are restrictions on publicly sharing data—e.g. participant privacy or use of data from a third party—those must be specified.

Reviewer #1: Yes

**********

4. Is the manuscript presented in an intelligible fashion and written in standard English?

PLOS ONE does not copyedit accepted manuscripts, so the language in submitted articles must be clear, correct, and unambiguous. Any typographical or grammatical errors should be corrected at revision, so please note any specific errors here.

Reviewer #1: Yes

**********

5. Review Comments to the Author

Please use the space provided to explain your answers to the questions above. You may also include additional comments for the author, including concerns about dual publication, research ethics, or publication ethics. (Please upload your review as an attachment if it exceeds 20,000 characters)

**Reviewer #1:** Thank you for giving me the opportunity to review this paper. This study addresses a significant health issue, but there are several key concerns regarding the methodology:

1. Please clarify the specific type of qualitative study and provide justification for this choice.

2. The study setting needs more detail. Please describe the geographic or contextual setting and explain how participants were recruited, what do you mean of via social media or advertisements?

3. In terms of participant selection, please provide comprehensive inclusion and exclusion criteria, as the current description is too brief.

4. Regarding sampling, please detail the sampling framework and justify the sample size with relevant references.

5. For data collection tools, please explain how the semi-structured interview questions were developed, their number, and whether any pilot testing was conducted.

6. Since the study employed focus group discussions, please justify why this method was chosen, especially considering the sensitive nature of the topic.

7. Concerning the study procedure, please elaborate on how social media was utilized, the recruitment timeline, the interviewers involved, the language used, and the overall recruitment and interviewing process.

8. For data transcription and analysis, please clarify who handled transcription, whether translation was needed, and how potential biases were minimized.

9. Finally, regarding trustworthiness and rigor, please elaborate on how credibility, transferability, dependability, and confirmability were ensured.

**********

6. PLOS authors have the option to publish the peer review history of their article (what does this mean?). If published, this will include your full peer review and any attached files.

If you choose “no”, your identity will remain anonymous but your review may still be made public.

**Do you want your identity to be public for this peer review?**  For information about this choice, including consent withdrawal, please see our Privacy Policy.

Reviewer #1: **Yes:** Masoud Mohammadnezhad

**********

[NOTE: If reviewer comments were submitted as an attachment file, they will be attached to this email and accessible via the submission site. Please log into your account, locate the manuscript record, and check for the action link "View Attachments". If this link does not appear, there are no attachment files.]

To ensure your figures meet our technical requirements, please review our figure guidelines: https://journals.plos.org/plosone/s/figures

You may also use PLOS’s free figure tool, NAAS, to help you prepare publication quality figures: https://journals.plos.org/plosone/s/figures#loc-tools-for-figure-preparation.

NAAS will assess whether your figures meet our technical requirements by comparing each figure against our figure specifications.

# Author comment: Author response to Decision Letter 1

**DOI:** 10.1371/journal.pone.0348158.r002

16 Mar 2026

Thank you for the email dated 27th February outline editorial and reviewer revisions for PONE-D-25-58139

Male perceptions and experiences of weight loss initiatives: recommendations towards sustainable lifestyle practice.

PLOS One

Outlined below are the full comments to all requests:

Authors replies to comments

Journal Requirement comments

1) Please ensure that your manuscript meets PLOS ONE's style requirements, including those for file naming.

Authors response: Thank you for the information. We have duly looked at both pdfs and fully reviewed/revised the manuscript in alignment with PLOS ONEs style requirements. This has also involved a complete revision for references.

2) When completing the data availability statement of the submission form, you indicated that you will make your data available on acceptance. We strongly recommend all authors decide on a data sharing plan before acceptance, as the process can be lengthy and hold up publication timelines. Please note that, though access restrictions are acceptable now, your entire data will need to be made freely accessible if your manuscript is accepted for publication. This policy applies to all data except where public deposition would breach compliance with the protocol approved by your research ethics board. If you are unable to adhere to our open data policy, please kindly revise your statement to explain your reasoning and we will seek the editor's input on an exemption. Please be assured that, once you have provided your new statement, the assessment of your exemption will not hold up the peer review process.

Authors response: Thank you for this information. This statement will be revised during the resubmission process. As this was a qualitative study involving transcription of focus groups, full transcriptions can be made accessible.

3) Your ethics statement should only appear in the Methods section of your manuscript. If your ethics statement is written in any section besides the Methods, please delete it from any other section.

Authors response: Thank you for this information. We have removed the information at the end of manuscript and only kept a clear ethics statement in the Methods section as requested.

4) We note that this data set consists of interview transcripts. Can you please confirm that all participants gave consent for interview transcript to be published?

Authors response: Yes indeed, all participants provided consent for interview transcripts to be published. There are no potentially identifying information, and where only first names were mentioned, these were removed during the thematic analysis process so that individuals could not be identified in any context.

5) Please include captions for your Supporting Information files at the end of your manuscript, and update any in-text citations to match accordingly.

Authors response: Thank you for noting this, we have updated the manuscript accordingly.

6) If the reviewer comments include a recommendation to cite specific previously published works, please review and evaluate these publications to determine whether they are relevant and should be cited. There is no requirement to cite these works unless the editor has indicated otherwise.

Author response: we have noted this comment.

Response to reviewers:

Reviewer #1: Thank you for giving me the opportunity to review this paper. This study addresses a significant health issue, but there are several key concerns regarding the methodology:

1. Please clarify the specific type of qualitative study and provide justification for this choice.

Authors response: Firstly thank you for the review and commentary, which presented interesting and beneficial research team discussion. In constructing the paper, we aimed to be as concise as possible in the early stages, knowing that the nature of thematic analysis findings can be longer. In doing so, it is evident that we may have overlooked methodological detail which was indeed evident during the design and execution of the study. As the manuscript has been in submission since October, it has been a while since we have reviewed the paper so this has been a useful exercise in itself.

The study employed a qualitative descriptive study format using focus groups to explore mens perceptions and experiences of weight loss initiatives with the intention of informing practices which may be more sustainable and beneficial in the longer term. Based on previous work within our group, we also recognised the challenge faced when recruiting participants particularly when more sensitive topics are presented (such as weight loss). As such use of semi-structured interviews within focus group sessions was deemed appropriate in providing an informal, safe space for participants to openly and honestly engage. Indeed, topics which may be somewhat sensitive to discuss may be more easier to explore when there is peer support or similar shared experiences. The study type was based on previously published research by our group (reference 24) and follows similar guidelines and approaches outlined elsewhere (see: https://pubmed.ncbi.nlm.nih.gov/40374218/ and https://pmc.ncbi.nlm.nih.gov/articles/PMC10797665/ ). We have revised the beginning of the ‘Focus groups’ section in the methods to reflect revised modifications to the manuscript.

2. The study setting needs more detail. Please describe the geographic or contextual setting and explain how participants were recruited, what do you mean of via social media or advertisements?

Authors response: Again, thank you for the comment. It is important to note that we have followed approaches similar to other published articles (https://pubmed.ncbi.nlm.nih.gov/37339012/ , https://pubmed.ncbi.nlm.nih.gov/35010838/ , https://www.tandfonline.com/doi/full/10.1080/1612197X.2015.1114504 ), some of which do not go into as much detail (including one published in this journal: https://journals.plos.org/plosone/article?id=10.1371/journal.pone.0076089 ). However, in fully responding to this comment, as this was an exploratory study, we aimed to undertake local focus group interviews (specific to the Anglia Region, Cambridgeshire and Chelmsford where we are based in the UK). However, for those who could not attend the University, we also held an online Teams forum for practicality. All face-to-face interviews were held in a wide-spaced meeting room environment on the University campus, often used for general or public meetings. Interviews were undertaken with all participants sat in a circle, facing each other to foster equality, open communication, and active listening throughout. In all interviews members of the research team were also positioned amongst the participants within the circle.

Participants were recruited via convenience sampling utilising several approaches, mainly word of mouth and personal contacts, alongside use of social media (researcher promotion via public advert on facebook or Linkedin). The same advert was also promoted via our local University intranet and media network (i.e. study adverts promoted internally via advertising screens and internal university marketing). In addition, study adverts were promoted via local government authority (Cambridgeshire Region) health initiatives following gatekeeper approval. Via adverts we employed purposive and snowballing sampling approaches. We have added content to the methods section to clarify these points.

3. In terms of participant selection, please provide comprehensive inclusion and exclusion criteria, as the current description is too brief.

Authors response: The initial information provided in the manuscript is correct based on the general remit employed during the ethical approval process. We purposely wanted to keep the inclusion remit broad so as not to restrict study participation. However, we have included additional information in the revised manuscript to further clarify study inclusion/exclusion in line with overall study conduct.

For study inclusion, participants were required to be male, aged > 30 years, in general good health, and able to communicate in English. Participants were also required to have a lived experience of being overweight, having previously undertaken a weight-loss programme or were actively aiming to lose weight in the near future, and therefore willing and able to contribute to discussions around perceptions and experiences of weight loss initiatives within a semi-structured focus group discussion setting. Participants were excluded if they were: < 30 years, had an underlying medical condition that would make participation unsafe or inappropriate, or had no prior engagement with weight loss behaviours. Participants were also excluded if they unwilling to be audio-recorded or unable to provide informed consent.

4. Regarding sampling, please detail the sampling framework and justify the sample size with relevant references.

Authors response: Our study employed a criterion-based purposive sampling framework. Participants were selected based on predefined characteristics that were directly aligned with the study aims and inclusion/exclusion criteria. Qualitative studies frequently employ purposeful sampling, a strategy in which participants are chosen based on their capacity to contribute detailed, experience‑based information that aligns closely with the study’s aims (Patton, 2002). Reference: Patton, M. 2002. Qualitative research and evaluation methods. 3rd Edition. Sage Publications; Thousand Oaks, CA.

Sample size was based on prior experience (https://pubmed.ncbi.nlm.nih.gov/35010838/, incorporating 8 participants), and further justified through reference to numerous publications employing a similar methodological approach (e.g. https://www.mdpi.com/1660-4601/20/4/3320, n=13; https://www.mdpi.com/1660-4601/22/12/1796, 2 FGIs only and n=14; https://link.springer.com/article/10.1186/s12889-024-20677-5, n= 15 adults; https://www.tandfonline.com/doi/pdf/10.1080/17482631.2024.2406099, n=10). In the current study, we undertook several focus groups to ascertain theme saturation. The overall included sample size (n=24) is considered robust in alignment with other published research within this domain.

5. For data collection tools, please explain how the semi-structured interview questions were developed, their number, and whether any pilot testing was conducted.

Authors response: The semi‑structured interview question framework (see S1 File) was developed to align closely with the primary aim of the study which was to explore male lived experiences, perceptions, motivations, and barriers related to weight‑loss initiatives. The researcher’s questions were developed based on the following:

• The existing literature on male engagement with weight‑loss programmes, relevant behavioural change strategies, relevant prevalence / incidence data and gender‑specific barriers.

• Theoretical considerations in including hegemonic practices, male help seeking behaviours, reports on lifestyle behaviour change, masculine norms, and both general and specific weight‑management challenges / facilitators.

• The research team’s prior work, which highlighted gaps in understanding men’s motivations and experiences with weight loss and management.

During initial preparation, the research team discussed what the key objectives and themes were, and drafted open-ended questions to encourage more detailed responses and wider group discussion. Questions were informally piloted with academic colleagues during this initial preparation phase to ensure alignment with the underlying objectives of the study.

The resulting focus group framework consisted of a set of pre‑determined open‑ended questions, designed to prompt discussion around dieting experiences, perceived barriers, motivations for change, support mechanisms, and views on long‑term weight‑loss sustainability. The underlying framework comprised 3 general sections in logical order (views/opinions on weight management; knowledge and understanding around weight loss initiatives; and critical insights and thoughts towards longer term positive change), and 14 prompt questions. The researcher’s accounted for the use of additional probing questions to further explore responses to improve depth of understanding, consistent with the semi‑structured approach. Based on the above, we were able to ensure that the questions were grounded in evidence, relevant, and capable of eliciting rich experiential data.

6. Since the study employed focus group discussions, please justify why this method was chosen, especially considering the sensitive nature of the topic.

Author response: Focus group discussions were selected as the primary data collection method because they enabled the researchers to explore the experiences, social meanings, and the collective narratives surrounding weight loss among men >30 years. The researchers wished to understand male perceptions, motivations, and barriers to weight‑loss engagement. Topics that are shaped not only by individual experiences and beliefs but by social norms, masculine identities, and peer group influences. Focus groups are well-suited to capturing these relational perspectives, as they provide an environment for participants to respond to, challenge, and build upon each other’s perspectives. In doing so these help to reveal collective perspectives, beliefs, experienced and social norms detected via thematic analysis.

Based on prior work from this group, we were initially conscious of the sensitive nature of the topic, particularly from a male context which is somewhat under-represented. Indeed, findings from other studies highlight that sensitive topics may often be more freely disclosed or discussed within informal focus group settings where peer support or those having similar experiences become more comfortable to share perspectives and experiences (https://www.tandfonline.com/doi/full/10.1080/13645579.2017.1281601 ).

7. Concerning the study procedure, please elaborate on how social media was utilized, the recruitment timeline, the interviewers involved, the language used, and the overall recruitment and interviewing process.

Author response: As outlined within comment 2, participants were recruited via convenience sampling utilising several approaches, mainly word of mouth and personal contacts, alongside use of social media (researcher promotion via public advert on Facebook or Linkedin)[See comment 2 for further information]. In terms of social media, an online advert depicting the study aims and objectives, participants requirements and what to do next if interested was posted via the researchers own social media networks (e.g. Facebook, LinkedIn). This was part of the strategy to maximise reach within the local region via personal connections. The recruitment timeline was undertaken over a 7 week period preceding start of the first focus group. All researchers were involved in supporting and conducting the focus group interviews. MC is a qualitative public health researcher with considerable experience in this domain, particularly relevant to male participants and lived experienced. As such, MC was the key moderator for each focus group. Both JR and JL are experienced nutrition researchers, with experience in conducting research (including focus groups) pertinent to weight management and male participants. As such, JR acted as an objective moderator to ensure all themes/objectives were addressed. JL supported this role, and employed prompt or probing questions in line with the framework used to encourage further discussion. All focus group interviews were conducted verbally in English. For two of the FGIs, these were held face-to-face within the same University meeting room (purposefully designed to be an informal, relaxed, wide, open-planned setting). The room was set out in an open-planned, circular layout to encourage and facilitate open discussion. Following initial welcome and provision of name badges, the research team checked participants had read and fully understood the ‘Participant Information Sheet’ which overviewed the study in detail, emphasising that participation was voluntary, and that they could withdraw at any point without judgement. Following this, participants were required to complete written

Attachment Submitted filename: Response to Reviewers.docx

# Peer review: Decision Letter 1

**DOI:** 10.1371/journal.pone.0348158.r003

**Authors:** Ayodeji Oginni

2 Jun 2026

PONE-D-25-58139R1

Male perceptions and experiences of weight loss initiatives: recommendations towards sustainable lifestyle practice.

PLOS One

Dear Dr. Roberts,

Thank you for submitting your manuscript to PLOS ONE. After careful consideration, we feel that it has merit but does not fully meet PLOS ONE’s publication criteria as it currently stands. Therefore, we invite you to submit a revised version of the manuscript that addresses the points raised during the review process.

Please respond to the reviewer's comments and make the necessary revisions to the manuscript accordingly. With respect to the manuscript title, you may revise it as suggested, provided that the revised title remains consistent with the original intent and scope of the study.

Please submit your revised manuscript by Jul 17 2026 11:59PM. If you will need more time than this to complete your revisions, please reply to this message or contact the journal office at plosone@plos.org. When you're ready to submit your revision, log on to https://www.editorialmanager.com/pone/ and select the 'Submissions Needing Revision' folder to locate your manuscript file.

Please include the following items when submitting your revised manuscript:

- A letter that responds to each point raised by the academic editor and reviewer(s). You should upload this letter as a separate file labeled 'Response to Reviewers'.
- A marked-up copy of your manuscript that highlights changes made to the original version. You should upload this as a separate file labeled 'Revised Manuscript with Track Changes'.
- An unmarked version of your revised paper without tracked changes. You should upload this as a separate file labeled 'Manuscript'.

If you would like to make changes to your financial disclosure, please include your updated statement in your cover letter. Guidelines for resubmitting your figure files are available below the reviewer comments at the end of this letter.

If applicable, we recommend that you deposit your laboratory protocols in protocols.io to enhance the reproducibility of your results. Protocols.io assigns your protocol its own identifier (DOI) so that it can be cited independently in the future. For instructions see: https://journals.plos.org/plosone/s/submission-guidelines#loc-laboratory-protocols. Additionally, PLOS ONE offers an option for publishing peer-reviewed Lab Protocol articles, which describe protocols hosted on protocols.io. Read more information on sharing protocols at https://plos.org/protocols?utm_medium=editorial-email&utm_source=authorletters&utm_campaign=protocols.

As the corresponding author, your ORCID iD is verified in the submission system and will appear in the published article. PLOS supports the use of ORCID, and we encourage all coauthors to register for an ORCID iD and use it as well. Please encourage your coauthors to verify their ORCID iD within the submission system before final acceptance, as unverified ORCID iDs will not appear in the published article. *Only*  the individual author can complete the verification step; PLOS staff *cannot*  verify ORCID iDs on behalf of authors.

We look forward to receiving your revised manuscript.

Kind regards,

Ayodeji Babatunde Oginni

Academic Editor

PLOS One

Journal Requirements:

If the reviewer comments include a recommendation to cite specific previously published works, please review and evaluate these publications to determine whether they are relevant and should be cited. There is no requirement to cite these works unless the editor has indicated otherwise.

Please review your reference list to ensure that it is complete and correct. If you have cited papers that have been retracted, please include the rationale for doing so in the manuscript text, or remove these references and replace them with relevant current references. Any changes to the reference list should be mentioned in the rebuttal letter that accompanies your revised manuscript. If you need to cite a retracted article, indicate the article’s retracted status in the References list and also include a citation and full reference for the retraction notice.

[Note: HTML markup is below. Please do not edit.]

Reviewers' comments:

Reviewer's Responses to Questions

**Comments to the Author**

1. If the authors have adequately addressed your comments raised in a previous round of review and you feel that this manuscript is now acceptable for publication, you may indicate that here to bypass the “Comments to the Author” section, enter your conflict of interest statement in the “Confidential to Editor” section, and submit your "Accept" recommendation.

Reviewer #2: (No Response)

**********

2. Is the manuscript technically sound, and do the data support the conclusions?

The manuscript must describe a technically sound piece of scientific research with data that supports the conclusions. Experiments must have been conducted rigorously, with appropriate controls, replication, and sample sizes. The conclusions must be drawn appropriately based on the data presented.

Reviewer #2: Yes

**********

3. Has the statistical analysis been performed appropriately and rigorously?

Reviewer #2: Yes

**********

4. Have the authors made all data underlying the findings in their manuscript fully available?

The PLOS Data policy requires authors to make all data underlying the findings described in their manuscript fully available without restriction, with rare exception (please refer to the Data Availability Statement in the manuscript PDF file). The data should be provided as part of the manuscript or its supporting information, or deposited to a public repository. For example, in addition to summary statistics, the data points behind means, medians and variance measures should be available. If there are restrictions on publicly sharing data—e.g. participant privacy or use of data from a third party—those must be specified.

Reviewer #2: Yes

**********

5. Is the manuscript presented in an intelligible fashion and written in standard English?

PLOS ONE does not copyedit accepted manuscripts, so the language in submitted articles must be clear, correct, and unambiguous. Any typographical or grammatical errors should be corrected at revision, so please note any specific errors here.

Reviewer #2: Yes

**********

6. Review Comments to the Author

Please use the space provided to explain your answers to the questions above. You may also include additional comments for the author, including concerns about dual publication, research ethics, or publication ethics. (Please upload your review as an attachment if it exceeds 20,000 characters)

Reviewer #2: The manuscript addresses an important public health issue by exploring male perceptions and experiences regarding weight loss initiatives and sustainable lifestyle practices. The qualitative focus and thematic exploration provide useful insights into male-specific barriers and motivators toward dietary control and weight management. However, several areas require clarification and improvement before publication.

Major Comments

Title Revision

The title is lengthy and somewhat unclear. Consider revising for conciseness and clarity.

Example: Male Perceptions and Experiences of Weight Loss Initiatives: Recommendations for Sustainable Lifestyle Practices.

Abstract

The abstract should clearly state:

the study design,

sampling technique,

method of thematic analysis,

and the practical implications of findings.

“MP3 files transcribed” should be revised grammatically.

The concluding statement should briefly specify the “three specific support systems” identified.

Methodology Clarification

Please clarify the participant recruitment strategy and inclusion/exclusion criteria.

More detail is needed regarding:

ethical approval,

data saturation,

coding procedures,

and how trustworthiness/rigor of qualitative findings was ensured.

Specify whether thematic analysis followed an established framework such as Braun and Clarke.

Sample Characteristics

The sample size of 24 participants should be justified for qualitative inquiry.

Provide additional demographic information such as occupation, socioeconomic status, ethnicity, or educational background if available, since these factors may influence perceptions of dieting and lifestyle practices.

Results Presentation

The themes are relevant, but participant quotations should be presented more systematically to strengthen interpretation.

Consider adding a table summarizing themes, subthemes, and representative quotes.

Some findings appear descriptive rather than analytical; deeper interpretation is recommended.

Discussion Section

The discussion should better connect findings with existing literature on male health behavior, masculinity, dieting perceptions, and sustainable lifestyle interventions.

Expand on why men perceive dieting negatively and how gender norms may influence engagement in weight loss programs.

Clarify how the findings contribute new knowledge beyond previous studies.

Practical Implications

The manuscript mentions “male-specific narratives” and “support systems,” but practical recommendations remain vague.

Authors should provide clearer guidance for healthcare professionals, policymakers, or intervention designers.

Limitations

The limitations section should explicitly discuss:

small sample size,

possible selection bias,

self-reported experiences,

and limited generalizability.

Minor Comments

Line 13–15: Revise sentence structure for readability.

Line 17–18: “Interviews were undertaken by experienced male researchers” could be shortened and clarified.

Line 19: “Interviews were recorded with MP3 files transcribed” should be revised to:

“Interviews were audio-recorded and later transcribed verbatim for thematic analysis.”

Ensure consistent formatting of units such as kg·m².

Proofreading is needed for grammar, punctuation, and spacing throughout the manuscript.

References should be checked for journal formatting consistency.

Overall Recommendation

The study explores a meaningful and under-researched area regarding male engagement with weight loss initiatives. With stronger methodological clarification, deeper analytical discussion, and improved presentation of findings, the manuscript could make a valuable contribution to the literature on gender-specific health behavior and sustainable lifestyle interventions.

**********

7. PLOS authors have the option to publish the peer review history of their article (what does this mean?). If published, this will include your full peer review and any attached files.

If you choose “no”, your identity will remain anonymous but your review may still be made public.

**Do you want your identity to be public for this peer review?**  For information about this choice, including consent withdrawal, please see our Privacy Policy.

Reviewer #2: No

**********

[NOTE: If reviewer comments were submitted as an attachment file, they will be attached to this email and accessible via the submission site. Please log into your account, locate the manuscript record, and check for the action link "View Attachments". If this link does not appear, there are no attachment files.]

To ensure your figures meet our technical requirements, please review our figure guidelines: https://journals.plos.org/plosone/s/figures

You may also use PLOS’s free figure tool, NAAS, to help you prepare publication quality figures: https://journals.plos.org/plosone/s/figures#loc-tools-for-figure-preparation.

NAAS will assess whether your figures meet our technical requirements by comparing each figure against our figure specifications.

Attachment Submitted filename: PONE-D-25-58139_R1 (1).pdf

# Author comment: Author response to Decision Letter 2

**DOI:** 10.1371/journal.pone.0348158.r004

3 Jul 2026

Please see attached response to reviewer letter which details all revisions made. Responses to reviewer 1 have previously been addressed and are incorporated within current version of the manuscript.

Attachment Submitted filename: Response to Reviewer 2.docx

# Editor report: Decision Letter 2

**DOI:** 10.1371/journal.pone.0348158.r005

**Authors:** Ayodeji Oginni

22 Jul 2026

Male Perceptions and Experiences of Weight Loss Initiatives: Considerations for Sustainable Lifestyle Practices.

PONE-D-25-58139R2

Dear Dr. Roberts,

We’re pleased to inform you that your manuscript has been judged scientifically suitable for publication and will be formally accepted for publication once it meets all outstanding technical requirements.

Within one week, you’ll receive an e-mail detailing the required amendments. When these have been addressed, you’ll receive a formal acceptance letter, and your manuscript will be scheduled for publication.

An invoice will be generated when your article is formally accepted. Please note that if your institution has a publishing partnership with PLOS and your article meets the relevant criteria, all or part of your publication costs will be covered. Please make sure your user information is up-to-date by logging into Editorial Manager at Editorial Manager® and clicking the ‘Update My Information' link at the top of the page. For questions related to billing, please contact billing support.

If your institution or institutions have a press office, please notify them about your upcoming paper to help maximize its impact. If they’ll be preparing press materials, please inform our press team as soon as possible -- no later than 48 hours after receiving the formal acceptance. Your manuscript will remain under strict press embargo until 2 pm Eastern Time on the date of publication. For more information, please contact onepress@plos.org.

Kind regards,

Ayodeji Babatunde Oginni

Academic Editor

PLOS One

Additional Editor Comments (optional):

Reviewers' comments:

# Editor report: Acceptance letter

**DOI:** 10.1371/journal.pone.0348158.r006

**Authors:** Ayodeji Oginni

PONE-D-25-58139R2

PLOS One

Dear Dr. Roberts,

I'm pleased to inform you that your manuscript has been deemed suitable for publication in PLOS One. Congratulations! Your manuscript is now being handed over to our production team.

At this stage, our production department will prepare your paper for publication. This includes ensuring the following:

* All references, tables, and figures are properly cited

* All relevant supporting information is included in the manuscript submission,

* There are no issues that prevent the paper from being properly typeset

You will receive further instructions from the production team, including instructions on how to review your proof when it is ready. Please keep in mind that we are working through a large volume of accepted articles, so please give us a few days to review your paper and let you know the next and final steps.

Lastly, if your institution or institutions have a press office, please let them know about your upcoming paper now to help maximize its impact. If they'll be preparing press materials, please inform our press team within the next 48 hours. Your manuscript will remain under strict press embargo until 2 pm Eastern Time on the date of publication. For more information, please contact onepress@plos.org.

You will receive an invoice from PLOS for your publication fee after your manuscript has reached the completed accept phase. If you receive an email requesting payment before acceptance or for any other service, this may be a phishing scheme. Learn how to identify phishing emails and protect your accounts at https://explore.plos.org/phishing.

If we can help with anything else, please email us at customercare@plos.org.

Thank you for submitting your work to PLOS ONE and supporting open access.

Kind regards,

PLOS ONE Editorial Office Staff

on behalf of

Ayodeji Babatunde Oginni

Academic Editor

PLOS One
